# Characterizing Organic Gunshot Residues with Low-Frequency Raman and Terahertz Vibrational Spectroscopies

**DOI:** 10.1021/acsomega.5c10754

**Published:** 2026-01-23

**Authors:** Salvatore Zarrella, Margaret P. Davis, Mary N. Boyden, Timothy M. Korter

**Affiliations:** Department of Chemistry, 2029 Syracuse University, 3-014 Center for Science and Technology, Syracuse, New York 13244-4100, United States

## Abstract

Low-frequency (10–300 cm^–1^) vibrational spectroscopy is a promising method for enhancing the detection of solid-state organic gunshot residues (OGSRs) which serve as vital trace evidence in crime scene investigations. The use of low-frequency Raman spectroscopy (LFRS) and terahertz time-domain spectroscopy (THz-TDS) allows for the measurement of material-specific lattice vibrations that originate not only from individual molecules, but also from motions between molecules in the solid-state. Together, these vibrations yield unique spectral profiles for compound recognition and characterization. In this study, LFRS and THz-TDS data for two common and structurally similar OGSRs are presented and analyzed: 1,3-diethyl-1,3-diphenylurea (ethyl centralite) and 1,3-dimethyl-1,3-diphenylurea (methyl centralite). Despite their similarities, both exhibit distinctive Raman and THz spectra, and the data have been interpreted using solid-state density functional theory simulations. The computational results show that the vibrations in these molecular crystals that lead to the strongest spectral features all involve torsional motions of the phenyl rings rather than intermolecular motions, such as translations. To demonstrate the ability of LFRS and THz-TDS to differentiate between OGSRs, measurements were also made of binary mixtures of ethyl centralite and methyl centralite. For these specific OGSRs, LFRS was found to be the more sensitive technique with peaks at 98.8 cm^–1^ (ethyl centralite) and 111.7 cm^–1^ (methyl centralite) that are suitable for reliable detection and quantification. These spectral features should serve as practical markers in future analytical studies of alkylated diphenylurea compounds, while the overall approach highlights the unexplored potential of low-frequency vibrational spectroscopy in forensic science.

## Graphical abstract

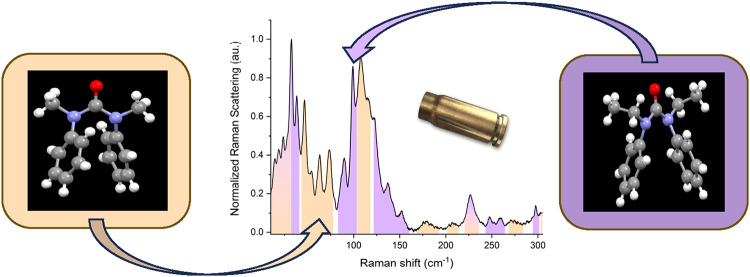

## I Introduction

Trace evidence plays a pivotal role in the field of forensics, particularly when related to gunshot residues from firearms.^1,2^ Gunshot residue (GSR) refers to the conglomerative mix of both organic and inorganic particles expelled from the cartridge of a firearm during discharge.^3^ The composition of GSR can further be broken down into the two subcategories of gunpowder (smokeless powder) and primer.^4^ Since a larger quantity of gunpowder is used in ammunition cartridges relative to primer, the organic gunshot residues (OGSRs) that result from the gunpowder component are convenient targets for GSR detection. Improved methods for detection and quantitation of OGSRs are under constant development to provide accurate analytical profiles of samples arising from firearm usage.

The experimental techniques for OGSR detection have been continually evolving in recent decades. Scanning electron microscopy coupled with energy dispersive X-ray spectrometry (SEM/EDX) is still the standard for detection of inorganic GSRs,^5–8^ while typical methods for OGSR characterization involve mass spectrometry coupled to chromatographic techniques (GC-MS/LC-MS).^9–11^ The focus on GC-MS/LC-MS for OGSRs comes from its demonstrated reliability in obtaining OGSR profiles from different ammunition types, with limits of detection that range in parts-per-billion levels.^12^ There are, however, limitations to these techniques. The samples are destroyed in the process, and the measured data can be complex and difficult to interpret without the assistance of advanced models such as statistical neural networks built through machine learning.^13^ Beyond these common methods, researchers have also investigated OGSRs by a variety of related approaches such as micellar electrokinetic capillary electrophoresis (MECE),^14^ time-of-flight secondary ion mass spectrometry (TOF-SIMS),^15^ portable electrochemical devices,^16^ and desorption electrospray ionization mass spectrometry (DESI-MS).^17,18^

From a vibrational spectroscopy perspective, both Raman and infrared (IR) methods have also been utilized for the identification and characterization of OGSRs.^11,19–21^ Vibrational spectroscopy of OGSRs usually focuses on the measurement of the internal vibrational motions of the molecular species that fall in the energy range of 400–2000 cm^–1^. This is commonly referred to as the “mid-frequency” range and the observed spectral features indicate the presence of particular functional groups, or bonding schemes.^22^ Infrared spectroscopy has been used successfully to characterize OGSRs (i.e., studying blast residues pelletized with KBr), but rigorous statistical analysis was needed due to high spectral complexity.^23,24^ Demonstrations of the forensic value of infrared spectroscopy have also been made through OGSR detection in plastics by acquiring a complete spectrum of nitrocellulose behind a layer of polyethylene.^20^ An ATR-FT-IR approach has also been reported on OGSRs deposited on cloth substrates to detect and differentiate different species, allowing potential correlations between ammunition composition and bullet caliber,^25^ while similar studies have mapped GSRs from different cartridges.^26^

The first published case of using Raman spectroscopy to identify GSRs was in 1998.^27^ The advantages of both Raman and IR are that they are nondestructive and highly selective, where each peak in the spectrum can be attributed to a precise vibrational motion that is specific to each sample. The robustness and reproducibility of using Raman vibrational spectroscopy for OGSR detection has been further established by more recent studies.^28–30^ However, Raman spectroscopy is not a universally applicable solution, and differentiation of OGSRs via Raman spectroscopy has only been demonstrated on a small scale.^31^ A common problem is that typical laser wavelengths in Raman spectroscopy can electronically excite and thereby cause fluorescence in organic molecules, which can severely reduce the signal-to-noise ratio of the Raman signature and distort the spectral baseline. Previous work has demonstrated that surface-enhanced Raman spectroscopy could help address this problem, but noted a new problem with inhomogeneity of the gun powders making it difficult to attribute features to individual components in ammunition.^32^ Generally related to this problem is that samples comprised of OGSR mixtures can be challenging to characterize with vibrational spectroscopy. For example, research has been reported on distinguishing members of a mixture of two nitro containing OGSRs, where it was found that spectral deconvolution was not feasible to assign specific spectral features to each OGSR.^33^

To date, vibrational spectroscopy of OGSRs has focused on relatively high-frequency vibrations.^34^ Motions below ∼300 cm^–1^ have been largely unexplored, though some work has shown data down to 100 cm^–1^.^35,36^ The utilization of low-frequency Raman and terahertz (or far-infrared) spectroscopies should help differentiate between structurally similar OGSRs, both alone and in analyte mixtures. Terahertz time-domain spectroscopy (THz-TDS) has already been proven useful in the detection of illicit drugs and imaging of concealed weapons as it is a penetrating, material-specific, yet nonionizing technique.^37^ Given its capabilities, terahertz spectroscopy has been applied in a wide variety of research areas.^38–40^ For example, it is also able to identify pharmaceutical crystalline polymorphs where the often subtle differences in solid-state structures are made readily apparent through the low-frequency lattice vibrations of the solid samples.^41^ The unique spectral features that accompany this region are specific to different molecules and crystal packing arrangements, allowing easier differentiation of chemically similar samples, as in the case of OGSR mixtures.

Two common OGSRs that are often found in gunpowder as stabilizing additives are the focus of this research: 1,3-diethyl-1,3-diphenylurea (DEDPU) and 1,3-dimethyl-1,3-diphenylurea (DMDPU). DEDPU is often referred to as ethyl centralite and DMDPU as methyl centralite. As shown in Figure 1, these compounds are structurally similar, differing only by methylene (−CH_2_−) groups on the alkyl substituents of the nitrogen atoms. Stabilizers are responsible for neutralizing decomposition products that tend to form in gunpowder formulations. A common example is nitrocellulose, which degrades over time into nitric acid and other oxides of nitrogen, and leads to corrosion and a buildup of strong oxidizers. In turn, this causes a risk of undesirable self-ignition as the degraded gunpowder is extremely temperature and pressure sensitive.^42^ Hence, OGSR stabilizers like DEDPU and DMDPU are often added to avoid these side reactions. DEDPU is one of the most utilized stabilizers, followed by DMDPU.^18,43^

**Figure 1:**
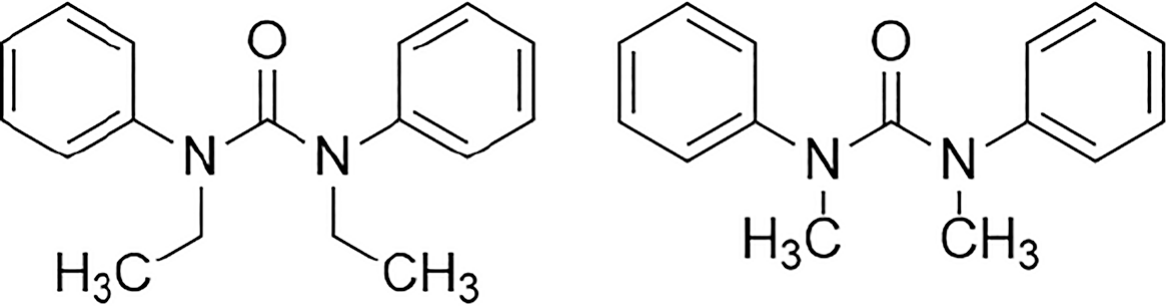
Molecular structures of DEDPU (left) and DMDPU (right).

The work presented here is a detailed investigation using terahertz vibrational spectroscopy (0.30 to 4.00 THz, 10 to 133 cm^–1^) in conjunction with low-frequency Raman vibrational spectroscopy (10 to 350 cm^–1^) to evaluate their capabilities for characterizing both pure and mixed samples of OGSRs. Little OGSR research has been done with either experimental technique, and none in combination. A terahertz spectroscopy study of gunpowder was published in 2013, but no attempts were made to characterize or quantify samples of known OGSRs.^44^ Raman spectroscopy has been performed on DEDPU and its spectrum has been recorded down to 100 cm^–1^.^34^ The final aspect of this comprehensive vibrational study is a computational evaluation of solid-state DEDPU and DMDPU. Solid-state density functional theory (ss-DFT) simulations of the OGSR crystal structures and vibrational motions in the bulk are of great utility. Pairing experiments and simulations enables the assignment of observed spectral features to specific atomic motions in the OGSR crystalline samples. No ss-DFT simulations of these or other OGSRs have been previously reported.

Combining the experimental and computational results facilitates the spectral deconvolution of the THz-TDS and LFRS data sets for both pure and mixed OGSR species. This permits contributions from each molecule to be established and enables the tracking of spectral intensities to obtain limits of detection (LOD) for the OGSR compounds under study. The results of this multifaceted approach demonstrate that the low-frequency Raman and terahertz spectra of OGSRs can successfully serve as characteristic signatures of structurally similar OGSR substances.

## II Materials and Methods

### II.A Materials

DEDPU (C_17_H_20_N_2_O) was purchased from Sigma-Aldrich (99% purity, St. Louis, MO). DMDPU (C_15_H_16_N_2_O) was purchased from Ambeed (95% purity, Arlington Heights, IL). DEDPU was kept at room temperature, but DMDPU was stored at approximately 4 °C, as specified by the vendor. Samples were used as received in their pure forms as well as in binary mixtures created using specific molar ratios (mole fractions) of each OGSR. For mixtures, the OGSRs were individually weighed and then added together in a ball-milling jar and milled to achieve homogeneous mixing.

### II.B Powder X-ray Diffraction (PXRD)

Powder X-ray diffraction measurements were utilized to confirm the bulk crystallinity and purity of each OGSR sample. PXRD measurements were taken using a Bruker (Billerica, MA) D2 Phaser diffractometer (Cu Kα radiation, λ = 1.54060 Å, 2θ = 5–70° with 0.5 s per 0.02° step). Experimental powder patterns were then compared to the predicted patterns based on the structures published in the Cambridge Structural Database (CSD)^45^ as UNUKUK^46^ and CEJDUT^47^ for DEDPU and DMDPU, respectively. PXRD data sets can be found in the .

### II.C Mid-Frequency Raman Spectroscopy (MFRS)

Mid-frequency Raman spectra were acquired at 295 K with a B&W Tek (Newark, NJ) iRaman Plus portable spectrometer using laser excitation centered at λ = 785 nm and a power of 200 mW. A Raman shift spectral range of 100 to 2000 cm^–1^ was used in this work, with a resolution of 4.5 cm^–1^. Samples were finely ground in a ball-mill to increase microcrystal uniformity and homogeneity to minimize intensity discrepancies from crystallite orientations. Each Raman spectrum is a coaddition of 100 spectral acquisitions, each with a 500 ms exposure time. A dark noise spectrum with identical acquisition parameters was collected and subtracted from the final Raman data.

### II.D Low-Frequency Raman Spectroscopy (LFRS)

A Coherent (Santa Clara, CA) THz-Raman system was used to acquire Raman spectra of all samples at 295 K (room temperature), 200 K, and 78 K (liquid nitrogen). The instrument utilizes a laser centered at λ = 784.7 nm for excitation and an Andor Shamrock DR-750 spectrograph with an iDus 416 CCD detector cooled to −70 °C for measurement of the Raman scattered radiation. Samples were prepared in the same way as for the MFRS data collection. They were then placed in a Lake Shore – Janis (Westerville, OH) ST-100 vacuum cryostat-mounted cuvette system with ∼10 mm diameter glass windows. Each sample was held at temperature for a period of 30 min prior to data collection to ensure sample temperature stability. Each Raman spectrum is composed of 225 acquisitions of three-second exposure time accumulations with a laser power of 200 ± 5 mW. Laser power was recorded immediately before each data set was collected. A dark noise spectrum was subtracted from the data to arrive at the final Raman spectrum. All LFRS data have a spectral range of 10 to 350 cm^–1^ and a spectral resolution of 0.6 cm^–1^. Using the Spectragryph software package (version 1.2.16.1, https://www.effemm2.de/index.html), atmospheric interference from N_2_ and O_2_ rotational Raman scattering was identified and removed from the final data.

### II.E Terahertz Time-Domain Spectroscopy (THz-TDS)

Terahertz time-domain spectra were measured using an all-fiber-optic-coupled TeraFlash THz-TDS spectrometer from Toptica Photonics (Munich, Germany). The instrument is based on a λ = 1560 nm femtosecond fiber laser with a 100 μm strip line photoconductive antenna (InGaAs/InP) for terahertz generation. Terahertz detection is done with a photoconductive antenna (InGaAs/InP) with a 25 μm dipole antenna and 10 μm gap. Each sample was ball-milled into fine particles and then mixed with a powdered polytetrafluorethylene (PTFE) matrix to achieve a targeted total mass of 1000 mg with a sample concentration of approximately 3% by mass. The mixture was then remilled to ensure complete mixing. The homogeneous mixture was then pressed into a 13 mm diameter pellet at 2000 psig. Due to losses during the milling and pelleting processes, the final mass of the sample pellets averaged about 900 mg. Each pelleted sample had a minimum thickness of 3 mm to increase the time delay of interfering THz pulse reflections from the pellet surfaces away from the main THz pulse. See Table S30 in the Supporting Information for pellet specifications. This time delay of unwanted reflections prevents signal artifacts in the subsequent Fourier-transformed data sets. An additional pure PTFE pellet was constructed by measuring approximately 900 mg of PTFE and pressed under the same conditions to serve as a matrix reference or blank.

To obtain experimental THz absorption spectra, sample and reference pellets were mounted in the same cryostat used for Raman measurements, but with 3.5 mm thick polymethylpentene (TPX) windows to minimize THz radiation losses and avoid additional pulse reflections in the time-domain data. The THz beam path surrounding the cryostat was purged with dry N_2_ gas to eliminate interference from atmospheric water vapor which absorbs in this region. Terahertz data were collected in a transmission geometry and each time-domain acquisition consisted of a 70 ps time window centered on the main THz pulse, which was then truncated in data processing to 30 ps postpulse to avoid capturing spurious THz pulse reflections. The truncated time-domain terahertz waveform was treated with a Hanning apodization function and zero-padded to total of 17,424 data points prior to Fourier transformation. The final THz absorption spectra are a ratio between the sample and reference pellet data collections and have a spectral bandwidth of 10 to 133 cm^–1^ (0.30 to 4.00 THz, where 1 THz equals 33.35641 cm^–1^) with a spectral resolution of approximately 1.1 cm^–1^. The molar absorption coefficient (ε) is reported in units of M^–1^ cm^–1^ based on a logarithmic scale, with concentration expressed as the molecular molarity of each pelleted OGSR (amount of sample in each pellet).

**Figure 2:**
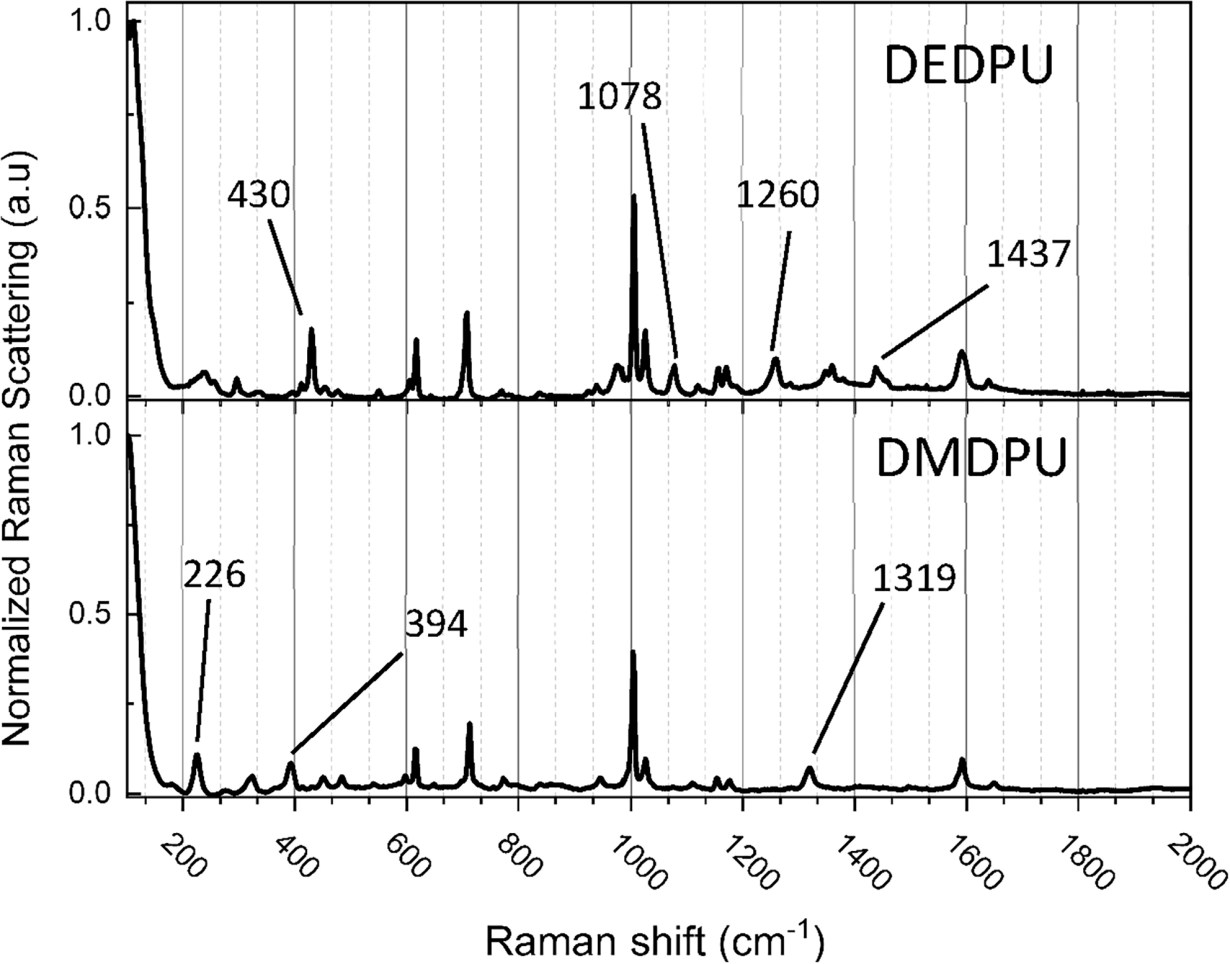
Mid-frequency Raman spectra of DEDPU (top) and DMDPU (bottom) at 295 K. All spectra have been intensity normalized to 1. The most significant compound-specific peaks in each spectrum have been indicated.

**Figure 3:**
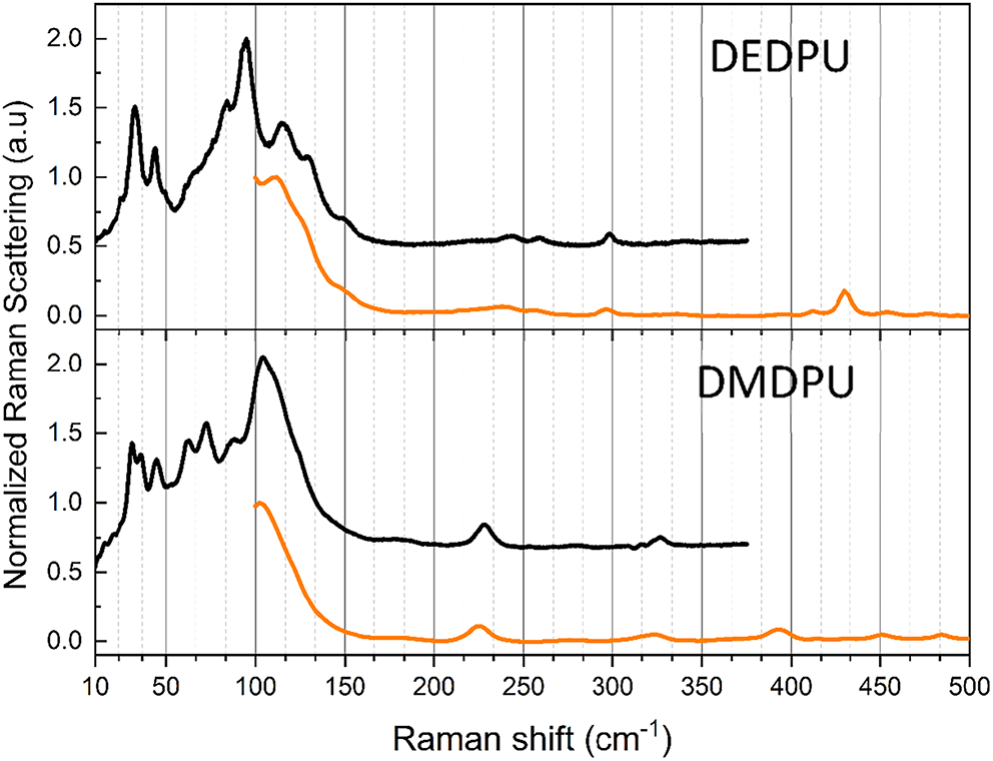
Comparison of 295 K MFRS (orange) and LFRS (black) data for DEDPU (top) and DMDPU (bottom). Spectral intensities have been scaled to achieve matching magnitudes in the overlap region. The LFRS plots have been vertically offset in the figure for visual clarity.

**Figure 4:**
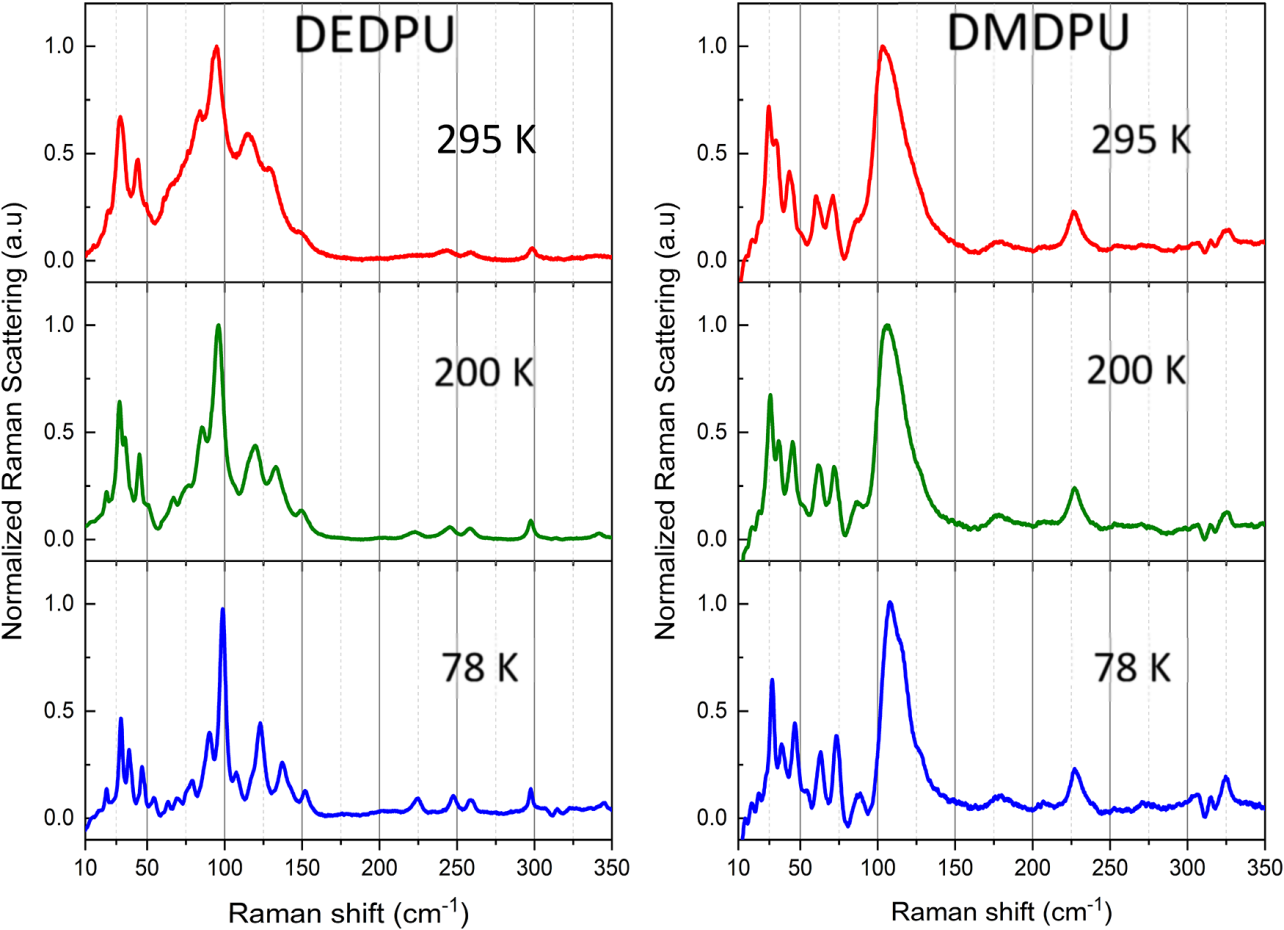
Experimental Raman spectra of DEDPU (left) and DMDPU (right). 295 K spectra are shown in red, 200 K in green, and 78 K in blue. All spectra are intensity normalized to 1.0. The weak feature in the 305–315 cm^–1^ range is a spectral artifact caused by detector noise.

### II.F Density Functional Theory Simulations

The CRYSTAL23 ^48^ software package was used to complete ss-DFT simulations for both OGSRs utilizing periodic boundary conditions to account for the three-dimensional environment of the crystalline materials. Starting structures were obtained from the CSD using the aforementioned reference codes. All simulations were performed within the published space group symmetries of *P*2_1_/*c* for DEDPU and *P*2_1_/*n* for DMDPU. These crystals have asymmetric unit cells consisting of one symmetry-independent molecule (*Z*’ = 1), with complete unit cells having four molecules (*Z* = 4).

The Perdew–Burke–Ernzerhof (PBE)^49^ density functional was used in conjunction with the VTZp^50^ atom-centered basis set. All calculations were augmented by the addition of Grimme’s London dispersion correction (D3) using the Becke-Johnson damping correction (BJ)^51–53^ and the three-body Axilrod–Teller-Muto repulsion contributions (software keyword “ABC”).^54,55^ A pruned integration grid of 99 radial points and 1454 angular points (software keyword “XXLGRID”) was used in the calculations. The *k*-point counts in the irreducible Brillouin zones of the crystals were 39 for both DEDPU and DMDPU, with a shrinking factor of 5 each. The overlap-based truncation criteria for the bielectronic Coulomb exchange integrals were set to 10^–8^, 10^–8^, 10^–8^, 10^–8^, and 10^–16^.

Full structural optimizations (unit cell dimensions and atomic positions) were performed with an energy convergence threshold of Δ*E* < 10^–8^ E_h_. Subsequent vibrational frequency calculations were completed with a stricter energy convergence of Δ*E* < 10^–10^ E_h_. The vibrational analyses of the optimized crystal structures were based on displacements of the atoms of the crystallographic asymmetric unit cell, each displaced twice along the Cartesian axes for determination of the numerical derivatives of the Hessian matrix via the central difference formula. Both Raman and THz intensities were calculated using the coupled-perturbed Hartree–Fock/Kohn–Sham (CPHF/KS)^56–58^ approach. To assist with comparison to experimental measurements, the calculated frequency positions and spectral intensities of the predicted vibrations were convolved with empirical Lorentzian peak shapes. A Lorentzian peak shape was chosen as a simple approximation for visualization only. For the simulated Raman spectra, DMDPU was convolved with a full-width-half-maximum (FWHM) of 6 cm^–1^, and DEDPU used a FWHM of 4 cm^–1^. For the THz spectra, both the DMDPU and DEDPU simulations used FWHM values of 4 cm^–1^.

### II.G Experimental Spectral Feature Analysis

Analysis of spectral features to determine peak positions and widths in the THz and Raman data sets was performed using the “Peak Analyzer” tool in Origin Pro 2024b software (OriginPro *2024b*, OriginLab Corporation, Northampton, MA). The THz-TDS peaks were analyzed over the entire 10 to 133 cm^–1^ spectral range, but in the analysis of the Raman data, spectra were divided into “lower” (10–200 cm^–1^) and “upper” (200–305 cm^–1^) regions for ease of peak fitting and deconvolution. Spectral baselines were corrected by subtracting a fitted polynomial baseline from the original data. Initial peak detection and starting positions were completed using a feature threshold height of 10% of the maximum observed intensity. Subsequent peak fitting was based on a pseudo-Voigt profile (software option: psdvoigt2), using the Levenberg–Marquardt (L–M) algorithm and default software settings for allowable variances. All data fitting converged within 500 iterations with a tolerance of 1 × 10^–6^. To evaluate the results of the experimental spectral feature deconvolution, the number of fitted peaks was compared to the corresponding ss-DFT simulations of pure DMDPU and DEDPU. In both cases, the total number of fitted experimental peaks did not exceed the number of predicted peaks from ss-DFT simulations, confirming reasonable spectral densities were found in the spectral feature analyses.

Signal-to-noise ratio (SNR) values for both the LFRS and THz data sets were calculated using peak heights (signal) determined by the spectral analysis and the standard deviation in the baseline to define the noise level in the spectra. The noise calculations consider only random fluctuations in the spectral baseline and therefore do not factor in broad contributions from fluorescence that may exist in the LFRS spectra.

## III Results

### III.A Experimental Mid-Frequency Raman Data

The mid-frequency Raman spectra of DEDPU and DMDPU are shown in Figure 2 with both spectral baselines corrected for fluorescence and are in agreement with prior work.^35,36^ The uncorrected data can be seen in the . The fluorescence background is weak for DEDPU, but moderate for DMDPU with the uncorrected spectrum having a significantly rising baseline that tends to obscure minor features.

The Raman spectra exhibit numerous peaks in the fingerprint region and the two samples share many similarities. This result is anticipated given the closely related DEDPU and DMDPU chemical structures. A complete list of peak positions in this region is provided in the Supporting Information. While the most prominent features in each spectrum are shared, there are a few distinct peaks that can be used to differentiate the two compounds. These peaks include those at 430, 1078, 1260, and 1437 cm^–1^ in DEDPU and the features at 226, 394, and 1319 cm^–1^ in DMDPU. However, the most representative peaks in the mid-frequency region that are useful for identifying these OGSRs are generally of lower scattering intensity. It is evident that the Raman scattering signal is particularly intense in the sub-150 cm^–1^ region. This observation is worthy of further study using a low-frequency Raman instrument to confirm the strong scattering at low Raman shift and to examine the spectral signatures of the OGSRs in this region. Comparison of the mid-frequency and low-frequency Raman spectra in Figure 3, further highlights the advantages of applying LFRS to the characterization of OGSRs. The enhanced signal-to-noise ratio from the strong Raman scattering evident in the LFRS data provides an opportunity to improve the analytical capabilities of Raman spectroscopy for these types of molecules.

### III.B Experimental Low-Frequency Raman Data

Figure 3 shows a direct comparison of the MFRS data from Figure 2 to LFRS data of the same samples. The LFRS data has been scaled to match the intensities of Figure 2 and confirms for both DEDPU and DMDPU that the Raman scattering signal is approximately three times higher near 100 cm^–1^ when compared to the otherwise most intense feature for these samples at ∼1000 cm^–1^. This enhanced scattering signal at low-frequency improves the signal-to-noise ratios in the OGSR spectra, making it easier to discern spectral differences. The sub −300 cm^–1^ Raman spectra of DEDPU and DMDPU are clearly specific to each compound, with nearly all spectral features serving to definitively identify each OGSR.

Feature resolution in the OGSR spectra is significantly improved with sample cooling. The low-frequency Raman spectra shown in Figure 4 were recorded at 295, 200, and 78 K to demonstrate their temperature dependence. Peak narrowing and shifting is evident at reduced temperature, revealing additional features that are attributable to each OGSR. There is also a two times greater SNR improvement with cooling as the average value increases from SNR ≈ 133 at 295 K to SNR ≈ 256 at 78 K. While sample cooling is beneficial, it is not a requirement, since identifying peaks are evident for all samples at all temperatures.

It is useful to note that peak resolution is realized even with less aggressive cooling to 200 K (−80 °C) which could be achieved using, for example, thermoelectric cooling without consuming a cryogen (e.g., liquid nitrogen for 78 K). As noted for the MFRS data, DMDPU has a noisier baseline compared to DEDPU given that DMDPU is a weaker Raman scatterer and also exhibits fluorescence. The fluorescence emission from DMDPU increases with decreasing temperature which results in a reduced signal-to-noise ratio. This is most likely caused by subtle changes in the packing density (unit cell contraction) of the molecules in the crystalline lattice that promotes electronic absorption of the laser wavelength, thereby increasing fluorescent emission. This phenomenon is illustrated in the unprocessed data figures in the Supporting Information.

Numerical analysis of the spectral features in the 295 K data below 300 cm^–1^, yielded 14 total peaks for DEDPU and 14 total peaks for DMDPU. A list of the room-temperature peak centers is provided in the Supporting Information. The analysis of the 78 K data yielded the results shown in Table[Table tbl1], with 23 total peaks for DEDPU and 24 total peaks for DMDPU, demonstrating the practical value of sample cooling. The improvement of the Raman scattering signal in the low-frequency region along with the unique pattern of spectral features in the sub-300 cm^–1^ range, makes it a powerful tool for distinguishing between structurally similar OGSRs.

**Table 1:** Experimental LFRS and THz-TDS Peak Centers (cm^–1^) with Corresponding Standard Deviations in Parentheses (cm^–1^) for DEDPU and DMDPU at 78 K^a^

LFRS		THz-TDS	
DEDPU	DMDPU	DEDPU	DMDPU
23.9 (0.15)	34.7 (0.04)	51.4 (0.04)	36.9 (0.10)
32.9 (0.04)	42.4 (0.10)	63.0 (0.03)	40.4 (0.02)
38.4 (0.07)	49.3 (0.06)	91.3 (0.02)	52.1 (0.13)
46.8 (0.08)	60.6 (2.08)	98.7 (0.01)	56.7 (0.03)
54.1 (0.20)	62.5 (0.43)	102.9 (0.02)	62.9 (0.05)
63.4 (0.26)	65.3 (0.40)	127.4 (0.01)	77.9 (0.07)
69.4 (0.31)	73.8 (0.22)		89.9 (0.10)
79.7 (0.48)	76.2 (0.08)		101.1 (0.53)
82.9 (0.64)	86.0 (0.30)		104.9 (0.14)
90.4 (0.16)	90.2 (0.28)		114.9 (0.08)
98.8 (0.03)	104.2 (3.48)		132.8 (0.02)
107.5 (0.26)	105.7 (1.58)		
120.4 (1.17)	108.0 (1.15)		
123.3 (0.31)	111.7 (1.00)		
136.6 (2.19)	114.9 (0.75)		
138.7 (3.44)	120.1 (0.74)		
152.1 (0.21)	120.1 (0.52)		
222.9 (0.53)	130.8 (0.40)		
225.4 (0.16)	227.2 (0.27)		
247.7 (0.04)	231.7 (0.91)		
257.8 (0.61)	258.7 (0.58)		
260.2 (0.62)	269.1 (2.04)		
297.5 (0.03)	272.4 (3.22)		
	278.7 (0.75)		

a Raman data analysis yielded fits with *R* ^2^ > 0.97 and average FWHMs of 4.8 cm^–1^. THz-TDS data possessed fits with *R* ^2^ > 0.97 and average FWHMs of 4.5 cm^–1^.

### III.C Experimental THz-TDS

Like the LFRS results, THz-TDS is able to resolve differences between the OGSRs. The THz spectrum of each OGSR is characteristic of the sample under study and can be used for identification and detection of the substance. The THz spectra shown in Figure 5 were recorded at the same temperatures as the LFRS measurements and exhibit the same general peak narrowing and shifting with cooling. Given the absolute spectral intensity units of THz-TDS, the data indicates that DEDPU has approximately 50% greater THz absorption strength than DMDPU, considering the strongest features of each. The THz data benefits significantly from sample cooling, with peak narrowing leading to more readily identifiable features as compared to the room-temperature spectra. The THz spectra also exhibit a gently rising baseline with increasing frequency across the spectral range, likely due to Mie scattering.^59,60^ Strong absorption features near the upper frequency range of the instrument (*vide infra*) appear to also be contributing to the baseline, especially for DMDPU.

**Figure 5:**
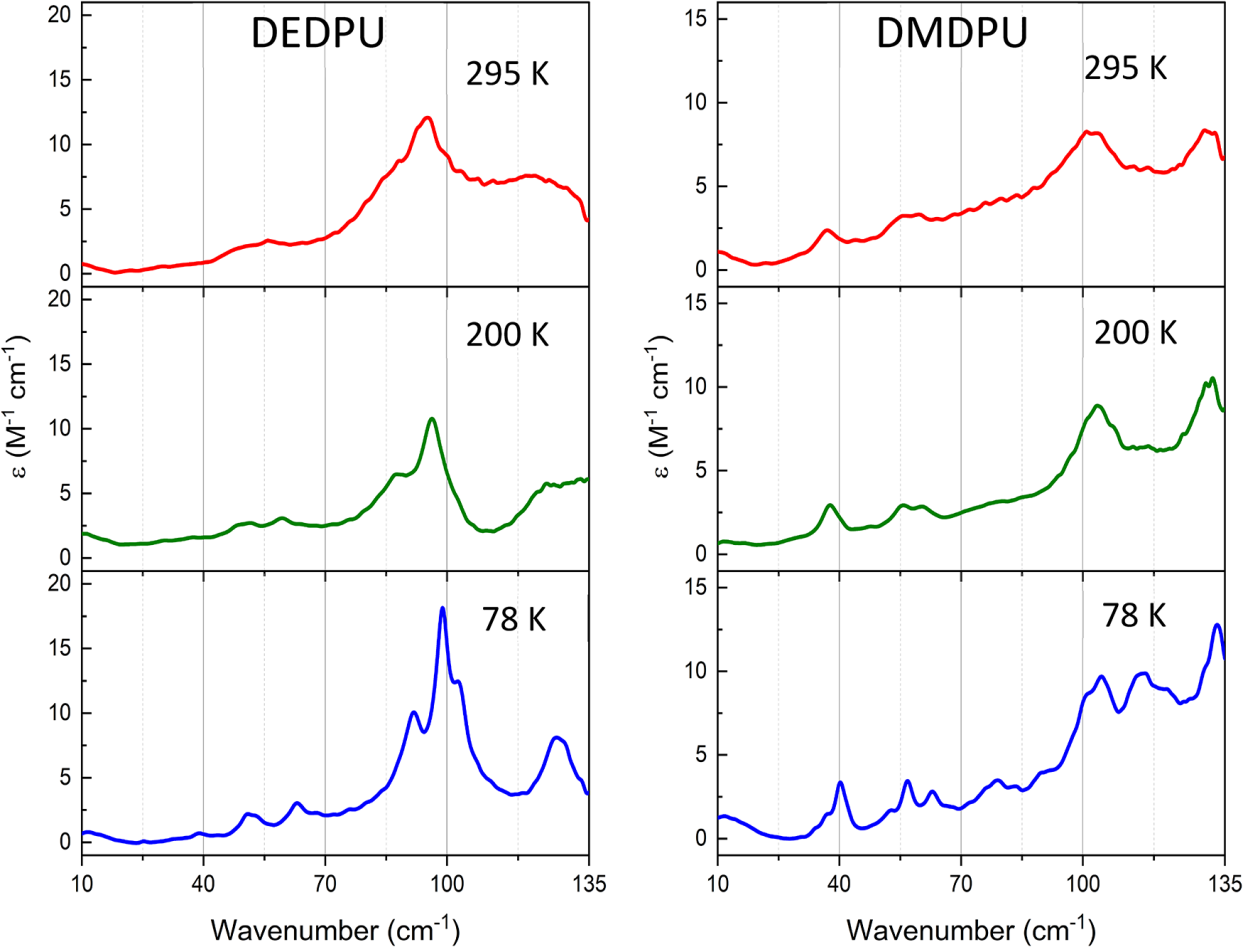
Experimental THz-TDS spectra of DEDPU (left), and DMDPU (right). 295 K spectra are shown in red, 200 K in green, and 78 K in blue.

Compared to the equivalent Raman spectra in the same frequency range, there are noticeably fewer IR-active peaks with strong spectral intensity. The THz-TDS data were subjected to the same numerical analyses as used for the LFRS data. At 295 K, this yielded 4 peaks and 7 peaks for DEDPU and DMDPU, respectively. The 295 K peak centers can be found in the Supporting Information. With cooling to 78 K, 6 peaks were observed for DEDPU and 11 peaks for DMDPU and these are listed in Table 1. Again, with cooling, the SNR increases by five times from a SNR ≈ 4 at 295 K to SNR ≈ 23 at 78 K. These results emphasize the value that sample cooling offers, leading to easier identification and numerical analysis. While not always a factor to consider, sample cooling greatly facilitates comparison of experimental low-frequency vibrational spectroscopy data with ss-DFT spectral simulations that are executed with an implicit temperature of 0 K.

### III.D Solid-State Density Functional Theory Analysis

#### III.D.1 Crystal Structure Optimizations

To assign the observed spectral features in the Raman and THz spectra of the OGSRs, ss-DFT has been utilized to model their crystal structures and vibrational motions. The first step in this process is the accurate simulation of the intramolecular and intermolecular geometries of the samples. Considering first the crystal packing, the simulated unit cell parameters are compared to experimental values in Table S4 of the Supporting Information. Overall, each average percent error for the OGSR lattice dimensions was less than 2.0%. Basis set superposition error (BSSE) in ss-DFT calculations using atom-centered basis sets (as done here) may also contribute to lattice dimension differences, typically manifesting as a unit cell contraction from overestimation of the cohesive energies. The BSSE magnitude was checked for both OGSRs and found to constitute approximately 5% and 6% of the DEDPU and DMDPU cohesion energies, respectively. in the Supporting Information summarizes the BSSE results. Based on the minor role of BSSE in these simulations, no corrections were applied to the presented results.

In terms of internal molecular structure, the root-mean-square deviation (RMSD) values for the intramolecular bond lengths, bond angles, and torsional angles were calculated to evaluate simulation accuracy ( in Supporting Information). The RMSD values were calculated considering all non-hydrogen atoms. Very good agreement was found between experiment and theory with RMSD values for DEDPU being 0.010 Å for bonds, 0.317° for angles, and 3.143° for dihedrals. The values for DMDPU were found to be similar at 0.013 Å, 0.320°, and 3.576°, respectively. The OGSR simulations reveal relatively high torsional RMSD values. While not problematic in this work, it could be attributed to the conformational flexibility and likely high thermal motions of the alkyl groups bonded to the amide nitrogen atoms given the weak interactions governing these groups.

#### III.D.2 Low-Frequency Raman Spectroscopy Simulations

Figure 6 shows a strong correspondence between the simulated Raman spectrum and the 78 K experimental data of each compound. The frequency positions and intensity profiles of the simulations enable the majority of observed spectral features to be assigned to specific atomic motions.

**Figure 6:**
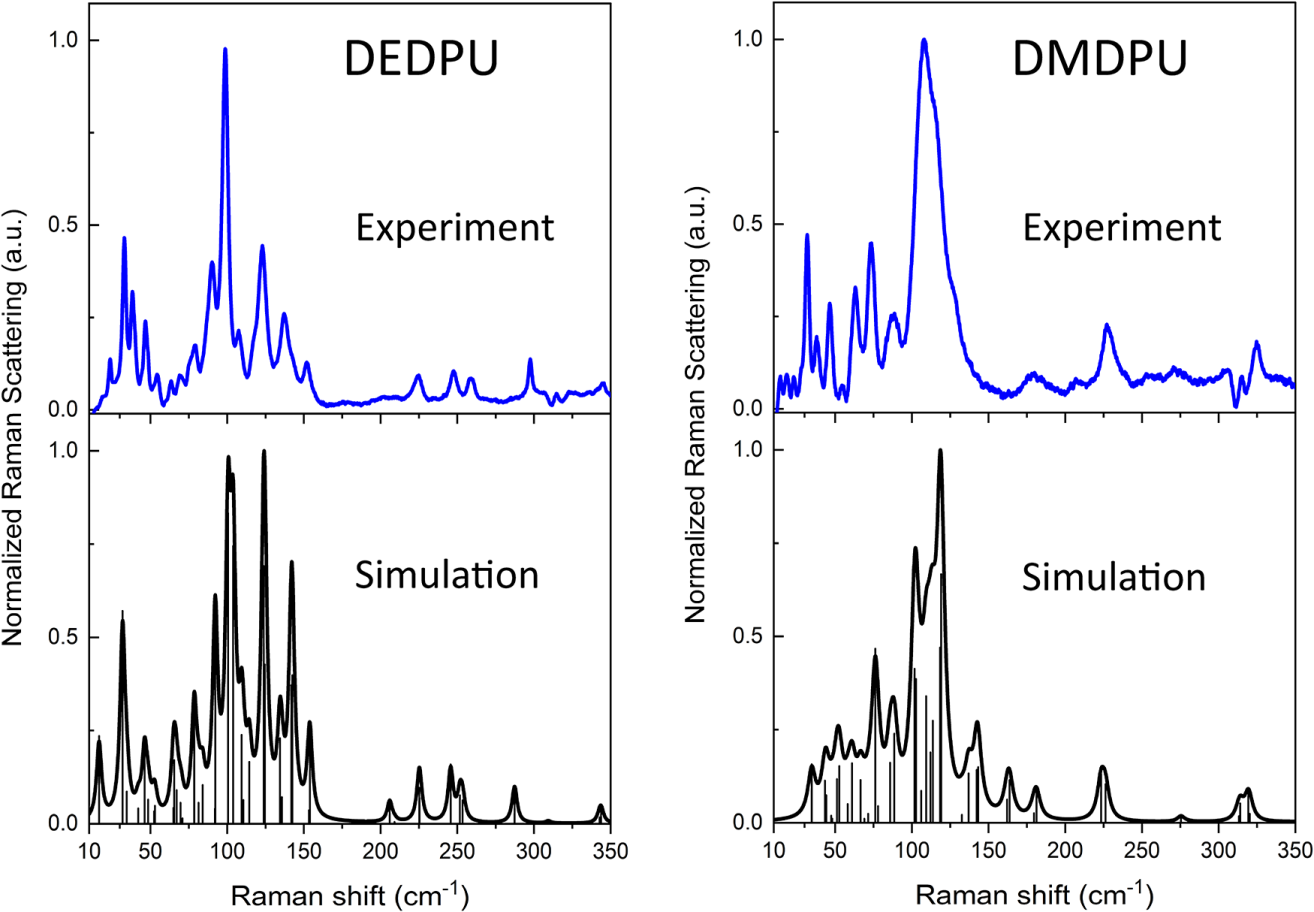
Experimental 78 K Raman spectra (blue) for DEDPU (left) and DMDPU (right) compared to their corresponding ss-DFT simulated Raman spectra (black). Unconvolved simulated data are plotted as impulses. All spectra are intensity normalized to 1.0.

Based on visualization of the calculated normal mode eigenvectors, it is found that both OGSRs display similar vibrational motions in the sub-350 cm^–1^ region. A vibrational type of particular interest is the torsional motion of the phenyl rings as these are common to both OGSRs and expected to be sensitive to crystal packing forces. An inspection of the calculated vibrations reveals that the majority of the modes listed in Table 2 are phenyl torsions and account for the most prominent and intense features in both OGSR spectra. A complete vibrational mode list and select vibration animations (high-intensity modes only) for each OGSR simulation can be found in the Supporting Information. It is important to restate here that while the DEDPU spectrum has an excellent signal-to-noise ratio that eases spectral assignments, DMDPU is less ideal. The experimental DMDPU Raman spectrum has a significant fluorescence background that adds uncertainty to the peak intensities, thereby reducing peak assignment confidence, especially for weaker features.

**Table 2:** Correlation of Experimental Raman and ss-DFT Simulated Vibrational Frequencies (cm^–1^) above a Threshold of 10% of the Maximum Intensity for Experimental DEDPU and DMDPU^a^

DEDPU			DMDPU		
exp. freq	ss-DFT freq.	ss-DFT int.	exp. freq	ss-DFT freq.	ss-DFT int.
23.9	16.52	282.35	34.7	34.71	235.73
32.9	31.82	685.16	42.4	43.37	169.95
				44.23	110.53
38.4	34.54	103.65	49.3	51.19	176.05
46.8	46.17	234.53	60.6	60.97	239.80
54.1	52.92	57.77	62.5	66.57	172.06
63.4	65.40	204.90	65.3	69.06	16.06
69.4	67.00	108.50	73.8	76.12	700.10
79.7	78.55	392.03	76.2	77.95	67.17
82.9	83.99	124.67	86.0	85.83	241.54
90.4	92.23	670.80	90.2	88.53	358.31
98.8	100.64	1000.00	104.2	101.71	619.81
107.5	103.90	893.93	105.7	102.69	578.97
	109.42	286.34		106.11	128.96
120.4	114.41	198.93	108.0	109.32	509.27
123.3	123.79	829.72	111.7	112.13	282.81
	124.54	512.62			
136.6	134.38	275.05	114.9	113.70	410.59
138.7	141.77	446.25	120.1	118.37	705.01
	142.48	477.80			
152.1	153.89	288.15	120.1	119.08	1000.00
222.9	225.24	116.30	130.8	137.02	198.78
225.4	225.38	82.82	227.2	223.37	195.72
247.7	245.69	191.05	231.7	226.22	155.08
257.8	251.75	91.68	258.7	-	-
260.2	253.48	75.52	269.1	-	-
297.5	287.41	101.02	272.4	275.45	29.94
			278.7	-	

a All ss-DFT intensities (arb. units) are normalized to 1000.00 within each simulation. Animations for select vibrational modes are provided in the Supporting Information.

#### III.D.3 Terahertz Time-Domain Spectroscopy Simulations

Figure 7 illustrates the good agreement between the experimental 78 K THz data and the simulated spectra, with all major features accounted for. However, it was found that the simulated spectral intensities were overestimated in the calculations compared to the experimental values. Simulated intensities were scaled down by a factor of 2 for both DEDPU and DMDPU in order to ease comparison with experimental observations. Like the LFRS results, the THz spectra of both OGSRs exhibit numerous peaks in this frequency range that can be assigned to specific motions using the ss-DFT simulated vibrational spectra. Table 3 lists the spectral correlations between experiment and theory in the sub-133 cm^–1^ range, with the full simulation list appearing in the along with animations for select vibrational modes.

**Figure 7:**
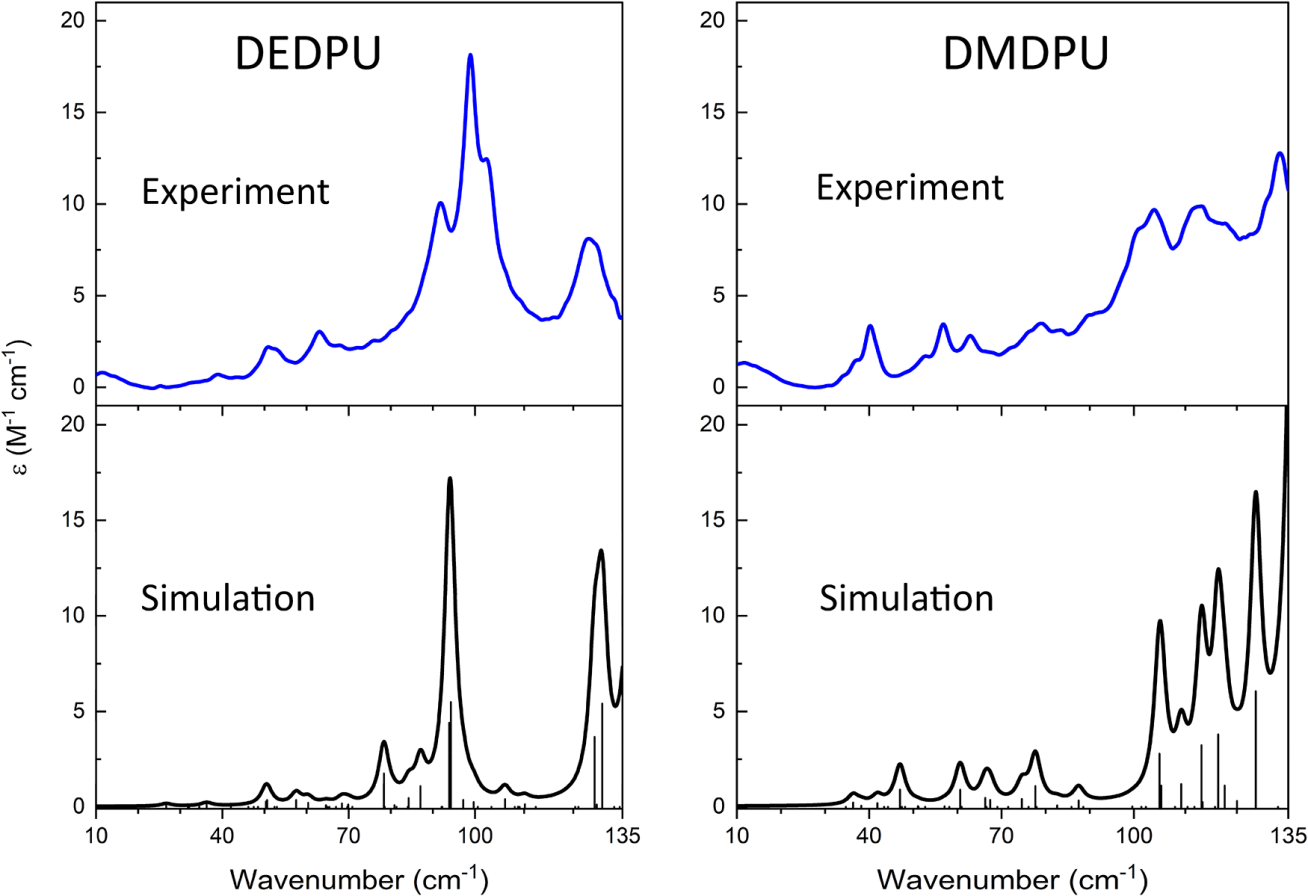
Experimental 78 K THz spectra (blue) for DEDPU (left) and DMDPU (right) compared to their corresponding ss-DFT simulated THz spectra (black). Unconvolved simulated data are plotted as impulses.

**Table 3:** Correlation of Experimental THz-TDS and ss-DFT Simulated Vibrational Frequencies (cm^–1^) above a Threshold of 10% of the Maximum Intensity for Experimental DEDPU and DMDPU^a^

DEDPU			DMDPU		
exp. freq	ss-DFT freq	ss-DFT int.	exp. freq	ss-DFT freq	ss-DFT int.
36.7	36.27	0.08	36.7	36.35	0.17
50.0	50.61	0.27	40.0	41.84	0.14
63.4	57.52	0.27	53.4	46.98	0.68
90.1	93.85	3.30	56.7	60.61	0.67
	94.22	4.12			
100.1	99.68	0.18	63.4	66.32	0.35
103.4	107.13	0.30	76.7	77.67	0.81
126.8	128.37	2.75	90.1	87.48	0.25
	130.18	4.06			
			103.4	105.82	2.09
			106.7	106.20	0.83
			113.4	115.31	2.43
			120.1	119.11	2.85
				120.57	0.83
			133.4	127.63	4.54

a Listed ss-DFT intensities (km/mol) are per molecule (rather than per unit cell) and have not been scaled.

Considering vibrational mode character, there are some similarities between the motions observed in the THz-TDS spectra (IR-active) and those in the LFRS data, with ring–chain torsions found in both. An apparent difference is that the alkyl groups contribute more to the character of the THz-active vibrations compared to the Raman-active motions (see animations in Supporting Information). It is also noteworthy that the experimental peak analyses and ss-DFT simulations show that the LFRS spectra in the region below 133 cm^–1^ possess a higher quantity of observable peaks than the THz-TDS data. The number of observable spectral features has a direct influence on the ability of the different spectroscopies to detect OGSRs.

## IV Mixture Analysis Using LFRS

A direct comparison of the number of resolved peaks, their positions, and the signal-to-noise ratios in the THz-TDS and LFRS data sets indicates that LFRS is the superior low-frequency vibrational spectroscopy technique for detecting and quantifying DEDPU and DMDPU in solid samples. On average, the 78 K LFRS data possesses a SNR ≈ 256 compared to the 78 K THz data with a SNR ≈ 23. The difference indicates that for these particular samples, there is a significantly greater SNR in the LFRS spectra. While LFRS may be the technique of choice for these particular OGSRs, it is not necessarily always higher performing, and the choice of spectroscopy will be dictated by the specific spectral profiles of the OGSRs of interest. As shown in Figures 4 and 5, greater peak resolution is achieved at reduced sample temperatures and therefore further analyses will focus on the 78 K experimental Raman data.

Binary mixtures of DEDPU and DMDPU were investigated to explore the feasibility of discerning the presence of each in a given sample. Raman spectra were measured of several DEDPU:DMDPU mixtures which are referred to in this work according to the mole fraction of DEDPU in the mixture and include 0.10, 0.25, 0.50, 0.75, and 0.90. The DEDPU:DMDPU mixture spectra were then analyzed using the LFRS spectral peak information derived from the numerical peak-shape analysis of the pure samples. Each mixture was treated as a linear combination of the two OGSRs when subjected to analysis. Confirmation of the noninteracting nature of the two OGSRs in the mixtures was made through PXRD measurements of the equimolar mixture revealing no new features (see Supporting Information). Peak fitting of the mixture spectra was initiated using the fitted peak values from the pure OGSRs as starting parameters for the peak-shape analysis and also to set the maximum number of total peaks possible (similar to how the simulations were used for the pure samples). Other than preseeding the starting values, the same numerical peak-shape analysis was applied to the mixtures as in the pure samples. The analysis of the LFRS data was split into two regions (upper and lower) to improve numerical convergence of the peak fitting routine by decreasing the number of peaks being fit simultaneously and reducing the weighting of the intense spectral features below approximately 100 cm^–1^. In the lower 10–200 cm^–1^ region, a maximum of 35 peaks were included in the peak analysis, while 12 were used in the upper 200–305 cm^–1^ portion.

The upper region of the LFRS data considered in this study is potentially of value, but has limitations. Unlike the more densely populated 10–200 cm^–1^ region, the peaks present here generally offer better baseline resolution and are therefore easier to characterize (Figure 8). The only exceptions are the peaks from DEDPU and DMDPU that overlap between 200 and 235 cm^–1^. However, looking at the data more closely, it becomes apparent that the spectral features in this region are dominated by DEDPU, thereby making this region impractical for analytical analysis of DEDPU:DMDPU mixtures. The Raman scattering signal of both OGSRs is relatively weak between 200 and 305 cm^–1^, but DMDPU is significantly weaker, so much so that only a single peak at 278.7 cm^–1^ is attributable to it. The signal-to-noise ratio for this particular peak is insufficient for reliable quantification of DMDPU (less than 1). Therefore, analytical analysis will focus on more prominent and distinguishable peaks in the 10–200 cm^–1^ region that are useful signifiers for both DEDPU and DMDPU content.

**Figure 8:**
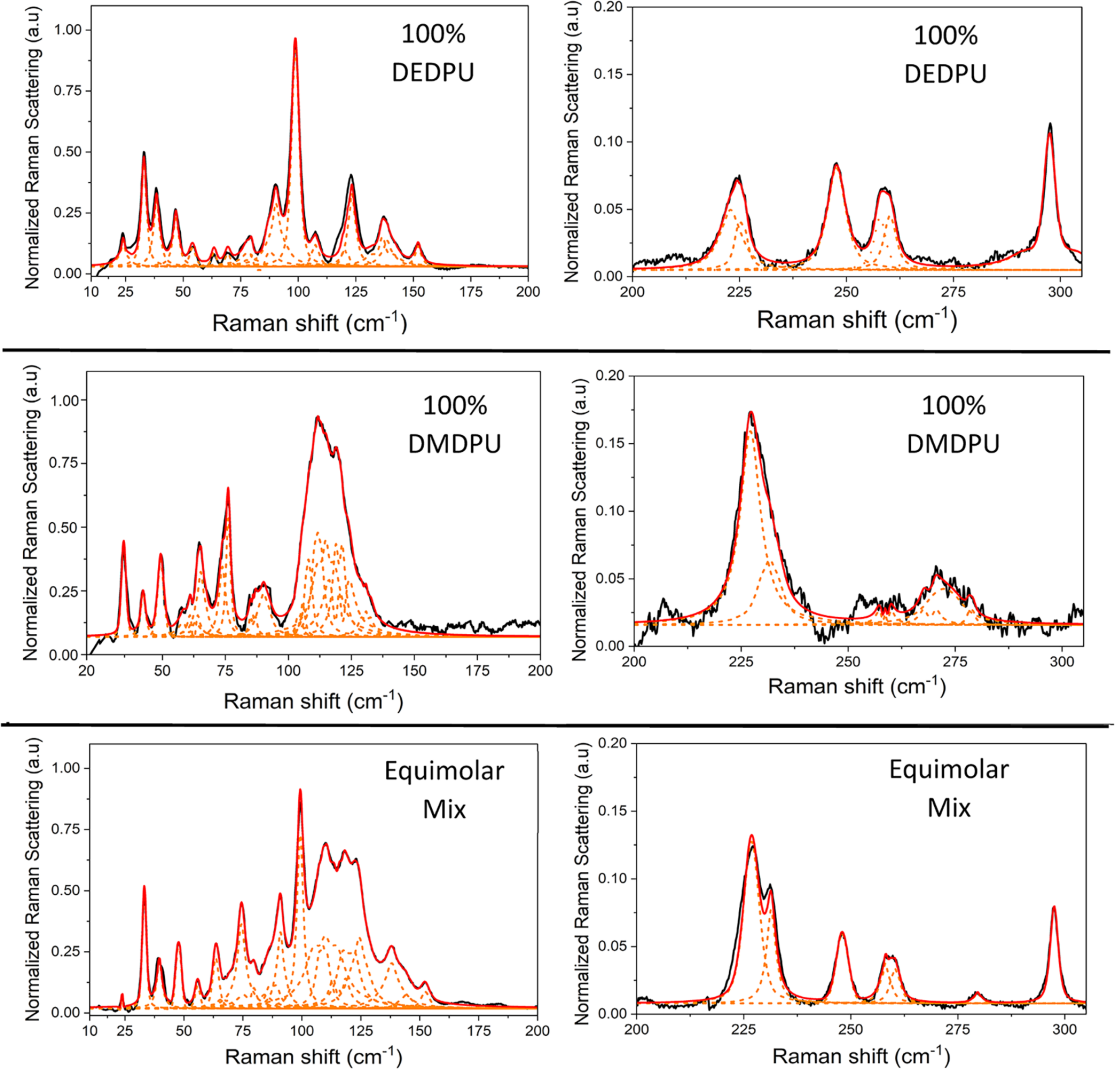
Comparison between peak-shape analyses for pure DEDPU (top), pure DMDPU (middle), and equimolar (mole fraction = 0.5) mix (bottom) at 78 K in the 10–200 cm^–1^ region (left) and the 200–305 cm^–1^ region (right). The experimental trace is shown in black, individual fitted peaks shown in orange, and aggregate fitted trace shown in red. All spectra have been intensity normalized to 1.00.

Figure 8 serves to illustrate how the equimolar (mole fraction = 0.50) binary mixture can be treated as a linear combination of the two individual OGSRs. Given the increased density of features, there are clearly more peaks overlapping in the equimolar mixture and thus some features from the pure samples are obscured. Only 20 peaks were identified with high confidence in the numerical peak-shape analysis. Table 4 provides the results of the data fitting and a majority of the listed peak centers can readily be correlated within the standard deviations to the values provided in Table 1 for the pure OGSR samples.

**Table 4:** Results of spectral peak analysis of the 78 K low-frequency Raman data for an equimolar binary mixture of DEDPU and DMDPU. See Table 2 for peak correlation with pure OGSR samples

peak centers and std. dev. (cm^–1^)	attributable OGSR
23.8 (0.05)	DEDPU
33.2 (0.01)	DEDPU
39.6 (0.04)	DEDPU
47.7 (0.02)	DEDPU
55.8 (0.07)	DEDPU
63.5 (0.14)	DEDPU
67.2 (0.43)	DMDPU
74.4 (0.05)	DMDPU
79.7 (0.14)	DEDPU
87.8 (0.56)	DMDPU
90.9 (0.09)	both
99.3 (0.51)	DEDPU
109.0 (0.25)	DMDPU
110.0 (0.98)	DMDPU
115.9 (0.26)	DMDPU
119.8 (0.96)	both
124.4 (0.59)	DEDPU
138.1 (0.16)	DEDPU
144.1 (0.51)	DEDPU
152.3 (0.14)	DEDPU
226.8 (0.06)	both
231.4 (0.10)	DMDPU
248.0 (0.07)	DEDPU
258.1 (0.72)	DEDPU
260.4 (0.67)	DEDPU
279.6 (0.57)	DMDPU
297.5 (0.06)	DEDPU

The same analysis methodology was applied to the other four mole ratio mixtures, and the experimental spectra and peak analyses are available in the Supporting Information. The most important aspect of the overall mixture analysis is that there are small quantities of peaks that are exclusively attributable to each OGSR. The intensities of these peaks track proportionally across all mixture compositions and can be used for the unambiguous identification and quantification of DEDPU and DMDPU in the mixtures. The most distinctive peaks in the equimolar ratio are found at 99.3 cm^–1^ (from DEDPU) and 110.0 cm^–1^ (from DMDPU) which correspond to the peaks at 98.8 and 111.7 cm^–1^ in the pure samples, respectively (within the standard deviation of the fits). The intensity variations of these two peaks with changes in the mixture ratios are shown in Figure 9. It may be possible to distinguish additional spectral features, but these specific peaks were selected due to their intensities and acceptable overlap with neighboring peaks. In addition, they are even visible in the room-temperature data, albeit with lower signal-to-noise ratios. The limit of detection (LOD) can be determined for each OGSR in the binary mixtures based on the measured peak intensities and their uncertainties. The LOD for the peak at 98.8 cm^–1^ is a molar ratio of 0.13, or 13% DEDPU. Likewise, the peak at 111.7 cm^–1^ has a LOD of 0.21 or 21% DMDPU. The higher LOD value for the peak at 111.7 cm^–1^ can be attributed to the fact that DMDPU is the weaker Raman scatterer of the pair.

**Figure 9:**
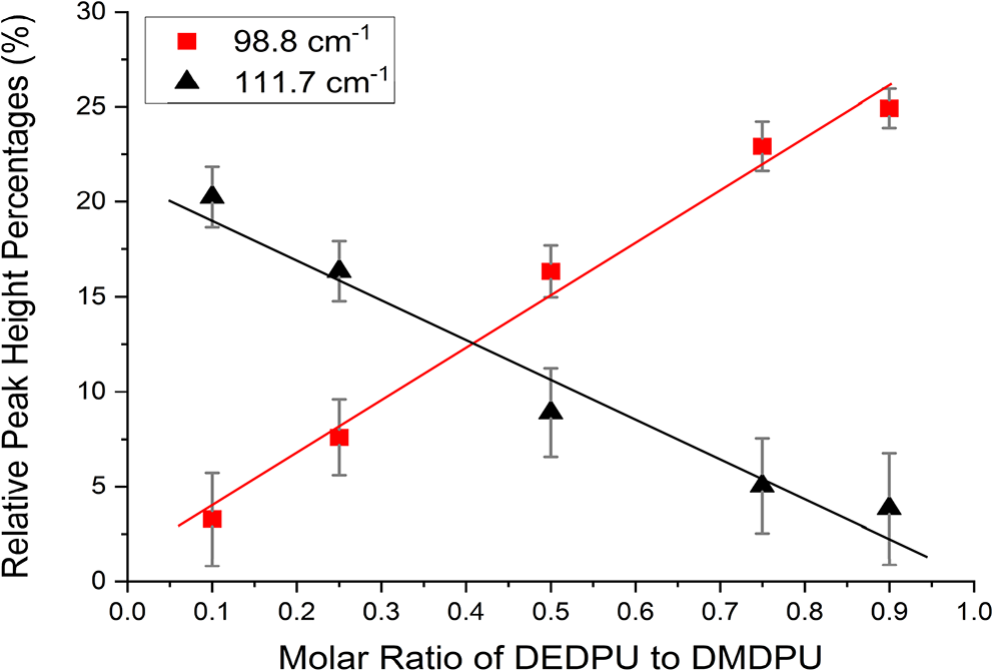
Linear regression best-fit line analysis for the most prominent peaks of DEDPU and DMDPU across all binary mole fractions. The fit illustrates the relative peak height percentages across each mixture composition, with vertical error bars indicating the peak height uncertainty. The red data correspond to the peak at 98.8 cm^–1^ from pure DEDPU, while the black data correspond to the peak at 111.7 cm^–1^ from pure DMDPU.

## V Conclusions

This study establishes low-frequency Raman spectroscopy and terahertz time-domain spectroscopy as effective and complementary techniques for revealing the characteristic spectral signatures of organic gunshot residues. The spectral patterns in the Raman and THz spectra of DEPDU and DMDPU provide a means for not only detection of the pure substances, but also for evaluating mixtures of OGSRs. The measured spectra demonstrate that even moderate sample cooling can significantly narrow the peak widths, thereby offering more opportunities to enable and improve OGSR detection. For DEDPU and DMDPU, LFRS was found to be the superior choice for analytical applications. This is partly due to the greatly enhanced Raman scattering signal for these OGSRs at low frequency as compared to the common fingerprint region. The Raman peaks at 98.8 cm^–1^ for pure DEDPU and 111.7 cm^–1^ for pure DMDPU are good markers of these compounds in mixtures and valuable for quantifying content.

The use of quantum mechanical simulations with periodic boundary conditions are of key importance in understanding the low-frequency vibrations of OGSR solids. Solid-state DFT yielded high-quality crystal structures and permitted assignment of the measured spectral peaks to specific large-amplitude vibrations of the molecules within each crystalline lattice. Lattice vibrations with intermolecular character are often expected to dominate vibrational spectra at low frequencies, but intramolecular phenyl torsions are the primary contributors to the LFRS and THz-TDS profiles of DEDPU and DMDPU. This finding shows that such rigorous simulations are needed for the rational explanation and application of low-frequency spectral data of crystalline solids as the chemical origins of these spectra cannot be assumed. Overall, these insights demonstrate that small structural differences between OGSRs translate into large lattice vibrational frequency differences, providing a new foundation for molecular discrimination.

## Supplementary Material







## Notes

DEDPU animations (ZIP)
DMDPU animations (ZIP)
Full list of fitted Raman and THz peak centers for DEDPU and DMDPU at 295 and 78 K; PXRD patterns for DMDPU and DEDPU; crystallographic unit cell images for each OGSR; lists of all calculated vibrational frequencies and intensities (IR and Raman) for each OGSR; animations of lattice vibrations of DEDPU and DMDPU down the a-axis with extended unit cell for visualization (*P*1 representation); mid-frequency IR spectra for each OGSR; uncorrected mid-frequency and low-frequency Raman data for OGSRs; all peak fitted low-frequency Raman mixture plots; tables for each fitted mixture with peak centers, standard deviations, Gaussian and Lorentzian contributions, and the Gaussian:Lorentzian ratio; linear regression plots for 3 peaks used in LOD calculations in the 200–305 cm^–1^ region; exact THz pellet specifications; SNR values for DEDPU and DMDPU at 295 and 78 K (PDF)

## References

[1] Aleksandar, I. Is there a way to precisely identify that the suspect fired from the firearm?. In Forensic Science International; Elsevier Ireland Ltd Elsevier House: Brookvale Plaza, East Park Shannon, Co, 2003; pp 158–159.

[2] Shrivastava P., Jain V. K., Nagpal S. (2021). Gunshot residue
detection technologies—a
review. Egypt. J. Forensic Sci..

[3] Goudsmits E., Sharples G. P., Birkett J. W. (2015). Recent
trends in organic gunshot
residue analysis. TrAC, Trends Anal. Chem..

[4] Hagel Rainer, R. K. Use of Zinc Peroxide as Oxidant for Explosives and Pyrotechnical Mixtures. US4363679A,, 1982.

[5] Brożek-Mucha Z. (2014). Scanning Electron
Microscopy and X-Ray Microanalysis for Chemical and Morphological
Characterisation of the Inorganic Component of Gunshot Residue: Selected
Problems. BioMed. Res. Int..

[6] Charles, S. End user commentary on advances in analysis of gunshot residue. In Emerging Technologies for the Analysis of Forensic Traces; Springer, 2019; pp 203–206 10.1007/978-3-030-20542-3_14.

[7] Romolo, F. S. Advances in analysis of gunshot residue. In Emerging Technologies for the Analysis of Forensic Traces; Springer, 2019; pp 183–202 10.1007/978-3-030-20542-3_13.

[8] Bonnar C., Moule E. C., Lucas N., Seyfang K. E., Dunsmore R. P., Popelka-Filcoff R. S., Redman K., Paul Kirkbride K. (2020). Tandem detection
of organic and inorganic gunshot residues using LC–MS and SEM-EDS. Forensic Sci. Int..

[9] Goudsmits E., Sharples G. P., Birkett J. W. (2016). Preliminary
classification of characteristic
organic gunshot residue compounds. Sci. Justice.

[10] Feeney W., Vander Pyl C., Bell S., Trejos T. (2020). Trends in composition,
collection, persistence, and analysis of IGSR and OGSR: A review. Forensic Chem..

[11] Charles S., Geusens N., Nys B. (2023). Interpol Review of
Gunshot Residue
2019 to 2021. Forensic Sci. Int.:Synergy.

[12] Vander
Pyl C., Feeney W., Arroyo L., Trejos T. (2023). Capabilities and limitations
of GC–MS and LC-MS/MS for trace detection of organic gunshot
residues from skin specimens. Forensic Chem..

[13] Feeney W., Menking-Hoggatt K., Vander Pyl C., Ott C. E., Bell S., Arroyo L., Trejos T. (2021). Detection of organic and inorganic
gunshot residues from hands using complexing agents and LC-MS/MS. Anal. Methods.

[14] Northrop D. (2001). Gunshot Residue
Analysis by Micellar Electrokinetic Capillary Electrophoresis: Assessment
for Application to Casework. Part I. J. Forensic
Sci..

[15] Mahoney C.
M., Gillen G., Fahey A. J. (2006). Characterization of gunpowder samples
using time-of-flight secondary ion mass spectrometry (TOF-SIMS). Forensic Sci. Int..

[16] Dalzell K. A., Ott C. E., Trejos T., Arroyo L. E. (2022). Comparison of portable
and benchtop electrochemical instruments for detection of inorganic
and organic gunshot residues in authentic shooter samples. J. Forensic Sci..

[17] Morelato M., Beavis A., Ogle A., Doble P., Kirkbride P., Roux C. (2012). Screening of gunshot
residues using desorption electrospray ionisation–mass
spectrometry (DESI–MS). Forensic Sci.
Int..

[18] Taudte R. V., Roux C., Bishop D., Blanes L., Doble P., Beavis A. (2015). Development of a UHPLC
method for the Detection of
Organic Gunshot Residues using Artificial Neural Networks. Anal. Methods.

[19] Krishna S., Ahuja P. (2023). A chronological study of gunshot residue (GSR) detection techniques:
a narrative review. Egypt. J. Forensic Sci..

[20] López-López M., García-Ruiz C. (2014). Infrared and
Raman spectroscopy techniques applied
to identification of explosives. TrAC, Trends
Anal. Chem..

[21] Bueno J., Lednev I. (2013). Advanced statistical analysis and discrimination of
gunshot residue implementing combined Raman and FT-IR data. Anal. Methods.

[22] Pasquarella C., Bertoni S., Passerini N., Boyd B. J., Be̅rziņš K. (2023). Comparison
of Low-, Mid-, and High-Frequency Raman Spectroscopy for an In Situ
Kinetic Analysis of Lipid Polymorphic Transformations. Cryst. Growth Des..

[23] Banas K., Banas A., Moser H., Bahou M., Li W., Yang P., Cholewa M., Lim S. (2010). Multivariate analysis
techniques in the forensics investigation of the postblast residues
by means of fourier transform-infrared spectroscopy. Anal. Chem..

[24] Sharma S., Lahiri S. (2009). A preliminary investigation
into the use of FTIR microscopy
as a probe for the identification of bullet entrance holes and the
distance of firing. Sci. Justice.

[25] Bueno J., Sikirzhytski V., Lednev I. K. (2013). Attenuated Total Reflectance-FT-IR
Spectroscopy for Gunshot Residue Analysis: Potential for Ammunition
Determination. Anal. Chem..

[26] López-López M., Fernandez
de la Ossa M. A., García-Ruiz C. (2015). Fast Analysis
of Complete Macroscopic Gunshot Residues on Substrates Using Raman
Imaging. Appl. Spectrosc..

[27] Stich S., Bard D., Gros L., Wenz H. W., Yarwood J., Williams K. (1998). Raman Microscopic Identification of Gunshot Residues. J. Raman Spectrosc..

[28] López-López M., Delgado J. J., García-Ruiz C. (2012). Ammunition Identification by Means
of the Organic Analysis of Gunshot Residues Using Raman Spectroscopy. Anal. Chem..

[29] Badawi H. M., Förner W. (2012). Analysis of
the infrared and Raman spectra of the symmetrically
substituted 1,3-diphenylurea and 1,3-diphenylacetone (dibenzyl ketone). Spectrochim. Acta, Part A.

[30] Bueno J., Halámková L., Rzhevskii A., Lednev I. (2018). Raman microspectroscopic mapping
as a tool for detection
of gunshot residue on adhesive tape. Anal. Bioanal.
Chem..

[31] Khandasammy S. R., Bartlett N., Halámková L., Lednev I. (2023). Hierarchical
Modelling of Raman Spectroscopic Data Demonstrates the Potential for
Manufacturer and Caliber Differentiation of Smokeless Powders. Chemosensors.

[32] López-López M., Merk V., García-Ruiz C., Kneipp J. (2016). Surface-enhanced Raman
spectroscopy for the analysis of smokeless gunpowders and macroscopic
gunshot residues. Anal. Bioanal. Chem..

[33] Bueno J., Sikirzhytski V., Lednev I. K. (2012). Raman Spectroscopic Analysis of Gunshot
Residue Offering Great Potential for Caliber Differentiation. Anal. Chem..

[34] Mistek E., Fikiet M. A., Khandasammy S. R., Lednev I. K. (2019). Toward Locard’s
Exchange Principle: Recent Developments in Forensic Trace Evidence
Analysis. Anal. Chem..

[35] Thayer E., Turner W., Blama S., Devadas M. S., Hondrogiannis E. M. (2019). Signal
detection limit of a portable Raman spectrometer for the SERS detection
of gunshot residue. MRS Commun..

[36] Estevanes J., Monjardez G. (2024). Detection
of organic explosive residues from outdoor
detonations using confocal Raman microscopy. Forensic Sci. Int..

[37] Davies A. G., Burnett A. D., Fan W., Linfield E. H., Cunningham J. E. (2008). Terahertz
spectroscopy of explosives and drugs. Mater.
Today.

[38] Menchu N., Chaudhary A. K. (2023). Cost-Effective Assessment of Water
Content in Indian
Almond (Catappa) of Various Colored Leaves Using Continuous-Wave Terahertz
Spectroscopy and Principal Component Analysis. J. Opt. Photonics Res..

[39] Huang H., Liu Z., Ruggiero M. T., Zheng Z., Qiu K., Li S., Zhang Z., Zhang Z. (2025). Terahertz Geoscience:
THz Time-Domain
Spectroscopy for Mineral Materials. Cryst. Growth
Des..

[40] Sumesh M. A., Karanth S., Sriram K. (2024). A Compact THz Photometer for Solar
Flare Burst Studies from Space. J. Opt. Photonics
Res..

[41] King M. D., Buchanan W. D., Korter T. M. (2011). Identification
and Quantification
of Polymorphism in the Pharmaceutical Compound Diclofenac Acid by
Terahertz Spectroscopy and Solid-State Density Functional Theory. Anal. Chem..

[42] Maitre M., Horder M., Kirkbride K. P., Gassner A.-L., Weyermann C., Roux C., Beavis A. (2018). A forensic
investigation on the persistence
of organic gunshot residues. Forensic Sci. Int..

[43] Serol M. A.-O., Ahmad S. A.-O., Quintas A. A.-O., Família C. A.-O. (2023). Chemical
Analysis of Gunpowder and Gunshot Residues. Molecules.

[44] Tomáš, G. ; Vojtěch, K. Terahertz time-domain spectroscopy for distinguishing different kinds of gunpowder Proc. SPIE 2013 89000H.

[45] Groom C. R., Bruno I. J., Lightfoot M. P., Ward S. C. (2016). The Cambridge Structural
Database. Acta Crystallogr., Sect. B.

[46] Betz R., Gerber T., Schalekamp H. (2011). 1,3-Diethyl-1,3-diphenylurea. Acta Crystallogr., Sect. E.

[47] Yamasaki R., Iida M., Ito A., Fukuda K., Tanatani A., Kagechika H., Masu H., Okamoto I. (2017). Crystal Engineering
of N,N′-Diphenylurea Compounds Featuring Phenyl–Perfluorophenyl
Interaction. Cryst. Growth Des..

[48] Erba A., Desmarais J. K., Casassa S., Civalleri B., Donà L., Bush I. J., Searle B., Maschio L., Edith-Daga L., Cossard A., Ribaldone C., Ascrizzi E., Marana N. L., Flament J.-P., Kirtman B. (2023). CRYSTAL23:
A Program for Computational Solid State Physics and Chemistry. J. Chem. Theory Comput..

[49] Perdew J. P., Burke K., Ernzerhof M. (1996). Generalized
Gradient Approximation
Made Simple. Phys. Rev. Lett..

[50] Schäfer A., Horn H., Ahlrichs R. (1992). Fully optimized contracted Gaussian
basis sets for atoms Li to Kr. J. Chem. Phys..

[51] Grimme S., Ehrlich S., Goerigk L. (2011). Effect of
the damping function in
dispersion corrected density functional theory. J. Comput. Chem..

[52] Grimme S., Antony J., Ehrlich S., Krieg H. (2010). A consistent and accurate
ab initio parametrization of density functional dispersion correction
(DFT-D) for the 94 elements H-Pu. J. Chem. Phys..

[53] Grimme S., Hansen A., Brandenburg J. G., Bannwarth C. (2016). Dispersion-Corrected
Mean-Field Electronic Structure Methods. Chem.
Rev..

[54] Axilrod B. M., Teller E. (1943). Interaction of the van der Waals
Type Between Three
Atoms. J. Chem. Phys..

[55] Donà L., Brandenburg J. G., Bush I. J., Civalleri B. (2020). Cost-effective
composite methods for large-scale solid-state calculations. Faraday Discuss..

[56] Ferrero M., Rérat M., Kirtman B., Dovesi R. (2009). Calculation of first
and second static hyperpolarizabilities of one- to three-dimensional
periodic compounds. Implementation in the CRYSTAL code. J. Chem. Phys..

[57] Ferrero M., Rérat M., Orlando R., Dovesi R. (2008). Coupled perturbed Hartree-Fock
for periodic systems: The role of symmetry and related computational
aspects. J. Chem. Phys..

[58] Ferrero M., Rérat M., Orlando R., Dovesi R. (2008). The calculation of
static polarizabilities of 1–3D periodic compounds. the implementation
in the crystal code. J. Comput. Chem..

[59] Garet F., Hofman M., Meilhan J., Simoens F., Coutaz J. L. (2014). Evidence
of Mie scattering at terahertz frequencies in powder materials. Appl. Phys. Lett..

[60] Franz M., Fischer B. M., Walther M. (2008). The Christiansen effect in terahertz
time-domain spectra of coarse-grained powders. Appl. Phys. Lett..

