# Supplementary figures and images for: Characterizing Organic Gunshot Residues with Low-Frequency Raman and Terahertz Vibrational Spectroscopies
Source: ACS Omega. 2026 Jan 23;11(5):8209–21. doi: 10.1021/acsomega.5c10754 (PMC12903151; doi:10.1021/acsomega.5c10754)

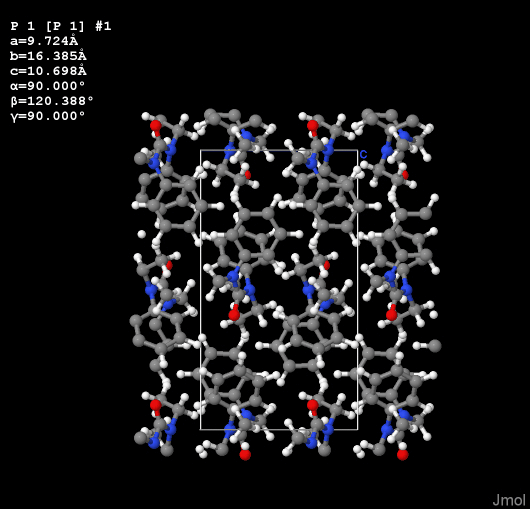

Supplement: Supplementary file 1 [file ao5c10754_si_001.zip › Select_DEDPU_Animations/Raman/100.64 - Mode 41/100-Mode41.gif]

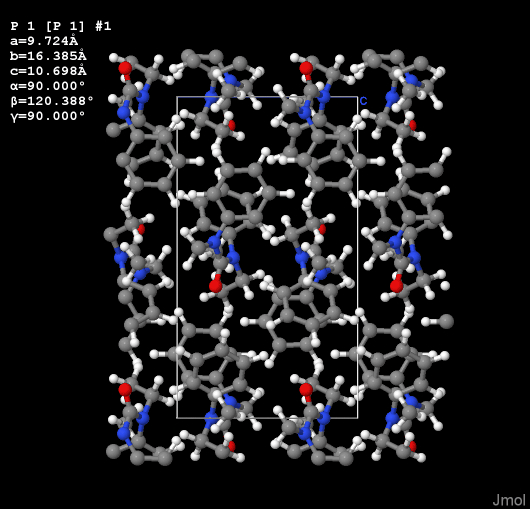

Supplement: Supplementary file 1 [file ao5c10754_si_001.zip › Select_DEDPU_Animations/Raman/103.90 - Mode 42/103 - Mode 42.gif]

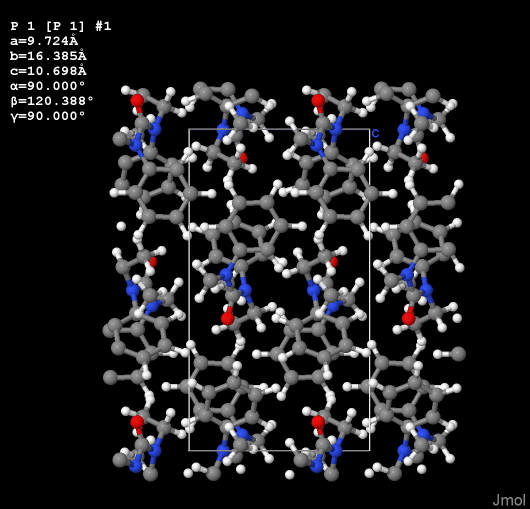

Supplement: Supplementary file 1 [file ao5c10754_si_001.zip › Select_DEDPU_Animations/Raman/123.79 - Mode 49/123 - Mode 49.gif]

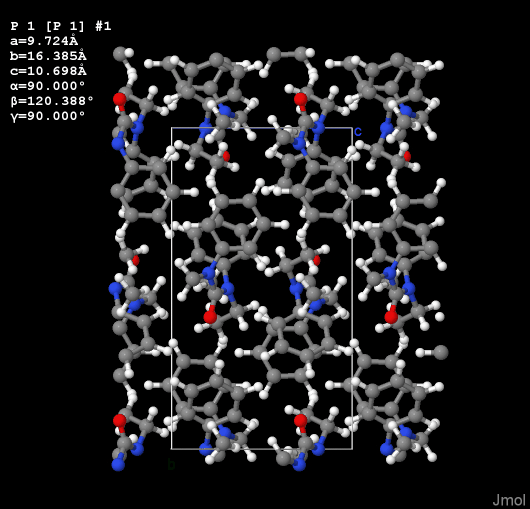

Supplement: Supplementary file 1 [file ao5c10754_si_001.zip › Select_DEDPU_Animations/Raman/124.54 - Mode 50/124 - Mode 50.gif]

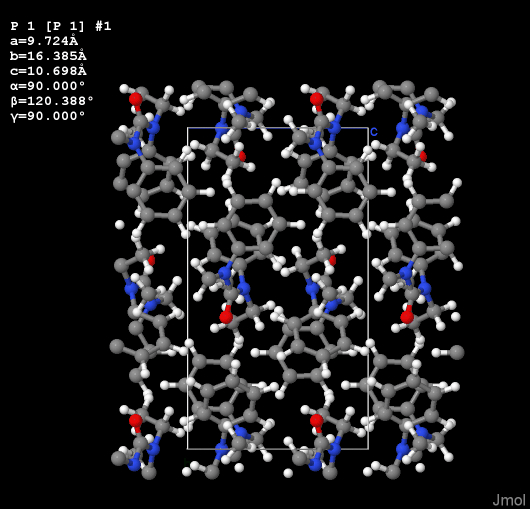

Supplement: Supplementary file 1 [file ao5c10754_si_001.zip › Select_DEDPU_Animations/Raman/92.23 - Mode 36/92 - Mode 36.gif]

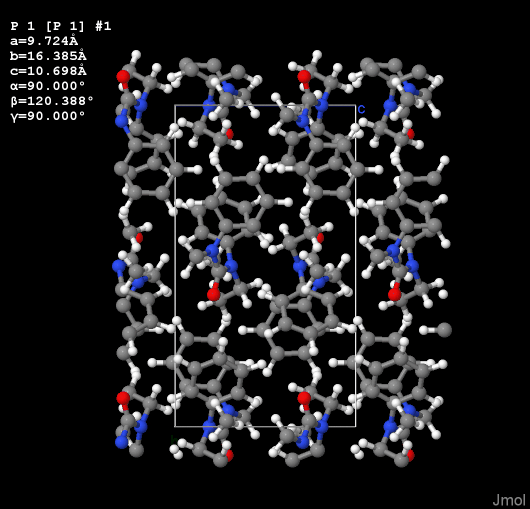

Supplement: Supplementary file 1 [file ao5c10754_si_001.zip › Select_DEDPU_Animations/THz/128.91 - Mode 52/128 -Mode 52.gif]

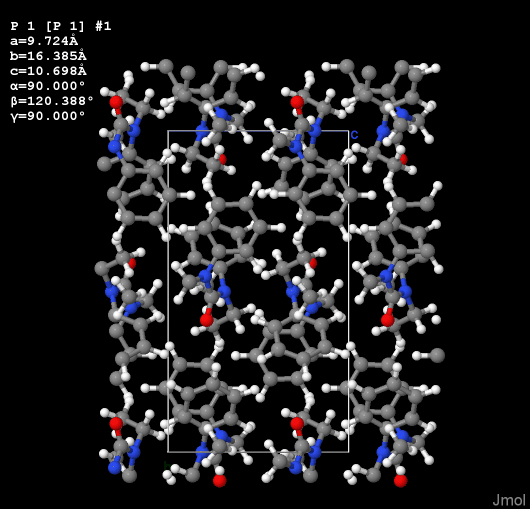

Supplement: Supplementary file 1 [file ao5c10754_si_001.zip › Select_DEDPU_Animations/THz/130.18 - Mode 53/130 - Mode 53.gif]

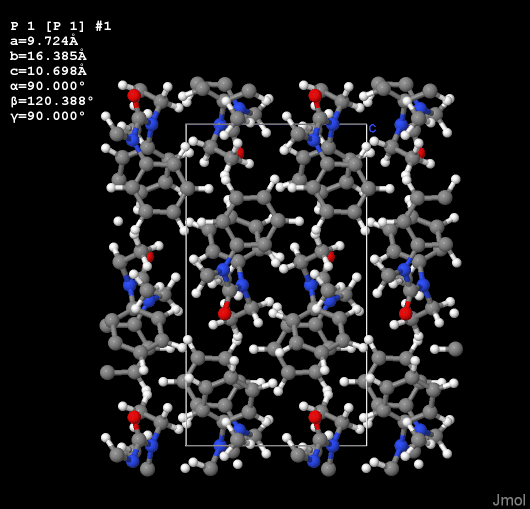

Supplement: Supplementary file 1 [file ao5c10754_si_001.zip › Select_DEDPU_Animations/THz/78.35 - Mode 28/78 - Mode 28.gif]

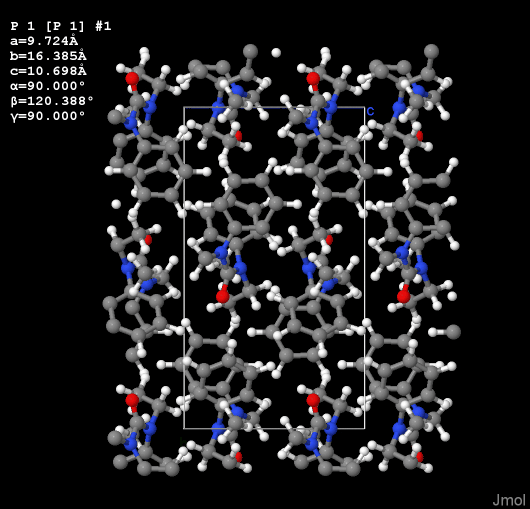

Supplement: Supplementary file 1 [file ao5c10754_si_001.zip › Select_DEDPU_Animations/THz/93.85 - Mode 37/93 - Mode 37.gif]

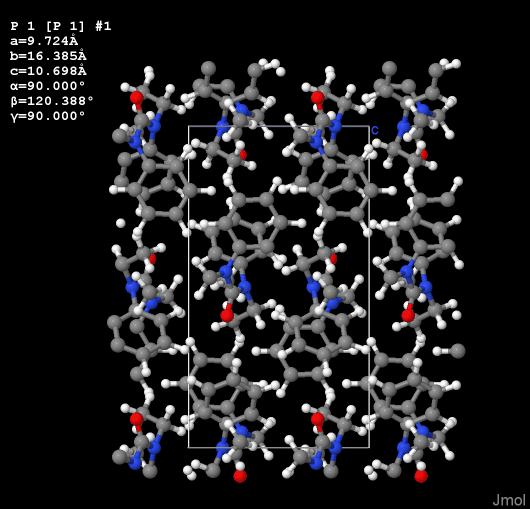

Supplement: Supplementary file 1 [file ao5c10754_si_001.zip › Select_DEDPU_Animations/THz/94.22 - Mode 38/94 - Mode 38.gif]

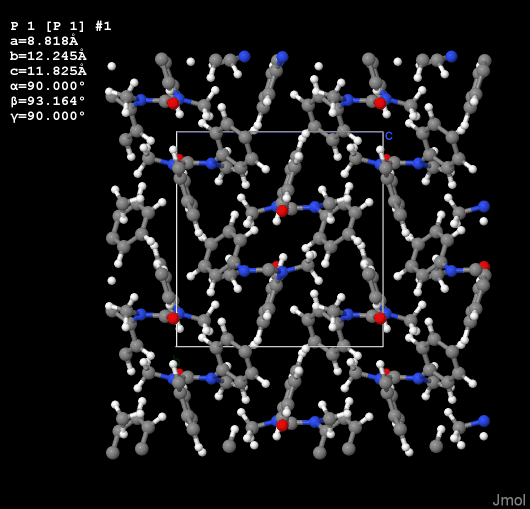

Supplement: Supplementary file 2 [file ao5c10754_si_002.zip › Select_DMDPU_Animations/Raman/101.71 - Mode 34/101 - Mode 34.gif]

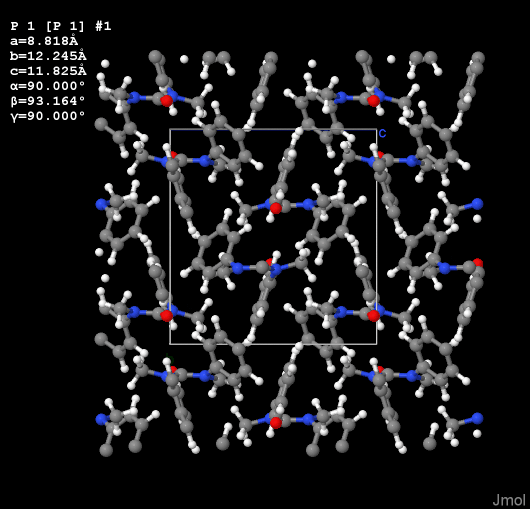

Supplement: Supplementary file 2 [file ao5c10754_si_002.zip › Select_DMDPU_Animations/Raman/102.69 - Mode 35/102 - Mode 35.gif]

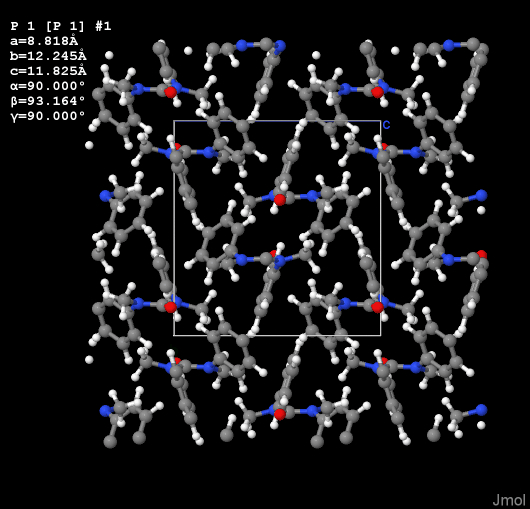

Supplement: Supplementary file 2 [file ao5c10754_si_002.zip › Select_DMDPU_Animations/Raman/118.37 - Mode 45/118 - Mode 45.gif]

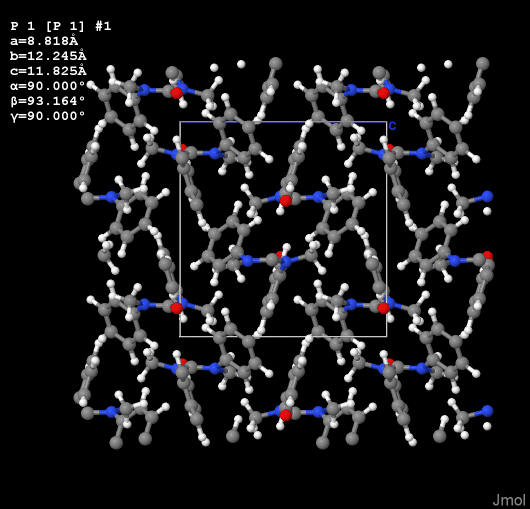

Supplement: Supplementary file 2 [file ao5c10754_si_002.zip › Select_DMDPU_Animations/Raman/119.08 - Mode 46/119 - Mode 46.gif]

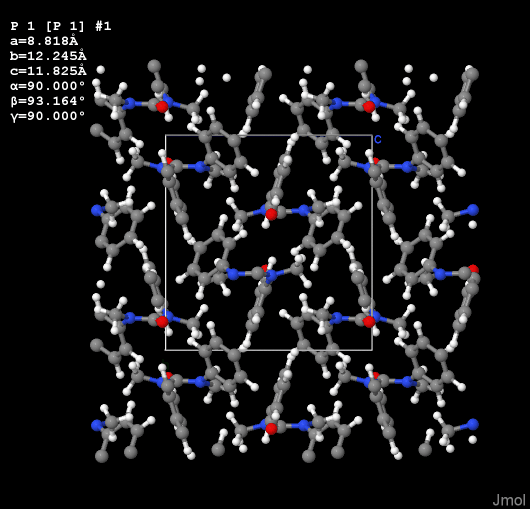

Supplement: Supplementary file 2 [file ao5c10754_si_002.zip › Select_DMDPU_Animations/Raman/76.12 - Mode 26/76 - Mode 26.gif]

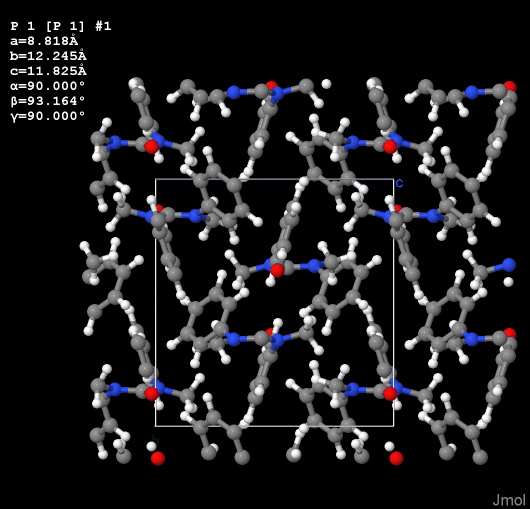

Supplement: Supplementary file 2 [file ao5c10754_si_002.zip › Select_DMDPU_Animations/THz/105.82 - Mode 36/105 - Mode 36.gif]

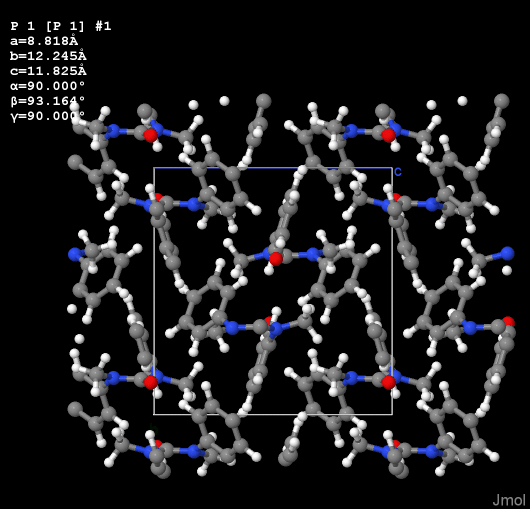

Supplement: Supplementary file 2 [file ao5c10754_si_002.zip › Select_DMDPU_Animations/THz/115.31 - Mode 43/115 - Mode 43.gif]

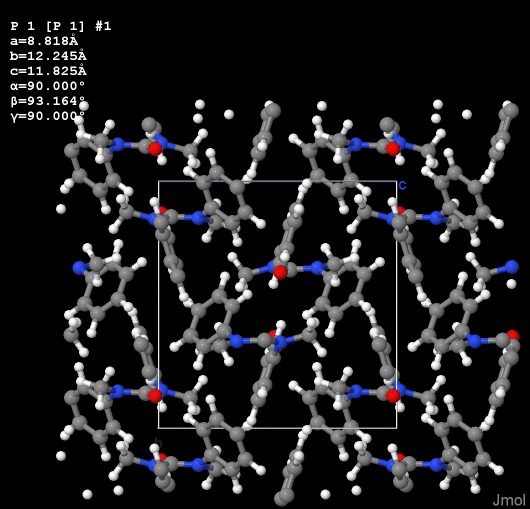

Supplement: Supplementary file 2 [file ao5c10754_si_002.zip › Select_DMDPU_Animations/THz/119.11 - Mode 47/119 - Mode 47.gif]

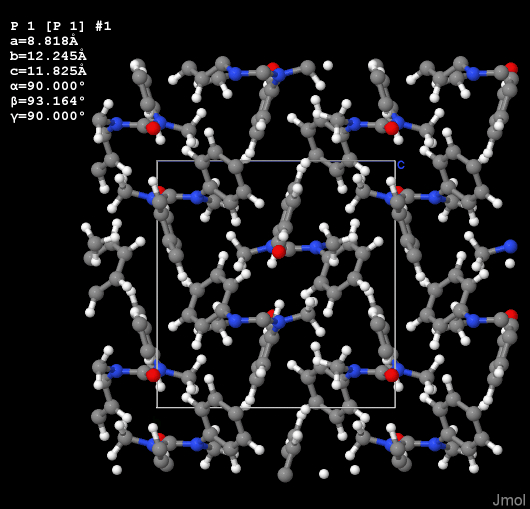

Supplement: Supplementary file 2 [file ao5c10754_si_002.zip › Select_DMDPU_Animations/THz/127.63 - Mode 50/127 - Mode 50.gif]

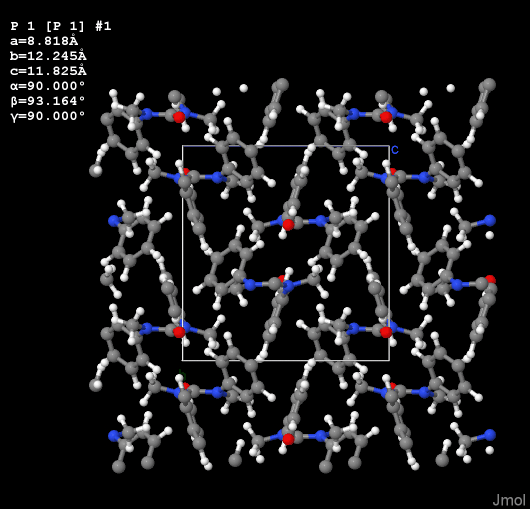

Supplement: Supplementary file 2 [file ao5c10754_si_002.zip › Select_DMDPU_Animations/THz/77.67 - Mode 27/77 - Mode 27.gif]
