# Supplementary material for: Characterizing Organic Gunshot Residues with Low-Frequency Raman and Terahertz Vibrational Spectroscopies
Source: ACS Omega. 2026 Jan 23;11(5):8209–21. doi: 10.1021/acsomega.5c10754 (PMC12903151; doi:10.1021/acsomega.5c10754)
Supplement: Supplementary file 3 [file ao5c10754_si_003.pdf]

Characterizing Organic Gunshot Residues with Low-frequency Raman and Terahertz Vibrational Spectroscopies

Salvatore Zarrella, Margaret P. Davis, Mary N. Boyden, and Timothy M. Korter*

Department of Chemistry, Syracuse University, 3-014 Center for Science and Technology, Syracuse, New York 13244-4100, United States

*Email: tmkorter@syr.edu

ORCID ID: Salvatore Zarrella 0009-0004-5576-3803
ORCID ID: Margaret P. Davis 0000-0003-0191-064X
ORCID ID: Mary N. Boyden 0000-0002-0530-5984
ORCID ID: Timothy M. Korter 0000-0002-0398-5680

Supporting Information

Table S1. Experimental low-frequency Raman peak centers at 295 K and 78 K for DEDPU and DMDPU.

Table S2. Experimental THz-TDS peak centers at 295 K and 78 K for DEDPU and DMDPU.

Table S3. Experimental mid-frequency Raman peak centers for DEDPU and DMDPU at 295 K.

Table S4. Experimental and ss-DFT optimized lattice parameters for DEDPU and DMDPU.

Table S5. BSSE corrected cohesion energies and contribution to ss-DFT calculations for DEDPU and DMDPU.

Table S6. RMSD values for DEDPU and DMDPU.

Tables S7 – S11. Line-shape analysis LFRS data for all molar ratios of DEDPU:DMDPU.

Tables S12 - S13. Line-shape analysis LFRS data for pure OGSRs at 295 K.

Tables S14 - S15. Line-shape analysis LFRS data for pure OGSRs at 78 K.

Table S16. Line-shape analysis THz data for equimolar DEDPU:DMDPU mixture.

Tables S17 - S18. Line shape analysis THz data for pure DMDPU at 295 K and 78 K.

Tables S19 – S20. Line shape analysis THz data for pure DEDPU at 295 K and 78 K.

Table S21. ss-DFT IR-active modes with frequencies (cm^{-1}), intensities (km/mol), and mode symmetries for DEDPU full optimization.

Table S22. ss-DFT Raman-active modes with frequencies (cm^{-1}), intensities (arb. units), and mode symmetries for DEDPU full optimization.

Table S23. ss-DFT IR-active modes with frequencies (cm^{-1}), intensities (km/mol), and mode symmetries for DMDPU full optimization.

Table S24. ss-DFT Raman-active modes with frequencies (cm^{-1}), intensities (arb. units), and mode symmetries for DMDPU full optimization.

Table S25. Linear Regression for Raman active peak at 98.8 cm^{-1} (DEDPU).

Table S26. Linear Regression for Raman active peak at 111.7 cm^{-1} (DMDPU).

Table S27. Linear Regression for Raman active peak at 247.6 cm^{-1} (DEDPU).

Table S28. Linear Regression for Raman active peak at 260.1 cm^{-1} (DEDPU).

Table S29. Linear Regression for Raman active peak at 297.6 cm^{-1} (DEDPU).

Table S30. THz pellet specifications for the corresponding data in Figures 5 and 7 of the manuscript.

Table S31. Signal-to-noise values for LFRS and THz data sets at 295 K and 78 K for DEDPU and DMDPU.

Figure S1. Uncorrected mid-frequency Raman data for DEDPU (orange) and DMDPU (purple) at 295 K.

Figure S2. Uncorrected mid-frequency Raman data for DEDPU (orange) and DMDPU (purple) at 295 K with peak centers.

Figure S3. Baseline corrected and normalized mid-frequency Raman spectra for DEDPU (orange) and DMDPU (purple).

Figures S4 – S5. 295 K IR spectra of DEDPU and DMDPU respectively.

Figures S6 – S7. Baseline-uncorrected 295 K (red) and 78 K (blue) LFRS data for DMDPU and DEDPU respectively. Spectra have been normalized to 1.

Figures S8 -S9. 3-D crystallographic unit cells of DEDPU and DMDPU respectively.

Figures S10 – S11. Experimental PXRD pattern (black) compared to CSD predicted pattern (red) of DEDPU and DMDPU respectively. Intensities have been normalized to 1.

Figure S12. Comparison of PXRD data of equimolar DEDPU:DMDPU mixture (black) to pure DEDPU (red) and pure DMDPU (blue). Data taken at 295 K.

Figure S13. Fitted 295 K Raman spectra for pure DEDPU in the 10-200 cm^{-1} region.

Figure S14. Fitted 295 K Raman spectra for pure DEDPU in the 200-305 cm^{-1} region.

Figures S15 – S16. Fitted 78 K Raman spectra for pure DEDPU in both 10-200 and 200-305 cm^{-1} region.

Figures S17 – S18. Fitted 295 K Raman spectra for pure DMDPU in both 10-200 and 200-305 cm^{-1} region.

Figures S19 – S20. Fitted 78 K Raman spectra for pure DMDPU in both 10-200 and 200-305 cm^{-1} region.

Figure S21. Fitted 295 K THz spectrum of pure DEDPU

Figure S22. Fitted 78 K THz spectrum of pure DEDPU

Figure S23. Fitted 295 K THz spectrum of pure DMDPU

Figure S24. Fitted 78 K THz spectrum of pure DMDPU

Figures S25. Fitted 78 K THz spectrum of the equimolar mix of DEDPU:DMDPU

Figures S26 – S27. Fitted 78 K Raman spectra for 0.10 molar mixture of DEDPU:DMDPU

Figures S28 – S29. Fitted 78 K Raman spectra for 0.25 molar mixture of DEDPU:DMDPU

Figures S30 – S31. Fitted 78 K Raman spectra for 0.50 molar (equimolar) mixture of DEDPU:DMDPU

Figures S32- S33. Fitted 78 K Raman spectra for 0.75 molar mixture of DEDPU:DMDPU

Figures S34- S35. Fitted 78 K Raman spectra for 0.90 molar mixture of DEDPU:DMDPU

Figure S36. Peaks of DEDPU across all molar mixtures in the 200-305 cm^{-1} region and their respective LODs.

Figure S37. Typical THz-TDS pellet with corresponding pellet dimensions for each OGSR

Table S1. List of the fitted experimental Raman peak centers at 295 K and 78 K for DEDPU and DMDPU. Values are rounded to 1 decimal place to match significant figures of instrument resolution.

Peak #	DEDPU 295 K	DEDPU 78 K	DMDPU 295 K	DMDPU 78 K
	Peak center (cm^{-1})		Peak center (cm^{-1})	
1	25.4	23.9	29.6	34.7
2	32.1	32.9	34.5	42.4
3	42.6	38.4	43.2	49.3
4	67.0	46.8	47.8	60.6
5	82.0	54.1	61.3	62.5
6	93.6	63.4	70.8	65.3
7	116.1	69.4	89.5	73.8
8	130.0	79.7	93.8	76.2
9	150.5	82.9	103.1	86.0
10		90.4	109.6	90.2
11		98.8	125.0	104.2
12		107.5		105.7
13		120.4		108.0
14		123.3		111.7
15		136.6		114.9
16		138.7		120.1
17		152.1		120.1
18				130.8

Table S2. List of the fitted experimental THz peak centers at 295 K and 78 K for DEDPU and DMDPU. Values are rounded to 1 decimal place to match significant figures of instrument resolution.

Peak Number	DEDPU 295 K	DEDPU 78 K	DMDPU 295 K	DMDPU 78 K
	Peak center (THz)		Peak center (THz)	
1	1.5	1.1	1.1	1.1
2	2.7	1.5	1.6	1.2
3	2.9	1.9	1.8	1.6
4	3.6	2.7	3.0	1.7
5		3.0	3.1	1.9
6		3.1	3.9	2.3
7		3.8	4.0	2.7
8				3.1
9				3.2
10				3.4
11				3.6
12				4.0

Table S3. List of experimental high-frequency Raman peak centers at 295 K for DEDPU and DMDPU.

DEDPU	DMDPU
Peak Center (cm^{-1})	Peak Center (cm^{-1})
238.08	226.30
298.26	324.49
430.00	393.54
553.09	450.40
606.42	484.23
617.38	597.63
708.59	615.19
777.03	712.89
976.78	772.75
1005.25	946.09
1077.70	1003.22
1156.94	1025.48
1170.68	1153.01
1259.94	1319.16
1358.84	1591.75
1437.20	
1591.75	

Table S4. Comparison of experimental and ss-DFT optimized crystallographic unit cell dimensions (Å), angles (°), volumes (V, Å^3), and densities (D, g/cm^3) of DMDPU and DEDPU.

	DMDPU			DEDPU		
	Exp.[a]	ss-DFT	Diff.	Exp.[b]	ss-DFT	Diff.
a	8.84721	8.81845	-0.33	9.699	9.72377	0.26
b	12.3542	12.24483	-0.89	16.7622	16.38538	-2.25
c	11.745	11.8249	0.68	10.6011	10.69848	0.92
α	90	90	-	90	90	-
β	92.445	93.161	0.77	118.854	120.388	-
γ	90	90	-	90	90	-
V	1282.562	1274.910	-0.60	1509.523	1470.384	-2.59
D	1.244	1.251	0.56	1.181	1.211	2.54

[a]Betz, R.; Gerber, T.; Schalekamp, H., 1,3-Diethyl-1,3-diphenylurea. *Acta Crystallographica Section E* **2011,** *67* (4), o827.
[b]Yamasaki, R.; Iida, M.; Ito, A.; Fukuda, K.; Tanatani, A.; Kagechika, H.; Masu, H.; Okamoto, I., Crystal Engineering of N,N′-Diphenylurea Compounds Featuring Phenyl–Perfluorophenyl Interaction. *Crystal Growth & Design* **2017,** *17* (11), 5858-5866.

Table S5. Final BSSE-corrected cohesion energy per molecule with DFT-D3-ABC and percent of BSSE contribution to the original uncorrected cohesion energy.

Molecule	Uncorrected Cohesion Energy per Molecule (kJ/mol)	BSSE-Corrected Cohesion Energy per Molecule (kJ/mol)	% BSSE Contribution
DMDPU	-119.7564	-112.1084	6.39
DEDPU	-119.2058	-112.6878	5.47

Table S6. RMSD values from analyses of differences in bond lengths, bond angle, and torsions between experimental DMDPU, DEDPU, and ss-DFT optimized crystal structures.

	DMDPU	DEDPU
Bond lengths (Å)	0.010	0.013
Bond angles (°)	0.317	0.320
Torsional angles (°)	3.143	3.576

Table S7. Resulting LFRS fit data for 0.10 molar DEDPU:DMDPU mixture (full spectrum).

Peak #	Peak Center (cm^{-1})	Peak Center Std. Deviation	Gaussian:Lorentzian Ratio
1	34.89592	0.02563	1:1
2	42.82765	0.06199	1:1
3	49.51558	0.03962	1:1
4	58.08772	0.12572	1:1
5	65.17971	0.04715	1:1
6	75.49744	0.02326	1:1
7	86.06785	0.21251	0.60:1
8	91.85634	0.1014	0.95:1
9	101.37481	0.12192	1.18:1
10	111.10258	0.27116	0.80:1
11	116.14753	0.11997	0.92:1
12	121.37513	0.25938	0.84:1
13	145.57444	0.62662	0.82:1
14	152.90646	0.693	1:1
15	170.80589	0.63514	1.30:1
16	185.38722	0.43378	1.67:1
17	228.35268	0.04164	1:1
18	232.61366	0.06048	0.75:1
19	249.50746	0.29177	2:1
20	267.36326	2.73453	1:1
21	274.66216	2.4277	1:1
22	281.13431	0.67266	0.90:1
23	298.51319	0.33828	0.90:1

Table S8. Resulting LFRS fit data for 0.25 molar DEDPU:DMDPU mixture (full spectrum).

Peak #	Peak Center (cm^{-1})	Peak Center Std. Deviation	Gaussian:Lorentzian Ratio
1	34.79145	0.02764	2.5:1
2	42.58467	0.08744	0.80:1
3	49.39104	0.07801	1.25:1
4	52.32294	0.53095	1:1
5	57.59472	0.16966	0.75:1
6	62.18375	0.35709	0.85:1
7	65.18797	0.20376	0.85:1
8	75.43115	0.06017	1:1
9	80.90412	0.23331	1:1
10	86.76138	0.61952	0.85:1
11	92.05361	0.13567	1:1
12	101.29672	0.08339	1:1
13	110.82393	0.16791	0.75:1
14	117.56639	0.2344	0.70:1
15	124.00548	0.33788	0.75:1
16	142.2213	1.2693	1:1
17	143.60066	2.24389	1:1
18	228.17604	0.06112	0.75:1
19	232.54802	0.06594	1:1
20	249.46974	0.20683	1.16:1
21	260.21899	0.44276	0.75:1
22	280.20032	0.24178	0.66:1
23	298.50575	0.21576	0.67:1

Table S9. Resulting LFRS fit data for equimolar DEDPU:DMDPU mixture (full spectrum).

Peak #	Peak Center (cm^{-1})	Peak Center Std. Deviation	Gaussian:Lorentzian Ratio
1	23.77217	0.04924	1.35:1
2	33.19668	0.01114	1:1
3	39.56295	0.03681	0.70:1
4	47.71276	0.023	1.40:1
5	55.79132	0.06828	0.80:1
6	63.52563	0.14041	0.80:1
7	67.17657	0.42978	0.90:1
8	74.38403	0.04961	1:1
9	79.68601	0.13942	1:1
10	87.75176	0.55899	0.67:1
11	90.90849	0.08581	0.80:1
12	99.28431	0.0154	1:1
13	108.96814	0.24754	0.75:1
14	109.95136	0.98425	0.80:1
15	115.90511	0.26064	0.85:1
16	119.84311	0.95542	0.70:1
17	124.37344	0.58808	0.80:1
18	138.1094	0.15735	0.85:1
19	144.07868	0.5065	0.80:1
20	152.34113	0.14372	1:1
21	226.81169	0.05624	1:1
22	231.41153	0.09835	0.67:1
23	248.01206	0.06891	1:1
24	258.12984	0.71893	0.80:1
25	260.38359	0.66706	1:1
26	279.6395	0.56299	0.85:1
27	297.52864	0.0586	1:1

Table S10. Resulting LFRS fit data for 0.75 molar DEDPU:DMDPU mixture (full spectrum).

Peak #	Peak Center (cm^{-1})	Peak Center Std. Deviation	Gaussian:Lorentzian Ratio
1	23.71041	0.03683	0.65:1
2	33.12842	0.00905	1:1
3	39.11193	0.01577	1:1
4	47.4918	0.01956	1:1
5	55.33492	0.05052	0.65:1
6	63.79109	0.03899	0.65:1
7	72.00288	0.17121	0.85:1
8	75.46168	0.13256	1:1
9	79.6158	0.14212	1:1
10	83.50713	0.10678	1:1
11	90.80258	0.02194	1:1
12	99.35165	0.01278	1:1
13	107.70075	0.14154	0.95:1
14	114.15353	0.16978	0.85:1
15	119.46185	0.23704	0.85:1
16	123.61914	0.12827	0.67:1
17	137.8437	0.06353	0.70:1
18	143.35435	0.18181	0.80:1
19	152.52817	0.05718	1:1
20	225.51336	0.0524	1.10:1
21	231.52464	0.20481	0.75:1
22	248.01996	0.03966	0.70:1
23	258.34398	0.47905	1:1
24	260.59038	0.72739	0.90:1
25	297.5741	0.03172	1:1

Table S11. Resulting LFRS fit data for 0.90 molar DEDPU:DMDPU mixture (full spectrum).

Peak #	Peak Center (cm^{-1})	Peak Center Std. Deviation	Gaussian:Lorentzian Ratio
1	24.91538	0.16198	1.25:1
2	34.03832	0.0349	1:1
3	39.64586	0.0858	0.60:1
4	48.11082	0.08993	1:1
5	55.7528	0.33078	1:1
6	67.39724	0.25304	0.80:1
7	76.7527	0.16737	1:1
8	81.28803	0.19093	1:1
9	91.54165	0.12211	0.80:1
10	100.00475	0.0311	0.75:1
11	108.93081	0.18926	0.95:1
12	115.44568	0.21927	1:1
13	122.3529	0.33865	1:1
14	124.04333	1.18228	1:1
15	137.90684	0.36586	0.80:1
16	142.74259	1.42859	0.70:1
17	153.01255	0.29651	0.80:1
18	225.85827	0.21353	0.75:1
19	230.02455	0.43746	1:1
20	248.5285	0.0918	0.75:1
21	260.10434	0.10791	1:1
22	268.4137	0.84894	1:1
23	298.55151	0.04858	1:1

Table S12. Resulting LFRS fit data for pure DMDPU at 295 K (full spectrum).

Peak #	Peak Center (cm^{-1})	Peak Center Std. Deviation	Gaussian:Lorentzian Ratio
1	29.6247	0.08532	1:1
2	34.54502	0.1192	1:1
3	43.15097	0.19115	0.75:1
4	47.7751	0.76609	0.80:1
5	61.25492	0.19311	0.67:1
6	70.81966	0.18967	0.85:1
7	89.47607	0.43472	1:1
8	93.75198	0.44794	1:1
9	103.08306	0.57222	0.70:1
10	109.56081	0.90546	0.75:1
11	125.03327	0.2632	0.95:1
12	188.34261	1.12258	1:1
13	226.49787	0.11216	0.01:1
14	227.67454	0.15006	1.50:1

Table S13 Resulting LFRS fit data for pure DEDPU at 295 K (full spectrum).

Peak #	Peak Center (cm^{-1})	Peak Center Std. Deviation	Gaussian:Lorentzian Ratio
1	25.43919	0.60479	0.75:1
2	32.13853	0.08559	1:1
3	42.59853	0.08559	0.80:1
4	66.99324	0.34172	0.90:1
5	82.04588	0.14671	0.67:1
6	93.5764	0.07152	1.40:1
7	116.0558	0.61792	1.10:1
8	129.99983	0.51423	1.10:1
9	150.50531	0.63305	0.75:1
10	241.58731	0.64998	0.75:1
11	245.95105	0.55399	0.95:1
12	256.9155	0.60025	0.80:1
13	260.34642	0.72896	0.80:1
14	297.70627	0.03022	1.10:1

Table S14. Resulting LFRS fit data for pure DMDPU at 78 K (full spectrum).

Peak #	Peak Center (cm^{-1})	Peak Center Std. Deviation	Gaussian:Lorentzian Ratio
1	34.66838	0.03913	1:1
2	42.35361	0.09739	0.75:1
3	49.34499	0.05694	1.50:1
4	60.64865	2.08126	0.75:1
5	62.52038	0.42918	0.85:1
6	65.28419	0.3992	0.80:1
7	73.83979	0.21986	0.67:1
8	76.18866	0.08198	1:1
9	85.97052	0.30091	1:1
10	90.22715	0.28465	0.75:1
11	104.2353	3.48324	0.67:1
12	105.67814	1.57824	0.70:1
13	107.96817	1.14537	0.70:1
14	111.65898	1.00868	0.70:1
15	114.86022	0.74978	0.85:1
16	120.11095	0.52078	0.60:1
17	120.12891	0.73995	1:1
18	130.80314	0.39909	0.87:1
19	228.46465	0.18248	0.80:1
20	233.12273	0.12054	1.67:1
21	253.37332	0.24142	0.80:1
22	266.4422	0.42402	1:1
23	273.98613	0.39899	1:1
24	281.25175	0.22142	1:1

Table S15. Resulting LFRS fit data for pure DEDPU at 78 K (full spectrum).

Peak #	Peak Center (cm^{-1})	Peak Center Std. Deviation	Gaussian:Lorentzian Ratio
1	23.92143	0.14912	1:1
2	32.93681	0.03629	1:1
3	38.42717	0.06781	0.70:1
4	46.84194	0.0831	0.70:1
5	54.08928	0.20187	0.85:1
6	63.39587	0.25592	0.60:1
7	69.3903	0.30777	0.80:1
8	79.66216	0.47661	1:1
9	82.94979	0.64012	0.85:1
10	90.35978	0.16411	0.90:1
11	98.78896	0.03193	1:1
12	107.48036	0.25598	0.70:1
13	120.39939	1.17321	0.85:1
14	123.33193	0.31366	0.75:1
15	136.61198	2.19435	1.10:1
16	138.67139	3.44042	0.85:1
17	152.12946	0.21375	0.75:1
18	222.98801	0.47122	0.67:1
19	225.39161	0.14713	0.75:1
20	247.64614	0.03553	1.10:1
21	257.79853	0.53953	0.70:1
22	260.09776	0.50734	0.60:1
23	297.56535	0.02883	1.25:1

Table S16. Resulting THz-TDS fit data for 50-50 DMDPU:DEDPU mixture at 78 K.

Peak #	Peak Center (THz)	Peak Center Std. Deviation	Gaussian:Lorentzian Ratio
1	1.1868	7.45761E-4	0.75:1
2	1.53177	0.00189	1.20:1
3	1.67996	0.0021	0.60:1
4	1.88884	0.00106	1.10:1
5	2.74366	8.21504E-4	0.67:1
6	2.96504	0.00524	1:1
7	3.10008	0.00793	1.60:1
8	3.41934	0.00194	1:1
9	3.77819	0.00342	1.30:1
10	3.86188	0.00753	1.20:1
11	3.99504	0.00395	1:1

Table S17. Resulting THz-TDS fit data for Pure DMDPU at 295 K.

Peak #	Peak Center (THz)	Peak Center Std. Deviation	Gaussian:Lorentzian Ratio
1	1.10808	0.00123	0.75:1
2	1.64259	0.00363	0.65:1
3	1.77864	0.00428	0.75:1
4	3.00886	0.0028	0.85:1
5	3.06553	0.01325	1.40:1
6	3.89553	0.00373	0.08:1
7	3.97105	0.00165	1:1

Table S18. Resulting THz-TDS fit data for Pure DMDPU at 78 K.

Peak #	Peak Center (THz)	Peak Center Std. Deviation	Gaussian:Lorentzian Ratio
1	1.10807	0.00307	0.85:1
2	1.21256	6.91708E-4	0.67:1
3	1.56437	0.00383	1:1
4	1.70229	9.22844E-4	0.10:1
5	1.88806	0.00164	0.80:1
6	2.33614	0.00208	0.10:1
7	2.69672	0.00311	0.75:1
8	3.03257	0.01591	0.60:1
9	3.14779	0.00415	0.55:1
10	3.44733	0.00245	1.33:1
11	3.98404	5.1292E-4	1.20:1

Table S19. Resulting THz-TDS fit data for Pure DEDPU at 295 K.

Peak #	Peak Center (THz)	Peak Center Std. Deviation	Gaussian:Lorentzian Ratio
1	1.53809	0.00637	0.75:1
2	2.6778	0.00359	0.75:1
3	2.88398	0.00284	0.85:1
4	3.61341	0.00356	0.80:1

Table S20. Resulting THz-TDS fit data for Pure DEDPU at 78 K.

Peak #	Peak Center (THz)	Peak Center Std. Deviation	Gaussian:Lorentzian Ratio
1	1.54223	0.00108	1:1
2	1.89065	0.00103	0.75:1
3	2.74073	4.95721E-4	0.75:1
4	2.96139	2.14118E-4	0.10:1
5	3.0881	5.14364E-4	1:1
6	3.82108	3.83979E-4	0.70:1

Table S21. ss-DFT IR-active modes with frequencies (cm^{-1}), intensities (km/mol), and mode symmetries for DEDPU full optimization.

Mode Label (ν)	Frequency (cm^{-1})	Intensity (km/mol)	Mode Symm.	Mode Label (ν)	Frequency (cm^{-1})	Intensity (km/mol)	Mode Symm.
5	26.67	0.22	Au	81	284.81	0.13	Bu
7	32.08	0.01	Au	82	286.50	0.43	Au
9	36.27	0.30	Bu	85	307.64	6.77	Bu
14	50.35	0.86	Au	87	309.43	0.25	Au
15	50.61	1.06	Bu	91	344.68	0.00	Au
18	57.52	1.06	Au	92	345.80	0.58	Bu
19	60.36	0.62	Bu	93	399.46	4.70	Au
20	64.60	0.26	Au	94	400.20	57.91	Bu
24	68.38	0.53	Bu	97	409.44	0.15	Au
26	69.87	0.37	Au	99	411.70	0.11	Au
28	78.35	5.27	Bu	102	412.18	0.18	Bu
30	80.86	0.25	Au	103	418.19	7.46	Bu
33	84.24	1.38	Bu	107	427.96	12.40	Au
34	87.04	3.25	Au	108	428.12	11.34	Bu
37	93.85	13.21	Bu	111	429.75	28.24	Au
38	94.22	16.47	Bu	112	429.96	28.86	Bu
39	97.21	1.08	Au	115	446.60	22.39	Bu
40	99.68	0.73	Au	116	446.70	1.87	Au
43	107.13	1.20	Bu	119	479.01	3.51	Bu
45	110.21	0.02	Au	120	479.25	42.75	Au
47	111.82	0.41	Bu	122	486.81	1.33	Bu
51	128.37	11.00	Bu	124	491.24	0.02	Au
52	128.91	0.38	Au	126	543.90	264.30	Bu
53	130.18	16.23	Bu	128	547.54	19.61	Au
54	133.04	0.00	Au	130	598.95	100.21	Au
57	138.12	56.19	Bu	131	599.04	8.34	Bu
60	144.79	1.27	Au	133	612.90	11.39	Bu
63	155.08	0.08	Bu	135	614.45	1.66	Bu
64	155.64	5.18	Au	136	614.55	0.55	Au
65	205.94	3.46	Au	139	616.84	6.28	Au
67	206.28	1.05	Bu	141	635.04	1.19	Au
69	224.85	2.31	Bu	143	637.95	12.47	Bu
70	225.06	0.00	Au	146	684.02	586.91	Bu
73	242.36	6.23	Bu	148	690.67	21.49	Au
75	243.51	0.12	Au	149	694.64	173.90	Au
79	254.28	5.80	Au	152	696.05	56.71	Bu
80	256.08	25.03	Bu	153	704.14	0.40	Au

156	705.30	2.34	Bu	237	1025.16	80.41	Au
157	724.77	284.06	Bu	239	1025.75	8.79	Bu
158	726.66	7.71	Au	242	1037.12	3.80	Au
162	745.48	227.60	Bu	244	1038.10	44.87	Bu
164	751.63	15.45	Au	247	1066.15	5.77	Bu
165	753.95	4.49	Bu	248	1066.31	0.42	Au
167	755.83	11.50	Au	251	1073.42	23.38	Bu
169	757.70	12.00	Bu	252	1073.52	76.84	Au
170	762.74	137.53	Au	253	1074.72	136.38	Bu
173	770.19	103.12	Au	256	1077.10	32.20	Au
175	771.85	25.82	Bu	259	1081.23	9.35	Au
177	813.60	41.64	Au	260	1081.85	243.16	Bu
180	827.23	13.94	Bu	262	1105.58	0.36	Bu
183	829.06	0.22	Au	263	1105.60	11.60	Au
184	830.49	22.32	Bu	266	1115.28	268.69	Bu
185	835.01	0.04	Au	268	1116.87	13.70	Au
188	837.09	9.79	Bu	271	1144.38	24.72	Au
190	884.86	5.99	Au	272	1144.91	7.60	Bu
192	889.97	6.04	Bu	273	1150.36	48.08	Au
194	899.89	3.52	Bu	276	1153.50	1.49	Bu
196	901.67	3.52	Au	277	1159.33	17.52	Bu
197	916.36	63.46	Bu	281	1160.93	70.63	Au
198	916.49	1.34	Au	282	1162.09	39.59	Bu
201	932.21	3.01	Au	284	1162.51	79.87	Au
202	932.58	0.05	Bu	287	1178.95	73.35	Au
205	942.28	0.82	Bu	288	1179.58	9.91	Bu
207	942.83	0.30	Au	289	1235.09	136.76	Bu
209	951.18	0.64	Au	290	1235.32	3.67	Au
211	955.28	1.04	Bu	295	1249.32	297.85	Au
214	961.39	10.95	Bu	296	1250.13	32.39	Bu
215	963.53	19.97	Au	299	1267.93	1440.84	Bu
219	971.58	13.92	Au	300	1269.35	120.72	Au
220	972.35	1.75	Bu	302	1292.93	13.25	Bu
222	981.12	3.77	Bu	303	1293.63	11.83	Au
224	983.38	0.09	Au	307	1299.17	22.40	Au
225	993.82	0.10	Au	308	1299.71	207.27	Bu
227	994.57	10.81	Bu	311	1330.69	42.20	Au
230	995.84	4.01	Bu	312	1330.74	43.99	Bu
232	996.45	4.93	Au	313	1338.86	76.94	Bu
234	1022.12	72.56	Bu	315	1341.08	2.02	Au
236	1023.17	2.80	Au	317	1343.57	51.11	Au

320	1344.52	1.73	Au	402	2977.60	8.78	Au
322	1345.02	32.53	Bu	405	2983.99	15.82	Au
323	1345.54	35.09	Bu	407	2984.06	54.21	Bu
325	1347.47	30.15	Au	409	3004.14	148.46	Bu
326	1348.16	73.17	Bu	410	3004.40	8.17	Au
331	1359.50	56.44	Au	415	3010.17	85.67	Bu
332	1360.64	158.45	Bu	416	3010.23	0.04	Au
333	1360.89	12.42	Au	417	3047.91	0.42	Bu
334	1361.52	32.39	Bu	418	3047.95	75.84	Au
337	1373.98	271.77	Au	421	3053.24	25.13	Bu
338	1375.56	1777.30	Bu	422	3053.27	13.85	Au
341	1419.74	182.28	Au	425	3057.66	3.26	Au
342	1419.96	51.27	Bu	426	3057.67	183.99	Bu
347	1431.11	58.94	Au	430	3067.83	109.46	Bu
348	1431.16	328.91	Bu	431	3067.85	34.23	Au
350	1433.61	76.56	Au	434	3072.64	6.84	Bu
351	1433.63	24.42	Bu	436	3072.92	95.12	Au
354	1440.40	56.55	Bu	438	3074.18	43.59	Au
358	1442.07	24.82	Bu	440	3074.43	5.45	Bu
359	1442.37	0.10	Au	441	3105.07	20.74	Au
360	1443.45	0.18	Au	444	3105.46	8.06	Bu
363	1445.19	58.47	Au	445	3112.80	2.74	Au
364	1445.82	150.94	Bu	448	3113.24	22.27	Bu
365	1449.05	47.72	Bu	449	3114.13	17.45	Au
366	1449.14	1.30	Au	452	3114.70	11.79	Bu
367	1451.34	0.44	Bu	453	3118.28	52.08	Bu
369	1452.49	64.50	Au	454	3118.48	5.13	Au
375	1478.67	79.14	Au	458	3121.22	10.45	Bu
376	1479.19	144.93	Bu	459	3121.77	18.14	Au
378	1481.82	784.27	Au	463	3127.91	1.60	Bu
380	1483.57	41.22	Bu	464	3128.04	22.52	Au
383	1580.36	290.20	Au	467	3129.28	1.97	Bu
384	1581.16	41.20	Bu	468	3129.36	21.98	Au
386	1582.73	57.23	Au	470	3133.39	12.28	Bu
387	1582.95	36.55	Bu	473	3134.15	3.30	Au
391	1594.94	32.24	Bu	474	3134.46	6.16	Bu
392	1595.46	43.58	Au	475	3134.47	56.73	Au
395	1597.34	458.02	Au	478	3143.87	15.07	Au
396	1599.23	38.02	Bu	480	3144.40	0.88	Bu
397	1625.13	3006.35	Au				
400	1636.61	281.06	Bu				

Table S22. ss-DFT Raman-active modes with frequencies (cm^{-1}), intensities (arb. units), and mode symmetries for DEDPU full optimization. Intensities are scaled to a maximum of 1000.

Mode Label (ν)	Frequency (cm^{-1})	Intensity (arb. units)	Mode Symm.	Mode Label (ν)	Frequency (cm^{-1})	Intensity (arb. units)	Mode Symm.
4	16.52	282.35	Ag	71	225.24	116.30	Ag
6	31.82	685.16	Bg	72	225.38	82.82	Bg
8	34.54	103.65	Ag	74	243.32	0.17	Bg
10	42.01	48.90	Ag	76	245.69	191.05	Ag
11	46.17	234.53	Ag	77	251.75	91.68	Ag
12	47.46	0.46	Bg	78	253.48	75.52	Bg
13	48.47	77.72	Bg	83	287.25	32.44	Bg
16	52.40	40.14	Bg	84	287.41	101.02	Ag
17	52.92	57.77	Ag	86	308.92	2.79	Bg
21	64.93	51.17	Ag	88	309.54	5.37	Ag
22	65.40	204.90	Bg	89	342.70	20.31	Bg
23	67.00	108.50	Ag	90	343.78	49.29	Ag
25	69.63	67.59	Bg	95	401.56	41.28	Bg
27	70.86	17.45	Bg	96	401.58	5.73	Ag
29	78.55	392.03	Bg	98	410.05	3.39	Bg
31	81.37	67.25	Ag	100	411.86	42.69	Bg
32	83.99	124.67	Bg	101	411.87	84.44	Ag
35	92.05	48.61	Bg	104	418.63	18.65	Ag
36	92.23	670.80	Ag	105	426.78	469.75	Ag
41	100.64	1000.00	Bg	106	427.32	15.50	Bg
42	103.90	893.93	Ag	109	428.54	5.18	Ag
44	109.42	286.34	Ag	110	429.23	7.96	Bg
46	110.57	76.39	Bg	113	445.74	86.58	Bg
48	114.41	198.93	Ag	114	446.60	36.18	Ag
49	123.79	829.72	Ag	117	478.15	29.73	Ag
50	124.54	512.62	Bg	118	478.41	1.44	Bg
55	134.38	275.05	Bg	121	486.77	18.20	Ag
56	135.68	85.94	Ag	123	489.59	3.53	Bg
58	141.77	446.25	Ag	125	542.71	7.30	Ag
59	142.48	477.80	Bg	127	545.00	46.12	Bg
61	153.30	43.43	Bg	129	596.87	135.06	Ag
62	153.89	288.15	Ag	132	599.07	5.96	Bg
66	205.97	76.67	Ag	134	614.35	61.69	Ag
68	209.08	5.97	Bg	137	614.84	67.60	Ag
138	615.42	136.35	Bg	218	970.88	145.56	Ag
140	617.46	2.84	Bg	221	980.65	28.53	Ag

142	636.00	10.76	Bg	223	982.85	7.98	Bg
144	638.15	2.50	Ag	226	994.23	50.32	Bg
145	682.90	9.56	Ag	228	994.82	558.92	Ag
147	689.13	1.75	Bg	229	995.37	421.91	Ag
150	695.04	40.48	Ag	231	996.38	10.71	Bg
151	695.54	0.14	Bg	233	1022.09	109.36	Ag
154	704.51	405.07	Ag	235	1022.20	18.84	Bg
155	705.07	10.11	Bg	238	1025.40	0.43	Bg
159	727.03	4.09	Ag	240	1026.21	788.47	Ag
160	730.07	26.39	Bg	241	1037.00	13.53	Bg
161	744.67	11.38	Ag	243	1037.99	8.80	Ag
163	749.74	2.76	Bg	245	1064.97	403.90	Ag
166	755.03	7.26	Ag	246	1065.41	0.94	Bg
168	756.11	13.13	Bg	249	1073.09	10.87	Ag
171	762.85	2.00	Bg	250	1073.28	3.19	Bg
172	762.89	22.24	Ag	254	1075.38	46.04	Ag
174	771.02	48.02	Ag	255	1076.35	9.95	Bg
176	775.00	2.11	Bg	257	1080.10	1.20	Ag
178	813.66	1.78	Bg	258	1081.12	17.81	Bg
179	826.44	36.18	Bg	261	1105.41	141.81	Ag
181	827.29	20.20	Ag	264	1106.83	7.66	Bg
182	828.51	22.81	Ag	265	1115.25	28.86	Bg
186	835.23	4.54	Ag	267	1115.72	18.20	Ag
187	836.39	0.48	Bg	269	1143.77	7.37	Bg
189	884.25	2.89	Bg	270	1144.02	49.04	Ag
191	889.47	3.73	Ag	274	1150.49	65.81	Bg
193	899.70	6.09	Bg	275	1151.88	51.42	Ag
195	901.13	10.59	Ag	278	1160.08	75.79	Bg
199	916.80	10.60	Ag	279	1160.22	93.58	Ag
200	917.62	18.30	Bg	280	1160.47	55.03	Ag
203	933.95	97.12	Ag	283	1162.35	1.56	Bg
204	935.23	12.19	Bg	285	1177.21	75.99	Ag
206	942.62	2.28	Bg	286	1177.31	24.62	Bg
208	942.99	11.59	Ag	291	1236.21	84.24	Ag
210	951.35	1.06	Bg	292	1236.62	181.63	Bg
212	955.73	6.84	Ag	293	1247.56	752.28	Ag
213	961.21	7.03	Ag	294	1247.98	25.22	Bg
216	963.94	8.00	Bg	297	1263.91	25.50	Bg
217	970.25	10.12	Bg	298	1266.33	8.30	Ag
301	1292.87	28.53	Ag	382	1580.04	32.77	Bg
304	1294.19	6.98	Bg	385	1582.48	92.69	Bg

305	1298.54	3.90	Ag	388	1583.08	1.61	Ag
306	1298.73	26.95	Bg	389	1585.75	59.55	Bg
309	1330.14	58.82	Bg	390	1586.72	285.90	Ag
310	1330.39	146.87	Ag	393	1595.79	383.96	Bg
314	1339.01	12.77	Bg	394	1595.89	43.48	Ag
316	1341.26	38.75	Ag	398	1627.88	245.17	Ag
318	1343.60	76.55	Ag	399	1629.57	13.47	Bg
319	1344.45	6.40	Bg	403	2978.11	7.34	Bg
321	1344.82	48.98	Ag	404	2978.36	459.81	Ag
324	1346.46	7.96	Bg	406	2984.00	688.34	Ag
327	1348.78	22.45	Bg	408	2984.15	2.71	Bg
328	1349.05	74.35	Ag	411	3004.61	356.29	Ag
329	1357.35	5.04	Bg	412	3004.67	44.08	Bg
330	1358.29	17.16	Ag	413	3009.92	241.06	Ag
335	1365.74	181.44	Ag	414	3009.96	8.60	Bg
336	1366.00	20.64	Bg	419	3048.04	145.94	Bg
339	1377.27	42.49	Bg	420	3048.04	29.93	Ag
340	1378.46	5.44	Ag	423	3053.29	138.87	Ag
343	1424.74	4.85	Bg	424	3053.36	114.24	Bg
344	1424.85	79.82	Ag	427	3058.52	185.20	Ag
345	1427.37	51.79	Bg	428	3058.55	41.95	Bg
346	1427.41	97.42	Ag	429	3067.61	86.38	Ag
349	1432.92	87.69	Ag	432	3068.10	141.49	Bg
352	1433.65	38.49	Bg	433	3072.55	40.11	Ag
353	1440.32	17.23	Ag	435	3072.78	60.18	Bg
355	1441.21	17.07	Bg	437	3073.94	90.16	Ag
356	1441.95	12.52	Ag	439	3074.26	1.76	Bg
357	1441.95	10.32	Bg	442	3105.14	58.35	Bg
361	1443.85	12.36	Bg	443	3105.29	57.85	Ag
362	1444.08	2.83	Ag	446	3112.93	1.04	Bg
368	1452.04	4.02	Bg	447	3113.18	64.77	Ag
370	1452.59	51.23	Ag	450	3114.23	131.69	Bg
371	1453.53	91.77	Ag	451	3114.62	63.55	Ag
372	1454.23	5.85	Bg	455	3118.57	120.90	Ag
373	1477.79	2.74	Ag	456	3118.91	16.09	Bg
374	1478.45	8.50	Bg	457	3120.98	136.60	Ag
377	1481.70	36.14	Ag	460	3121.77	43.69	Bg
379	1483.47	1.22	Bg	461	3127.07	225.07	Ag
381	1579.36	41.17	Ag	462	3127.23	100.88	Bg
465	3128.76	99.18	Ag	476	3134.89	32.41	Bg
466	3128.98	134.98	Bg	477	3143.64	1.31	Bg

469	3133.23	50.88	Ag	479	3144.34	757.77	Ag
471	3133.84	46.52	Bg				
472	3134.03	549.11	Ag				

Table S23. ss-DFT IR-active modes with frequencies (cm^{-1}), intensities (km/mol), and mode symmetries for DMDPU full optimization.

Mode Label (ν)	Frequency (cm^{-1})	Intensity (km/mol)	Mode Symm.	Mode Label (ν)	Frequency (cm^{-1})	Intensity (km/mol)	Mode Symm.
5	36.35	0.67	Au	76	316.13	1.87	Bu
6	38.20	0.16	Au	77	317.83	2.73	Au
7	41.84	0.57	Bu	78	317.90	7.35	Bu
10	46.98	2.70	Bu	83	382.36	103.16	Bu
13	51.03	0.10	Au	84	383.55	3.54	Au
16	57.05	0.08	Au	87	397.68	62.49	Bu
18	60.61	2.67	Bu	88	398.15	1.87	Au
20	66.32	1.40	Bu	89	406.86	1.08	Au
22	67.42	1.09	Au	91	408.84	10.35	Bu
25	74.58	1.23	Au	93	412.71	6.06	Bu
27	77.67	3.25	Bu	94	414.12	0.35	Au
29	82.61	0.20	Au	97	424.13	0.37	Bu
31	87.48	1.00	Bu	99	425.16	1.66	Au
33	99.62	0.05	Au	101	446.10	2.51	Bu
36	105.82	8.36	Au	103	447.59	0.00	Au
38	106.20	3.32	Bu	107	482.53	1.56	Au
40	110.73	3.60	Bu	108	483.57	24.91	Bu
43	115.31	9.70	Au	109	531.53	219.91	Bu
44	115.55	0.74	Bu	110	532.62	2.80	Au
47	119.11	11.40	Bu	115	592.07	2.07	Au
48	120.57	3.32	Bu	116	592.73	108.53	Bu
49	123.35	0.93	Au	118	612.87	0.13	Au
50	127.63	18.15	Au	119	613.53	18.02	Bu
53	137.09	96.61	Au	121	613.93	15.98	Bu
56	144.59	6.80	Bu	122	614.30	10.62	Au
57	147.24	2.19	Bu	126	643.18	31.57	Au
58	151.95	2.72	Au	127	643.57	26.75	Bu
63	187.72	11.19	Bu	129	683.11	93.81	Au
64	189.85	0.02	Au	131	685.46	588.42	Bu
65	221.79	0.69	Au	132	690.21	130.91	Bu
67	224.31	1.05	Bu	136	693.26	0.02	Au
69	273.74	19.49	Bu	137	710.67	2.48	Au
71	275.89	12.14	Au	138	710.94	25.06	Bu
74	314.01	0.15	Au	141	723.27	143.66	Au

142	725.58	231.30	Bu	227	1101.27	241.56	Bu
145	750.18	51.87	Bu	228	1104.58	3.13	Au
146	753.79	24.24	Au	231	1108.94	298.63	Bu
149	757.74	12.69	Au	232	1109.08	90.57	Au
150	760.09	148.95	Bu	233	1112.09	34.54	Au
153	820.85	1.31	Au	234	1112.37	1.65	Bu
154	825.19	6.43	Bu	237	1145.04	12.04	Bu
158	831.20	19.84	Bu	240	1146.43	0.70	Au
160	831.40	3.81	Au	241	1154.35	4.31	Bu
163	834.16	9.67	Bu	242	1154.36	0.78	Au
164	844.18	2.02	Au	247	1172.37	5.24	Bu
167	889.32	0.60	Au	248	1172.93	1.89	Au
168	889.57	8.36	Bu	251	1179.02	2.26	Au
169	895.98	14.75	Au	252	1181.36	27.37	Bu
171	900.33	32.08	Bu	253	1214.65	197.61	Bu
174	942.05	148.93	Bu	255	1216.20	18.30	Au
176	942.42	1.10	Au	259	1275.13	125.87	Bu
179	945.03	0.51	Au	260	1276.24	27.73	Au
181	947.09	6.71	Bu	262	1297.68	134.07	Bu
183	947.75	17.08	Bu	264	1299.82	18.84	Au
184	950.01	0.94	Au	266	1302.88	517.21	Bu
187	966.74	1.78	Bu	268	1303.95	38.97	Au
188	967.30	13.30	Au	269	1307.56	4.33	Au
190	982.70	2.54	Au	272	1312.17	42.60	Bu
192	983.98	3.54	Bu	275	1342.74	382.78	Bu
194	993.49	5.75	Bu	276	1343.55	46.98	Au
195	994.02	3.60	Au	277	1346.17	5.26	Au
197	995.62	2.59	Bu	278	1346.23	40.96	Bu
198	997.07	0.21	Au	282	1352.50	1561.33	Bu
201	1021.40	17.12	Bu	284	1357.22	198.79	Au
203	1023.27	9.73	Au	287	1396.96	170.43	Bu
206	1026.82	61.99	Bu	288	1398.65	0.41	Au
208	1029.57	0.05	Au	289	1411.80	0.00	Au
209	1037.82	33.42	Au	290	1412.42	556.53	Bu
211	1039.00	3.99	Bu	293	1425.12	16.51	Bu
215	1076.87	8.30	Bu	294	1425.20	3.79	Au
216	1077.64	24.82	Au	298	1437.25	178.55	Au
217	1079.24	58.48	Au	299	1438.95	45.01	Bu
219	1081.86	47.39	Bu	302	1443.83	17.43	Bu
221	1093.43	2.73	Bu	303	1443.85	11.10	Au
222	1093.71	52.01	Au	305	1445.65	7.78	Bu

306	1445.83	83.19	Au	390	3126.05	23.12	Au
309	1456.23	0.08	Au	392	3126.47	31.93	Bu
311	1459.47	26.32	Bu	395	3132.17	49.97	Bu
315	1466.60	330.41	Bu	396	3132.25	0.44	Au
316	1467.11	26.26	Au	397	3137.15	30.80	Bu
317	1486.43	279.32	Bu	398	3137.34	1.37	Au
319	1488.67	115.56	Au	401	3138.28	1.29	Au
322	1490.71	796.44	Bu	402	3138.33	7.84	Bu
324	1491.75	8.37	Au	405	3148.16	1.16	Au
327	1579.88	2.55	Au	407	3148.48	3.56	Bu
328	1580.44	1421.80	Bu				
329	1581.55	32.87	Bu				
332	1582.88	11.45	Au				
333	1587.39	1145.97	Bu				
335	1592.95	46.07	Au				
339	1597.44	0.85	Bu				
340	1597.65	31.23	Au				
342	1631.78	37.48	Au				
343	1631.93	3237.41	Bu				
346	2967.15	307.97	Bu				
347	2967.48	1.05	Au				
349	2968.69	183.44	Bu				
350	2968.79	9.25	Au				
353	3030.91	108.38	Au				
355	3031.01	16.61	Bu				
357	3044.16	110.06	Au				
359	3044.28	14.41	Bu				
363	3090.03	23.21	Au				
364	3090.05	58.87	Bu				
365	3099.36	74.63	Bu				
367	3099.58	65.20	Au				
370	3101.63	3.63	Au				
372	3102.15	445.39	Bu				
373	3105.38	15.83	Bu				
376	3105.74	36.32	Au				
377	3109.56	3.71	Au				
378	3109.66	13.95	Bu				
383	3111.02	105.73	Bu				
384	3111.14	25.25	Au				
385	3123.94	23.74	Bu				
386	3124.12	46.62	Au				

Table S24. ss-DFT Raman-active modes with frequencies (cm^{-1}), intensities (arb. units), and mode symmetries for DMDPU full optimization. Intensities are scaled to a maximum of 1000.

Mode Label (ν)	Frequency (cm^{-1})	Intensity (arb. units)	Mode Symm.	Mode Label (ν)	Frequency (cm^{-1})	Intensity (arb. units)	Mode Symm.
4	34.71	Ag	235.73	70	275.45	Ag	29.94
8	43.37	Ag	169.95	72	279.76	Bg	1.42
9	44.23	Bg	110.53	73	313.21	Bg	27.14
11	47.42	Bg	28.41	75	314.03	Ag	78.49
12	48.12	Bg	16.78	79	319.26	Ag	117.53
14	51.19	Ag	176.05	80	320.23	36.38	Bg
15	52.69	Ag	228.36	81	379.49	143.84	Ag
17	58.14	Bg	75.29	82	381.60	0.03	Bg
19	60.97	Ag	239.80	85	394.70	379.82	Ag
21	66.57	Ag	172.06	86	396.42	11.55	Bg
23	69.06	Bg	16.06	90	407.09	4.02	Bg
24	71.47	Bg	36.36	92	409.27	6.40	Ag
26	76.12	Ag	700.10	95	414.25	22.96	Bg
28	77.95	Bg	67.17	96	414.61	8.35	Ag
30	85.83	Bg	241.54	98	424.81	32.09	Ag
32	88.53	Ag	358.31	100	425.86	1.33	Bg
34	101.71	Bg	619.81	102	447.45	156.18	Ag
35	102.69	Ag	578.97	104	448.14	4.39	Bg
37	106.11	Bg	128.96	105	479.08	69.99	Bg
39	109.32	Ag	509.27	106	481.73	17.53	Ag
41	112.13	Bg	282.81	111	532.95	1.95	Ag
42	113.70	Ag	410.59	112	534.24	24.86	Bg
45	118.37	Ag	705.01	113	591.00	15.18	Bg
46	119.08	Bg	1000.00	114	591.15	53.64	Ag
51	132.66	Bg	31.69	117	612.60	75.83	Ag
52	137.02	Ag	198.78	120	613.70	65.93	Bg
54	142.32	Ag	213.29	123	615.90	4.34	Bg
55	143.23	Bg	223.91	124	616.04	149.75	Ag
59	162.12	Ag	93.55	125	642.61	1.82	Ag
60	163.68	Bg	172.17	128	644.13	26.41	Bg
61	179.61	Bg	38.26	130	684.99	9.62	Ag
62	181.25	Ag	135.52	133	690.38	0.74	Bg
66	223.37	Bg	195.72	134	691.32	8.07	Ag
68	226.22	Ag	155.08	135	692.04	5.75	Bg
139	711.28	29.64	Bg	220	1084.31	6.85	Ag
140	712.10	437.84	Ag	223	1094.24	237.17	Ag

143	727.37	7.37	Bg	224	1095.25	11.96	Bg
144	728.29	11.90	Ag	225	1098.47	124.13	Ag
147	755.34	40.15	Ag	226	1100.06	2.12	Bg
148	756.34	1.21	Bg	229	1106.46	81.71	Ag
151	764.34	5.99	Bg	230	1108.36	1.44	Bg
152	765.38	37.75	Ag	235	1112.42	11.67	Bg
155	825.99	13.64	Bg	236	1113.17	94.93	Ag
156	828.57	19.34	Ag	238	1145.80	17.22	Bg
157	829.32	12.68	Bg	239	1145.88	41.57	Ag
159	831.26	19.29	Ag	243	1154.41	41.59	Ag
161	833.66	1.67	Bg	244	1154.56	9.61	Bg
162	833.84	16.56	Ag	245	1170.21	16.29	Ag
165	887.93	11.53	Ag	246	1172.16	4.02	Bg
166	888.31	5.37	Bg	249	1175.65	60.61	Ag
170	896.66	22.40	Ag	250	1176.31	45.97	Bg
172	902.15	0.71	Bg	254	1216.00	42.87	Ag
173	941.97	71.33	Ag	256	1217.81	7.70	Bg
175	942.07	11.36	Bg	257	1272.87	140.23	Ag
177	944.36	19.18	Ag	258	1274.59	113.93	Bg
178	944.44	5.23	Bg	261	1296.78	20.05	Ag
180	945.87	0.64	Bg	263	1299.29	28.94	Bg
182	947.24	11.66	Ag	265	1301.92	35.45	Bg
185	966.48	0.15	Bg	267	1303.32	784.23	Ag
186	966.74	19.89	Ag	270	1311.17	200.99	Ag
189	982.68	225.38	Ag	271	1311.86	17.16	Bg
191	983.29	3.41	Bg	273	1341.06	29.99	Ag
193	992.80	5.99	Bg	274	1342.60	0.34	Bg
196	994.57	293.68	Ag	279	1346.38	16.79	Ag
199	1000.23	752.85	Ag	280	1346.81	2.32	Bg
200	1000.43	19.84	Bg	281	1352.22	74.39	Ag
202	1022.25	42.18	Ag	283	1355.19	15.43	Bg
204	1024.20	2.70	Bg	285	1391.39	16.35	Ag
205	1026.17	1.90	Bg	286	1393.60	10.35	Bg
207	1028.27	738.94	Ag	291	1412.45	16.41	Bg
210	1037.94	28.75	Bg	292	1416.23	18.68	Ag
212	1039.30	24.51	Ag	295	1425.78	50.06	Bg
213	1074.80	16.86	Ag	296	1426.04	58.27	Ag
214	1075.93	3.71	Bg	297	1437.25	76.68	Ag
218	1079.49	15.30	Bg	300	1439.20	19.07	Bg
301	1443.38	11.37	Ag	382	3110.90	8.88	Bg
304	1444.46	15.16	Bg	387	3124.89	38.02	Bg

307	1446.14	19.99	Ag	388	3125.09	72.92	Ag
308	1446.26	0.78	Bg	389	3125.97	31.42	Bg
310	1458.31	79.28	Ag	391	3126.06	77.52	Ag
312	1460.80	2.40	Bg	393	3131.25	451.79	Ag
313	1464.07	39.67	Ag	394	3131.61	76.97	Bg
314	1465.36	2.74	Bg	399	3137.64	27.36	Bg
318	1486.51	7.33	Bg	400	3137.65	106.82	Ag
320	1490.27	29.71	Ag	403	3138.88	75.12	Bg
321	1490.47	38.82	Ag	404	3139.10	350.62	Ag
323	1490.73	10.07	Bg	406	3148.45	77.46	Bg
325	1578.33	26.95	Ag	408	3148.64	478.33	Ag
326	1578.92	2.05	Bg				
330	1582.05	21.59	Ag				
331	1582.27	43.03	Bg				
334	1590.43	289.70	Ag				
336	1594.05	130.88	Bg				
337	1595.58	30.73	Ag				
338	1596.56	266.82	Bg				
341	1630.74	7.27	Bg				
344	1633.22	246.99	Ag				
345	2966.99	169.00	Ag				
348	2967.71	24.37	Bg				
351	2968.85	16.16	Bg				
352	2969.13	800.08	Ag				
354	3030.91	72.35	Ag				
356	3031.13	106.15	Bg				
358	3044.22	66.35	Ag				
360	3044.29	111.66	Bg				
361	3089.96	24.88	Bg				
362	3089.99	97.59	Ag				
366	3099.44	98.25	Ag				
368	3099.79	112.30	Bg				
369	3101.39	32.90	Bg				
371	3101.91	118.54	Ag				
374	3105.39	80.93	Ag				
375	3105.74	88.55	Bg				
379	3109.67	145.10	Ag				
380	3109.84	109.48	Bg				
381	3110.78	332.19	Ag				

Table S25. Linear Regression for Raman active peak at 98.8 cm^{-1}.

Linear Regression for Peak at 98.8 cm^{-1}	
slope (m)	28.12
y-intercept (b)	0.91
Standard error	1.89
Standard error of y-intercept (b)	1.10
Standard error in y	1.26
R squared	0.99
F value	222.50
Degrees of Freedom	3
Sum of squares of regression	351.85
Sum of squares of residuals	4.74
Limit of Detection (LOD)	0.13
Limit of Quantitation (LOQ)	0.39

Table S26. Linear Regression for Raman active peak at 111.7 cm^{-1}.

Linear Regression for Peak at 111.7 cm^{-1}	
slope (m)	-21.16
y-intercept (b)	21.00
Standard error	3.71
Standard error of y-intercept (b)	2.16
Standard error in y	2.47
R squared	0.92
F value	32.58
Degrees of Freedom	3
Sum of squares of regression	199.20
Sum of squares of residuals	18.34
Limit of Detection (LOD)	0.34
Limit of Quantitation (LOQ)	1.02

Table S27. Linear Regression for Raman active peak at 247.6 cm^{-1}.

Linear Regression for Peak at 247.6 cm^{-1}	
slope (m)	24.32
y-intercept (b)	1.09
Standard error	2.17
Standard error of y-intercept (b)	1.26
Standard error in y	1.45
R squared	0.98
F value	125.70
Degrees of Freedom	3
Sum of squares of regression	263.21
Sum of squares of residuals	6.28
Limit of Detection (LOD)	0.17
Limit of Quantitation (LOQ)	0.52

Table S28. Linear Regression for Raman active peak at 260.1 cm^{-1}.

Linear Regression for Peak at 260.1 cm^{-1}	
slope (m)	18.54
y-intercept (b)	-2.08
Standard error	1.24
Standard error of y-intercept (b)	0.72
Standard error in y	0.83
R squared	0.99
F value	224.28
Degrees of Freedom	3
Sum of squares of regression	152.89
Sum of squares of residuals	2.05
Limit of Detection (LOD)	0.13
Limit of Quantitation (LOQ)	0.39

Table S29. Linear Regression for Raman active peak at 297.6 cm^{-1}.

Linear Regression for Peak at 297.6 cm^{-1}	
slope (m)	32.41
y-intercept (b)	1.04
Standard error	2.92
Standard error of y-intercept (b)	1.70
Standard error in y	1.95
R squared	0.98
F value	123.60
Degrees of Freedom	3
Sum of squares of regression	467.56
Sum of squares of residuals	11.35
Limit of Detection (LOD)	0.17
Limit of Quantitation (LOQ)	0.52

Table S30. THz pellet specifications for DEDPU and DMDPU. Pathlength is represented in millimeters, masses are represented in milligrams and concentration is represented as a percentage of mass of OGSR / total pellet mass.

OGSR	Pathlength	Mass of OGSR	Mass of PTFE	Final OGSR Mass Percentage
**DEDPU**	3.15	31.15	968.85	3.12
**DMDPU**	3.04	29.09	970.91	2.91

Table S31. Signal-to-noise values for LFRS and THz data sets of each OGSR at 295 K and 78 K.

	DEDPU		DMDPU	
Temperature	LFRS	THz	LFRS	THz
295 K	232	6	34	2
78 K	452	29	59	16

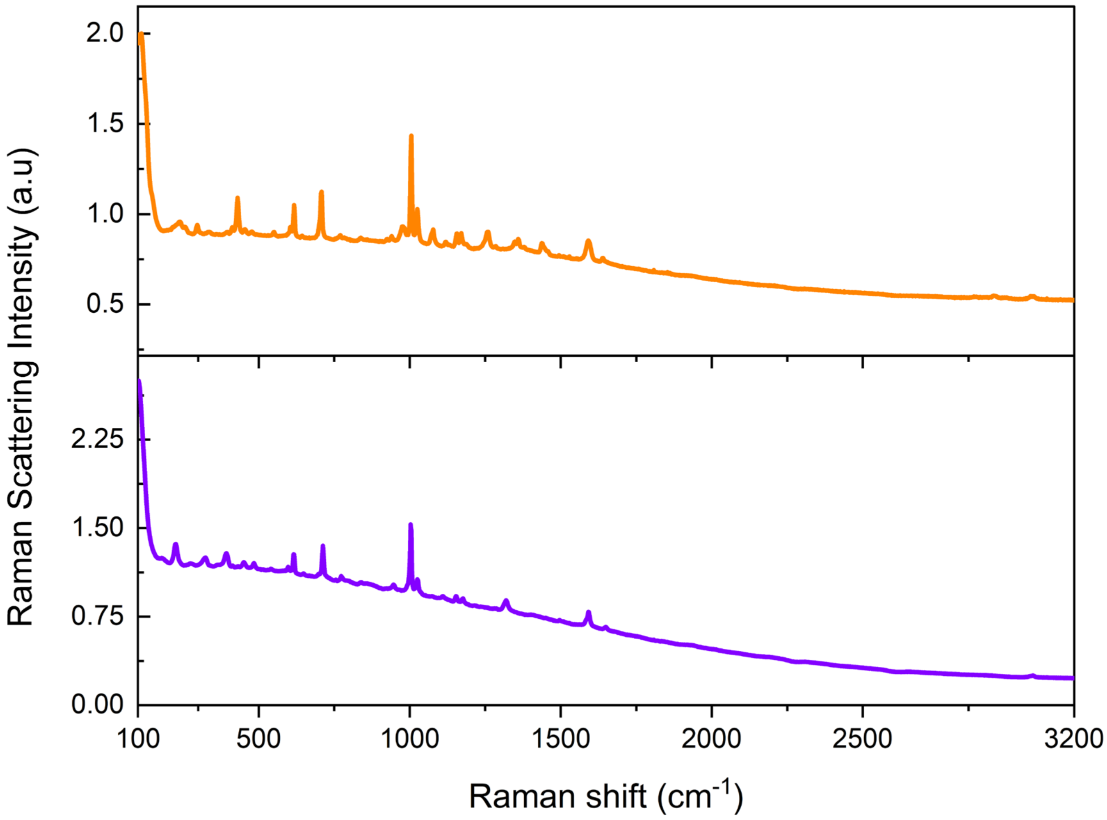

Figure S1. Baseline uncorrected and non-normalized high-frequency Raman data for DEDPU (orange) and DMDPU (purple) taken at 295 K demonstrating impact of fluorescence.

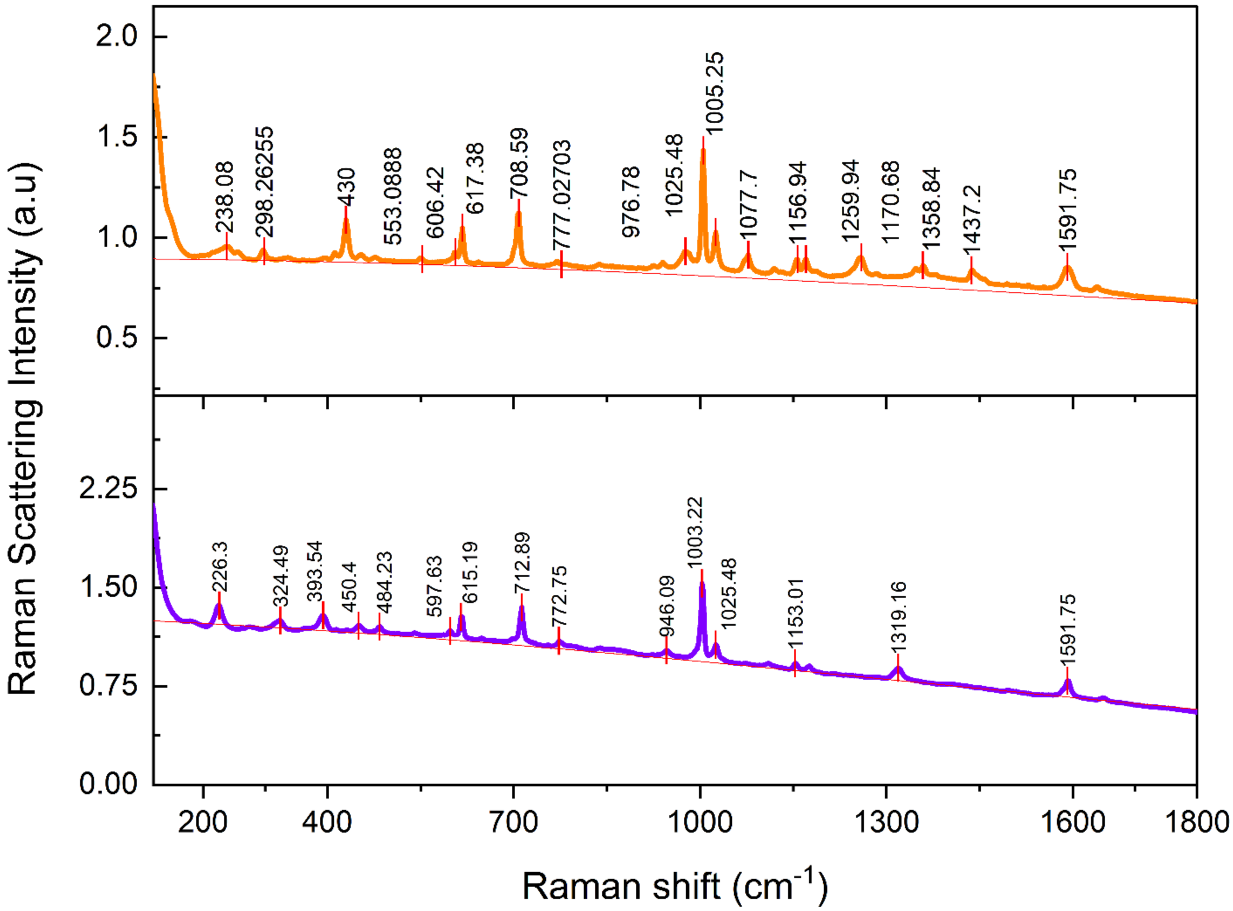

Figure S2. Uncorrected high-frequency Raman spectra of DEDPU (orange) and DMDPU (purple) with peak centers at 295 K from 100-1800 cm^{-1}. Some peak centers are offset to avoid overlap.

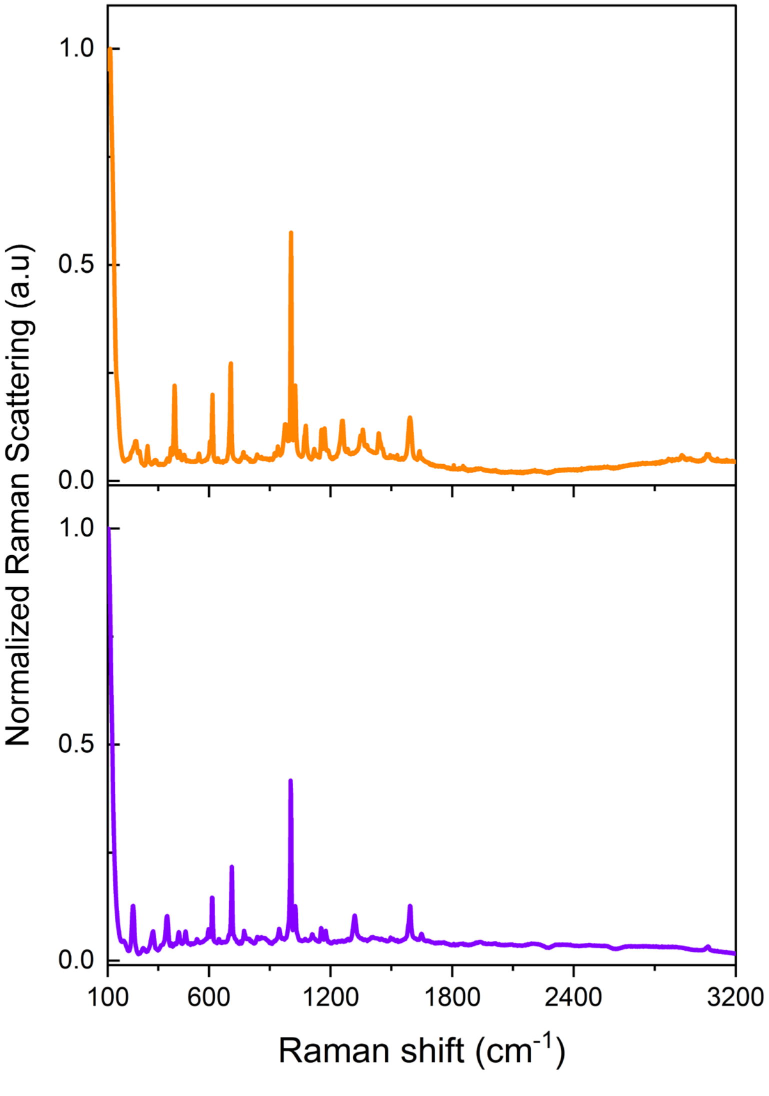

Figure S3. Baseline Corrected and normalized HFRS (full spectrum) of DEDPU (orange) and DMDPU (purple) at 295 K. Spectra were normalized to 1.

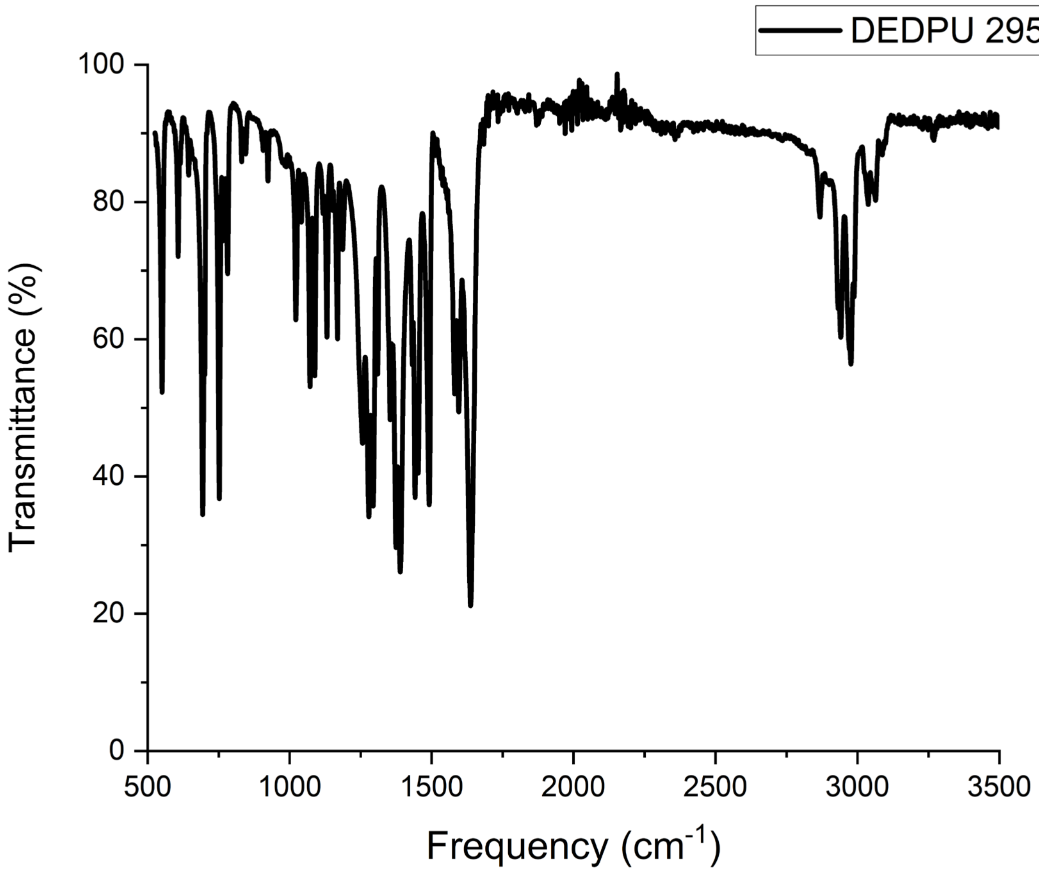

Figure S4. IR spectrum of DEDPU at 295 K

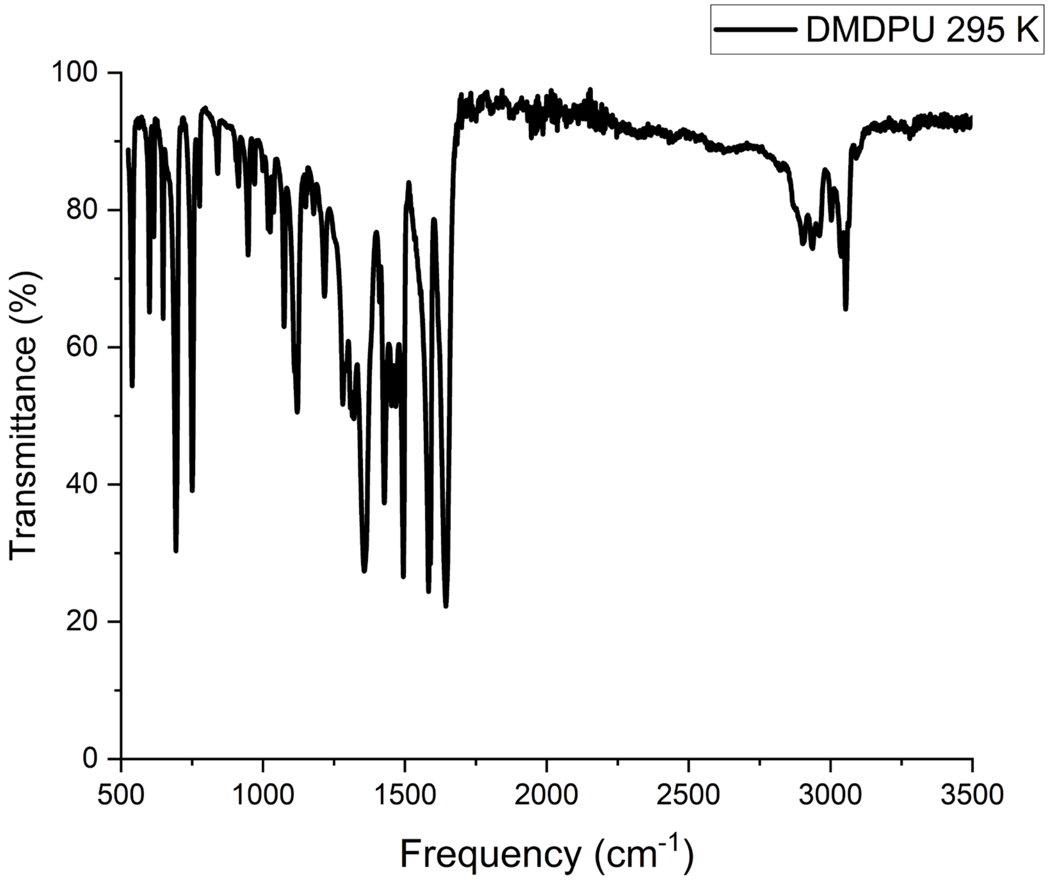

Figure S5. IR spectrum of DMDPU at 295 K

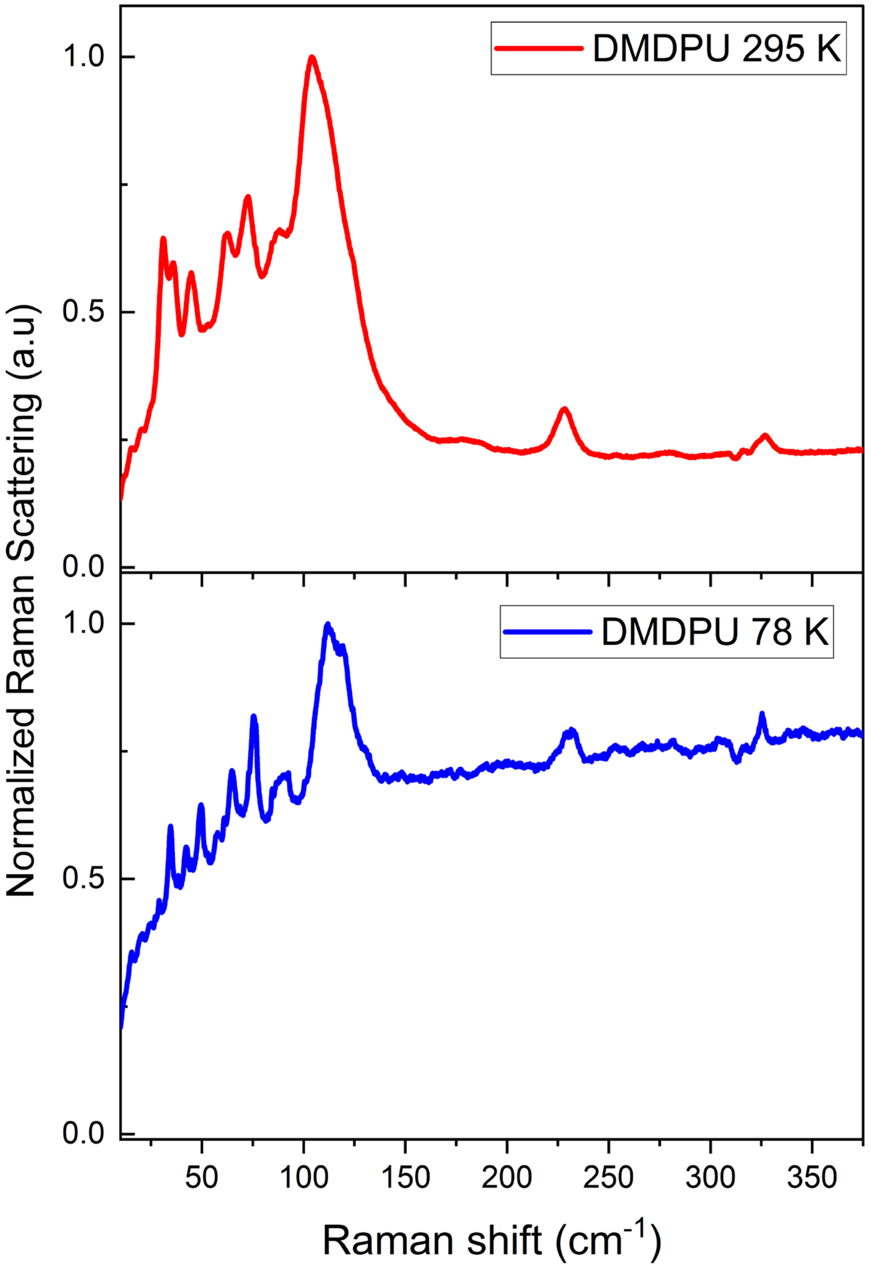

Figure S6. Uncorrected LFRS of DMDPU at 295 K (red) and 78 K (blue). All spectra normalized to 1.

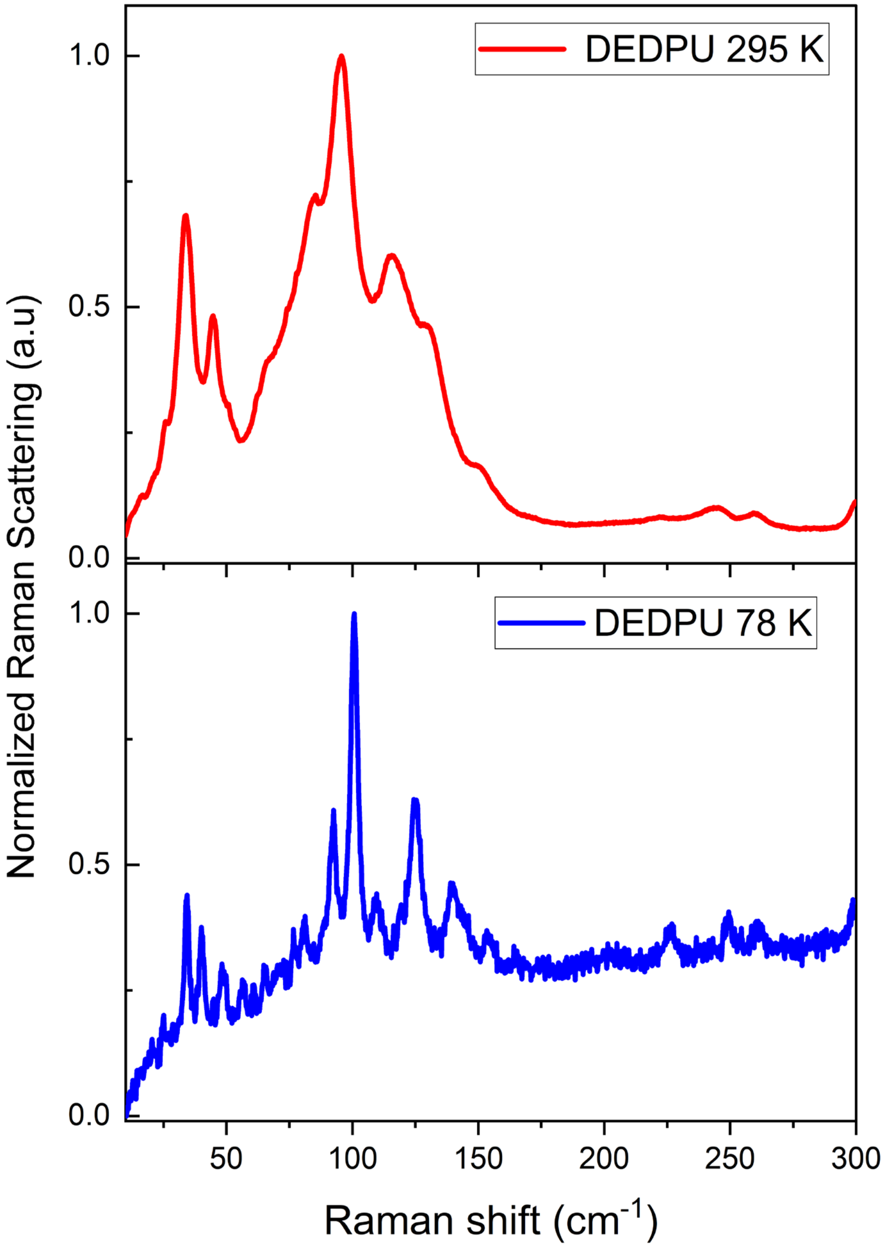

Figure S7. Uncorrected LFRS of DEDPU at 295 K (red) and 78 K (blue). All spectra normalized to 1.

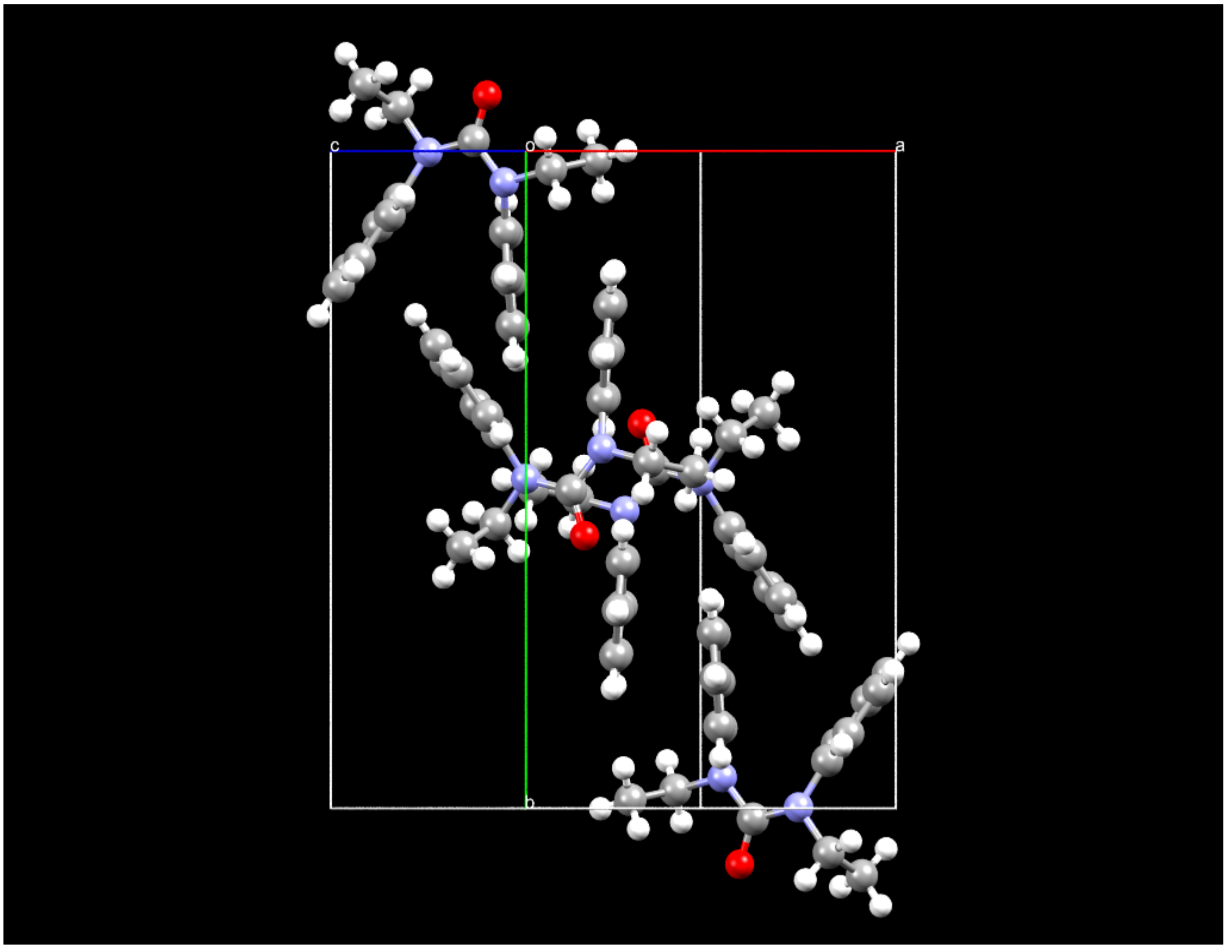

Figure S8. Crystallographic unit cell of DEDPU. REF Code: UNUKUK, $P2_1/c$ Z=4, Z'=1

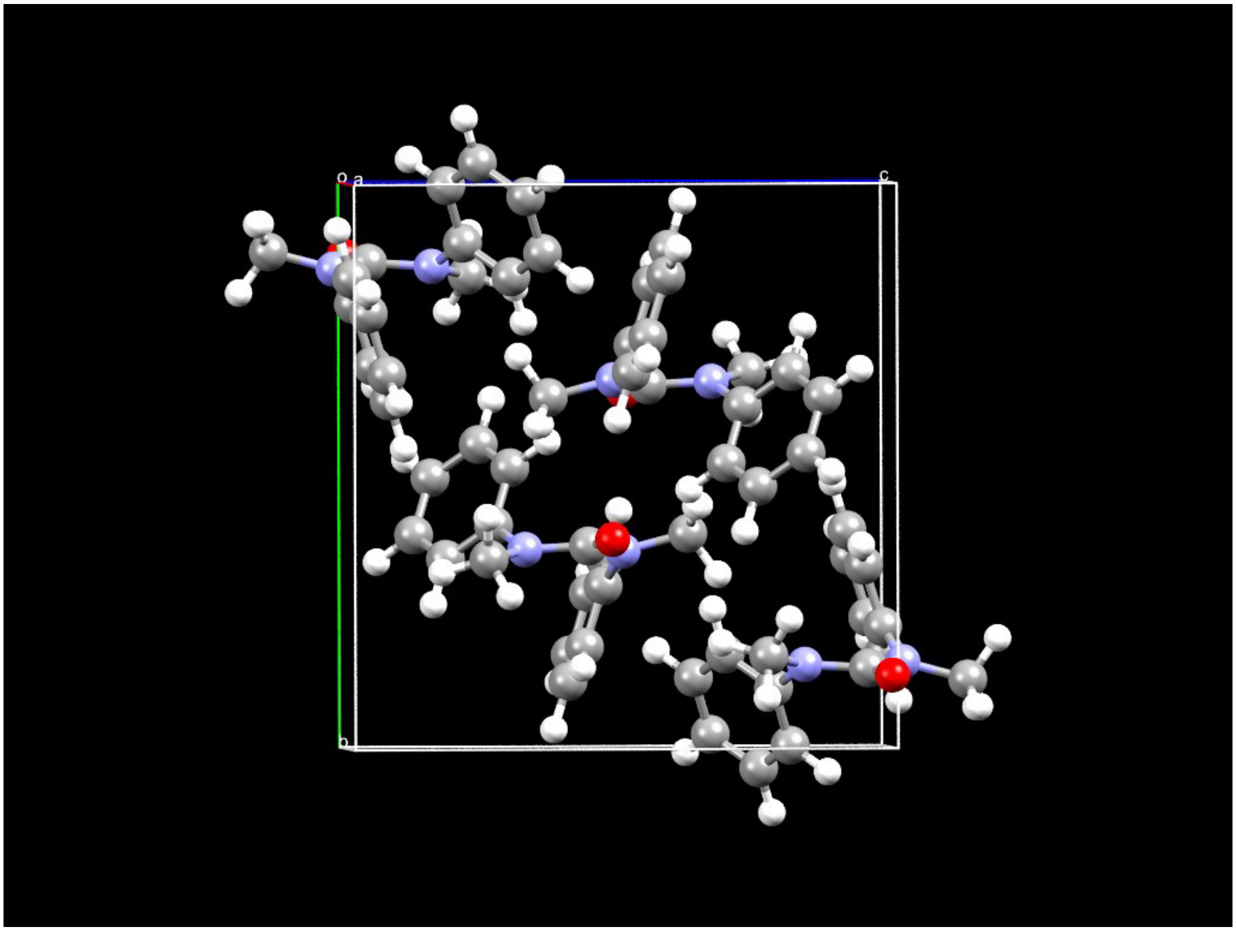

Figure S9. Crystallographic unit cell of DMDPU. REF code: CEJDUT, $P2_1/n$ Z=4, Z'=1

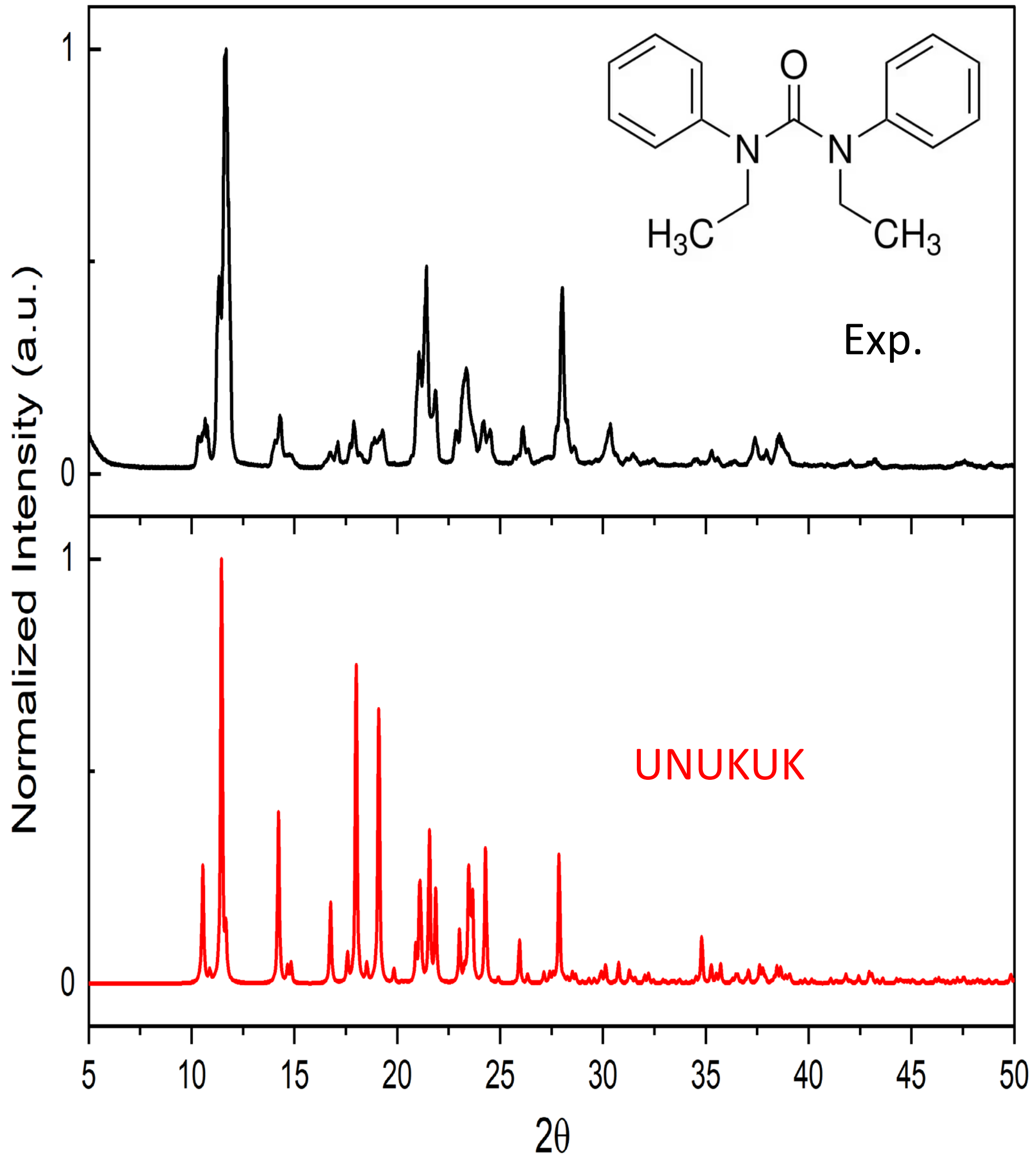

Figure S10. Experimental powder X-Ray pattern for DEDPU (black) compared to predicted pattern from the CSD (red). Data has been normalized to 1.

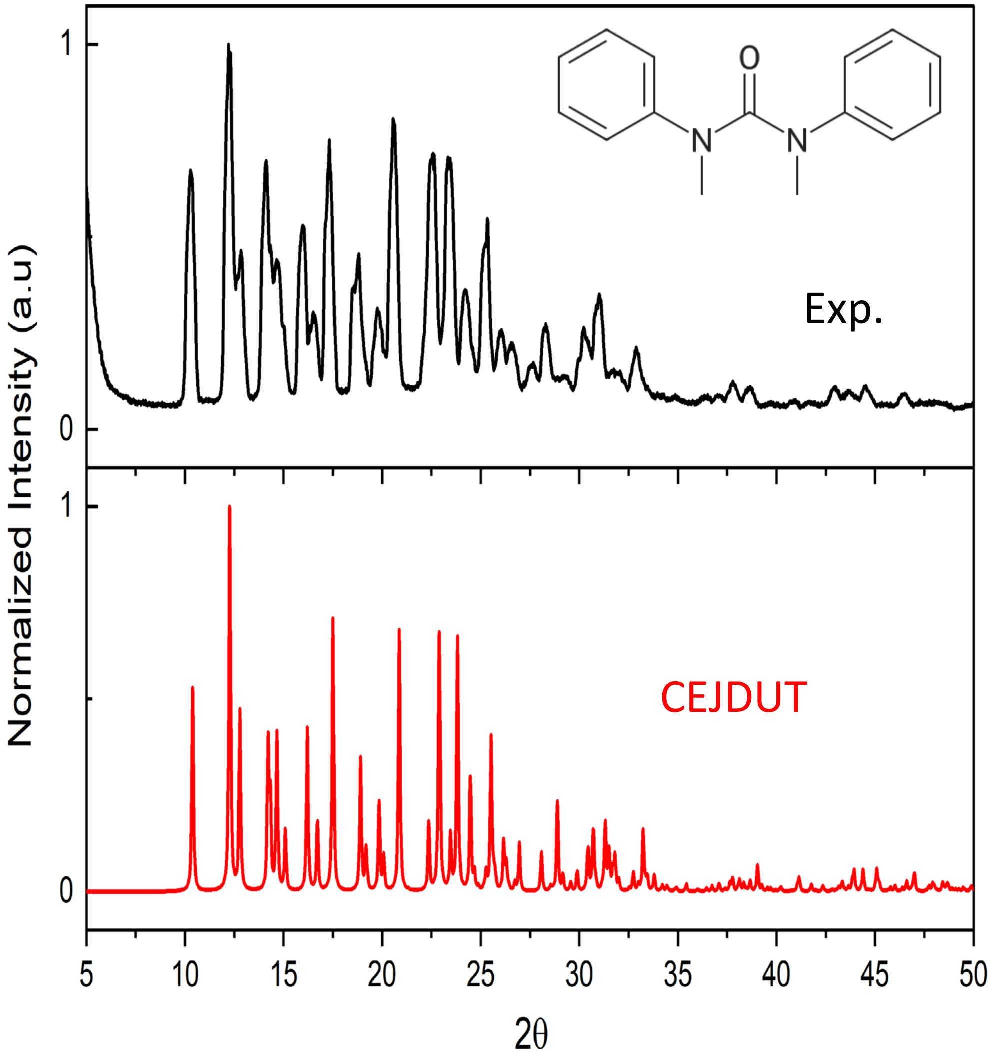

Figure S11. Experimental powder X-ray pattern for DMDPU (black) compared to predicted pattern from the CSD (red). Data has been normalized to 1.

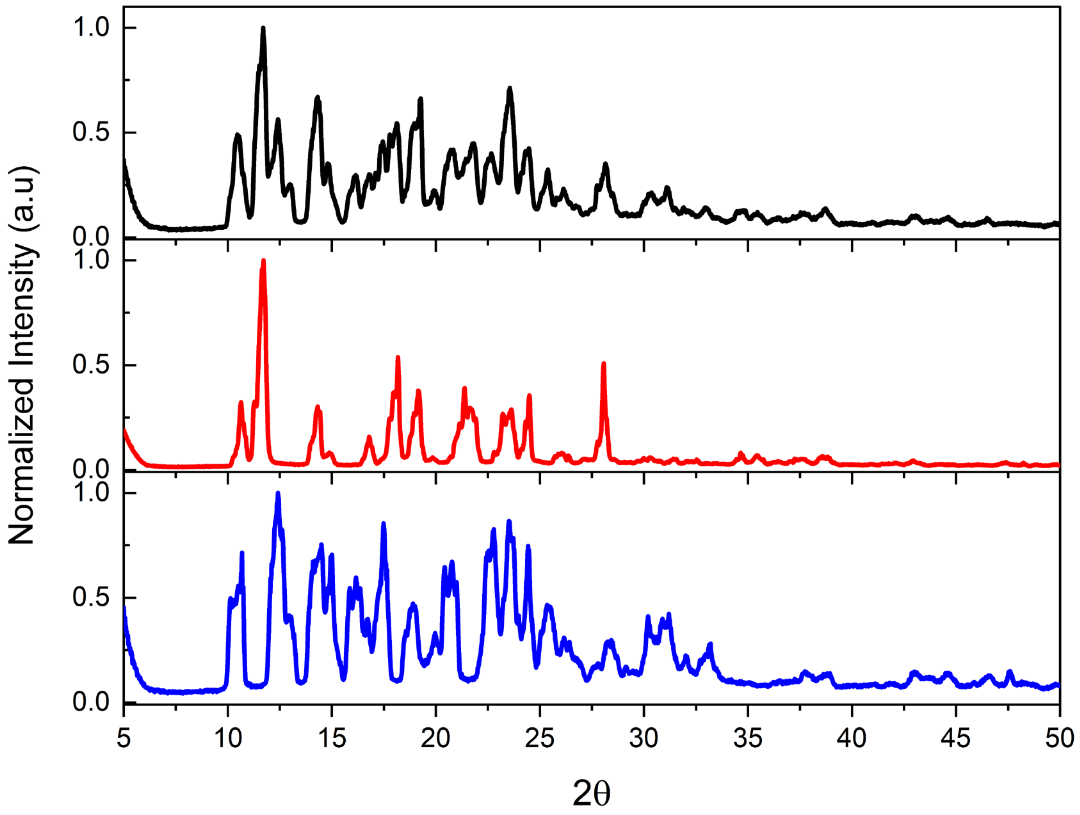

Figure S12. Comparison of PXRD data of equimolar DEDPU:DMDPU mixture (black) to pure DEDPU (red) and pure DMDPU (blue). Data taken at 295 K.

Peak Analysis

Data Set:[Book1]Sheet1!D Date:4/30/2025

BaseLine:Poly4

Chi^2=7.40472E-04 Adj. R-Square=9.85642E-01 # of Data Points=891

SS=6.26439E-01 Degrees of Freedom=846

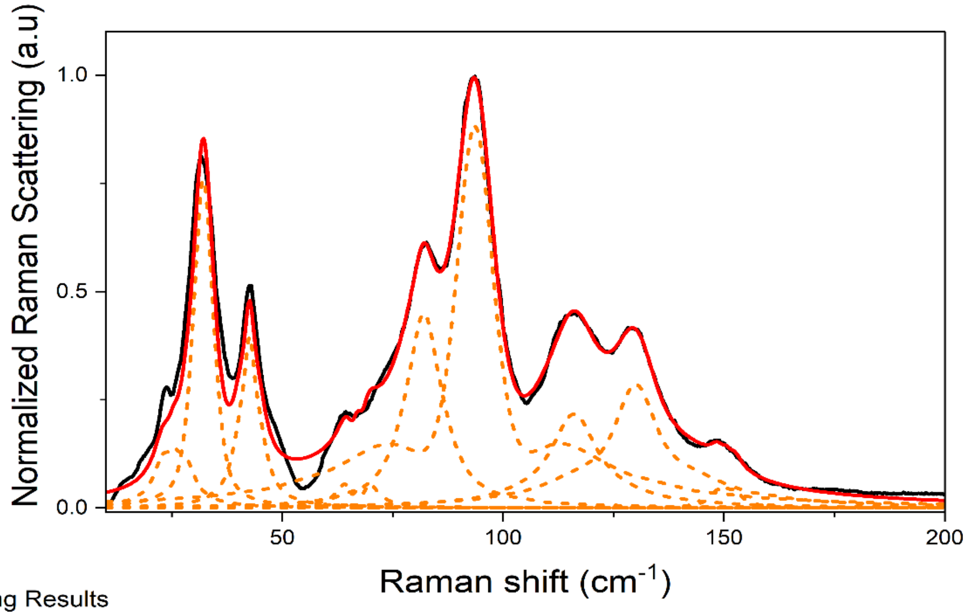

Fitting Results

Peak Index	Peak Type	Area Intg	FWHM	Max Height	Center Grvty	Area IntgP
1	PsdVoigt2	1.73216	9.52407	0.14004	25.43919	3.49299
2	PsdVoigt2	5.31661	5.3817	0.7582	32.13853	10.72121
3	PsdVoigt2	3.04267	5.33887	0.39615	42.59853	6.13569
4	PsdVoigt2	0.76182	11.00129	0.05503	66.99324	1.53624
5	PsdVoigt2	6.60409	10.06156	0.45067	82.04588	13.31748
6	PsdVoigt2	19.4397	10.15067	0.88141	93.5764	39.20113
7	PsdVoigt2	4.78148	13.69639	0.21669	116.0558	9.6421
8	PsdVoigt2	7.42323	13.88366	0.28533	129.99983	14.96932
9	PsdVoigt2	0.48788	7.74306	0.04582	150.50531	0.98384

Figure S13. Pseudo-Voigt Line-shape analysis fit 295 K Raman spectrum for pure DEDPU from 10-200 cm^{-1}. Experimental traces shown in black, fit peaks shown in dashed orange and fitted trace shown in red. Spectrum normalized to 1.

Peak Analysis

Data Set:[Book1]Sheet1!F		Date:4/30/2025
BaseLine:Poly4		
Chi^2=8.38828E-06	Adj. R-Square=9.77004E-01	# of Data Points=495
SS=3.94249E-03	Degrees of Freedom=470	

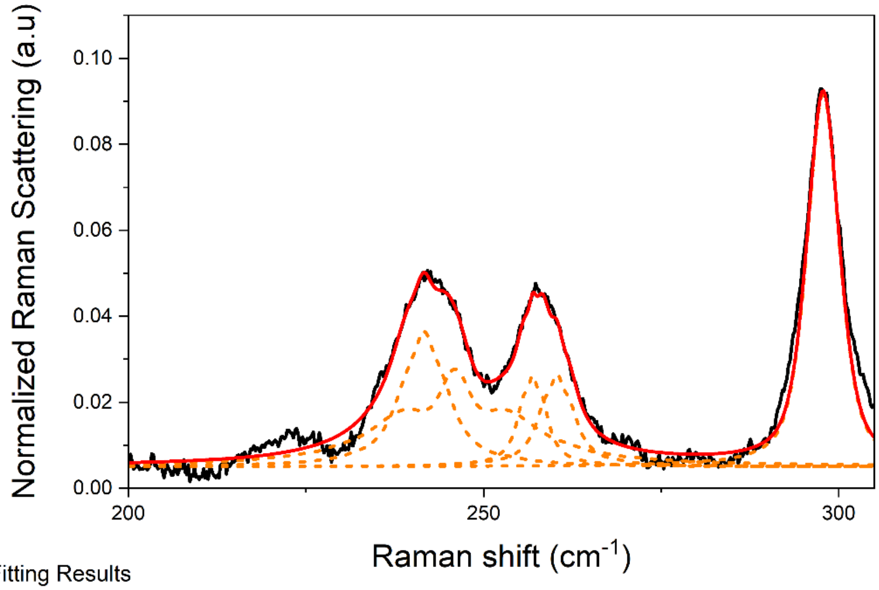

Fitting Results

Peak Index	Peak Type	Area Intg	FWHM	Max Height	Center Grvty	Area IntgP
1	PsdVoigt2	0.50241	18.98284	0.02271	245.97558	29.48623
2	PsdVoigt2	0.12715	4.43152	0.02131	256.91608	7.46252
3	PsdVoigt2	0.58735	5.43814	0.0867	297.78706	34.47087
4	PsdVoigt2	0.32491	7.61634	0.03142	241.61701	19.06859
5	PsdVoigt2	0.16207	5.57008	0.02159	260.32282	9.51179

Figure S14. Pseudo-Voigt Line-shape analysis fit 295 K Raman spectrum for pure DEDPU from 200-305 cm^{-1}. Experimental traces shown in black, fit peaks shown in dashed orange and fitted trace shown in red. Spectrum normalized to 1.

Peak Analysis

Data Set:[Book1]Sheet1!D   Date:4/30/2025

BaseLine:Poly4

Chi^2=5.55277E-04   Adj. R-Square=9.72339E-01   # of Data Points=891

SS=4.47554E-01   Degrees of Freedom=806

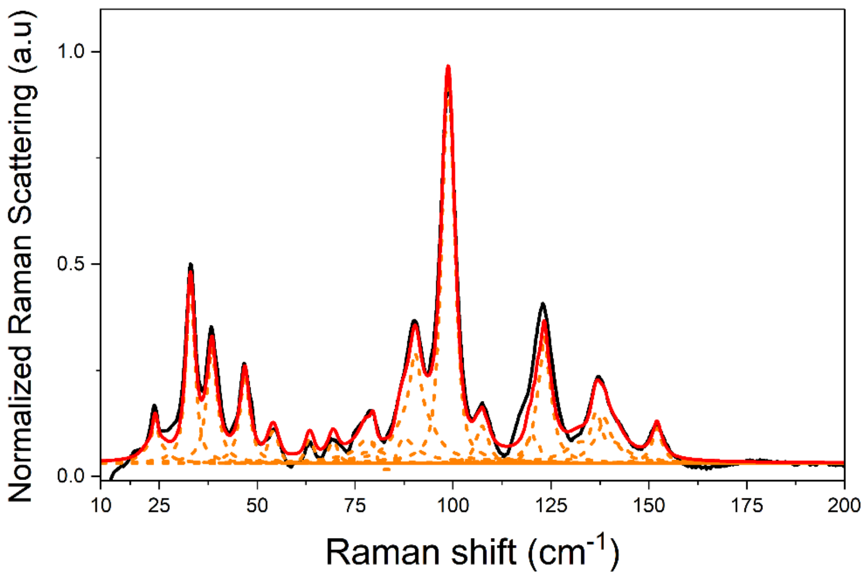

Peak Index	Peak Type	Area Intg	FWHM	Max Height	Center Grvty	Area IntgP
1	PsdVoigt2	0.52343	2.59984	0.10495	23.92143	2.78172
2	PsdVoigt2	1.66363	2.70731	0.42262	32.93681	8.84117
3	PsdVoigt2	1.28216	3.24153	0.26883	38.42717	6.81389
4	PsdVoigt2	0.95765	3.01451	0.21141	46.84194	5.08932
5	PsdVoigt2	0.3115	3.2648	0.07604	54.08928	1.65544
6	PsdVoigt2	0.99683	5.70768	0.05886	82.94979	5.29757
7	PsdVoigt2	2.44809	5.53282	0.25716	90.35978	13.01014
8	PsdVoigt2	4.68431	4.01896	0.88501	98.78896	24.89431
9	PsdVoigt2	0.47996	3.94078	0.08778	107.48036	2.55071
10	PsdVoigt2	1.68564	3.94376	0.29663	123.33193	8.95819
11	PsdVoigt2	1.38217	4.2615	0.12152	136.61198	7.34539
12	PsdVoigt2	0.40506	3.17177	0.0837	152.12946	2.15264
13	PsdVoigt2	0.21651	2.64589	0.05831	63.39587	1.15065
14	PsdVoigt2	0.21186	2.83908	0.05292	69.3903	1.12589
15	PsdVoigt2	0.34951	3.91836	0.06592	120.39939	1.85742
16	PsdVoigt2	0.32797	5.37695	0.04784	79.66216	1.74297
17	PsdVoigt2	0.89052	5.3559	0.10576	138.67139	4.73259

Figure S15. Pseudo-Voigt Line-shape analysis fit 78 K Raman spectrum for pure DEDPU from 10-200 cm^{-1}. Experimental traces shown in black, fit peaks shown in dashed orange and fitted trace shown in red. Spectrum normalized to 1.

Peak Analysis

Data Set:[Book1]Sheet1!F		Date:4/30/2025
BaseLine:Poly4		
Chi^2=1.30660E-05	Adj. R-Square=9.72701E-01	# of Data Points=495
SS=6.07568E-03	Degrees of Freedom=465	

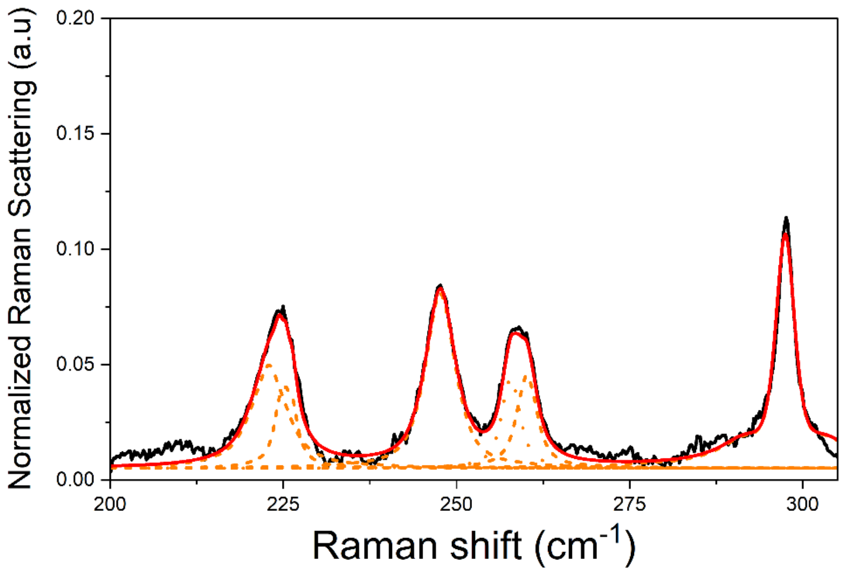

Peak Index	Peak Type	Area Intg	FWHM	Max Height	Center Grvty	Area IntgP
1	PsdVoigt2	0.38157	6.14269	0.04462	222.9262	18.3173
2	PsdVoigt2	0.16985	3.51879	0.03518	225.37924	8.15364
3	PsdVoigt2	0.53858	5.13592	0.07556	247.66988	25.85445
4	PsdVoigt2	0.1782	3.1855	0.03924	257.84381	8.55468
5	PsdVoigt2	0.20268	3.50556	0.04036	260.18725	9.72937
6	PsdVoigt2	0.61224	3.42097	0.1012	297.47136	29.39055

Figure S16. Pseudo-Voigt Line-shape analysis fit 78 K Raman spectrum for pure DEDPU from 200-305 cm^{-1}. Experimental traces shown in black, fit peaks shown in dashed orange and fitted trace shown in red. Spectrum normalized to 1.

Peak Analysis

Data Set:[Book1]Sheet1!D Date:4/30/2025

BaseLine:Poly5

Chi^2=1.43233E-03 Adj. R-Square=9.70759E-01 # of Data Points=845

SS=1.12438E+00 Degrees of Freedom=785

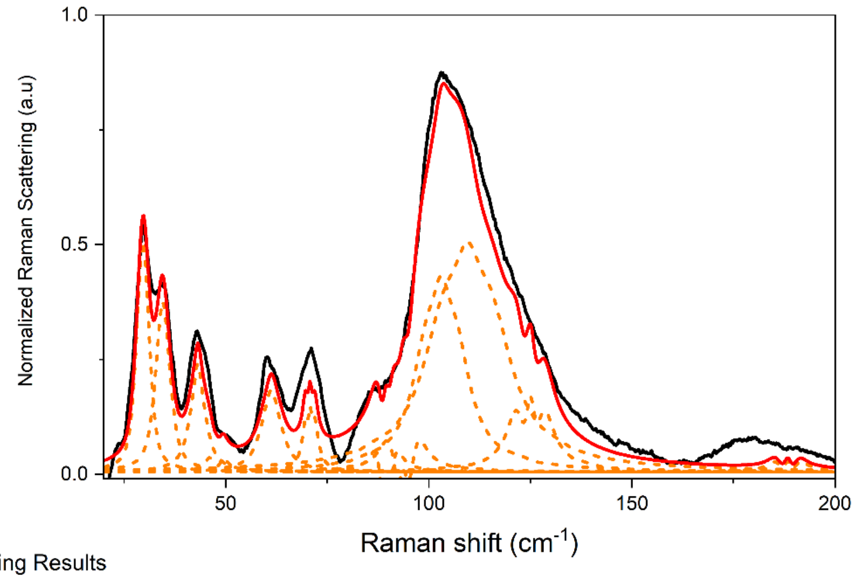

Fitting Results

Peak Index	Peak Type	Area Intg	FWHM	Max Height	Center Grvty	Area IntgP
1	PsdVoigt2	2.38635	3.68206	0.50197	29.6247	7.08541
2	PsdVoigt2	2.09334	4.19468	0.36771	34.54502	6.21542
3	PsdVoigt2	1.41189	4.14061	0.23594	43.15097	4.19209
4	PsdVoigt2	1.46677	5.68572	0.17841	61.25492	4.35505
5	PsdVoigt2	0.75697	4.01304	0.141	70.81966	2.24755
6	PsdVoigt2	0.34937	3.08316	0.04475	89.47607	1.03734
7	PsdVoigt2	0.95028	4.5643	0.06861	93.75198	2.8215
8	PsdVoigt2	7.5526	13.05458	0.42719	103.08306	22.42471
9	PsdVoigt2	13.67299	20.62623	0.50023	109.56081	40.59696
10	PsdVoigt2	2.48417	12.21686	0.16271	125.03327	7.37584
11	PsdVoigt2	0.25433	2.81472	0.03057	47.7751	0.75515
12	PsdVoigt2	0.30076	4.28825	0.0222	188.34261	0.893

Figure S17. Pseudo-Voigt Line-shape analysis fit 295 K Raman spectrum for pure DMDPU from 10-200 cm^{-1}. Experimental traces shown in black, fit peaks shown in dashed orange and fitted trace shown in red. Spectrum normalized to 1.

Peak Analysis

Data Set:[Book1]Sheet1!F		Date:4/30/2025
BaseLine:Constant		
Chi^2=4.42556E-05	Adj. R-Square=9.59183E-01	# of Data Points=495
SS=2.14640E-02	Degrees of Freedom=485	

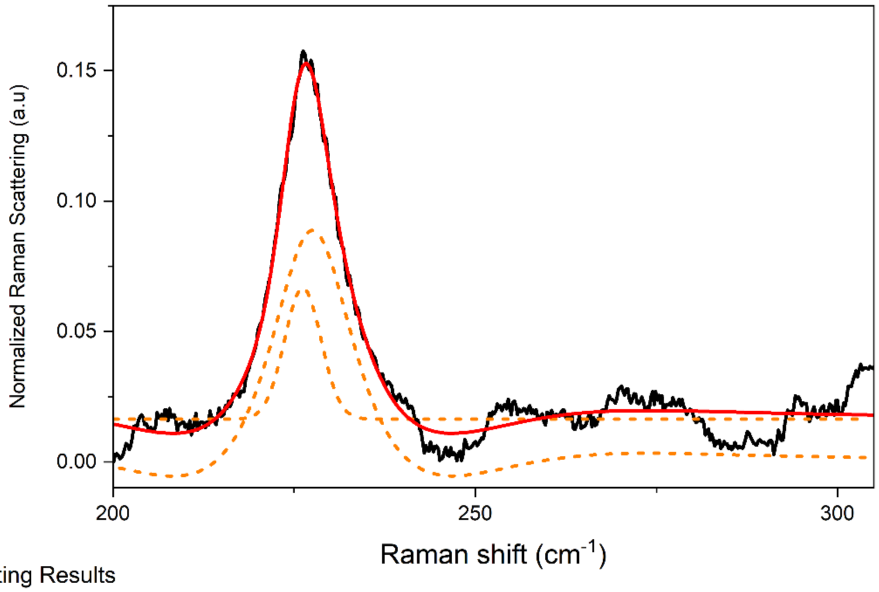

Fitting Results

Peak Index	Peak Type	Area Intg	FWHM	Max Height	Center Grvty	Area IntgP
1	PsdVoigt2	2.0512	7.40756	0.06655	226.20588	65.26794
2	PsdVoigt2	1.09154	11.64181	0.08871	227.52445	34.73206

Figure S18. Pseudo-Voigt Line-shape analysis fit 295 K Raman spectrum for pure DMDPU from 200-305 cm^{-1}. Experimental traces shown in black, fit peaks shown in dashed orange and fitted trace shown in red. Spectrum normalized to 1.

Peak Analysis

Data Set:[Book1]Sheet1!D　　Date:4/30/2025

BaseLine:Poly5

Chi^2=4.16460E-06　　Adj. R-Square=9.81633E-01　　# of Data Points=845

SS=3.14427E-03　　Degrees of Freedom=755

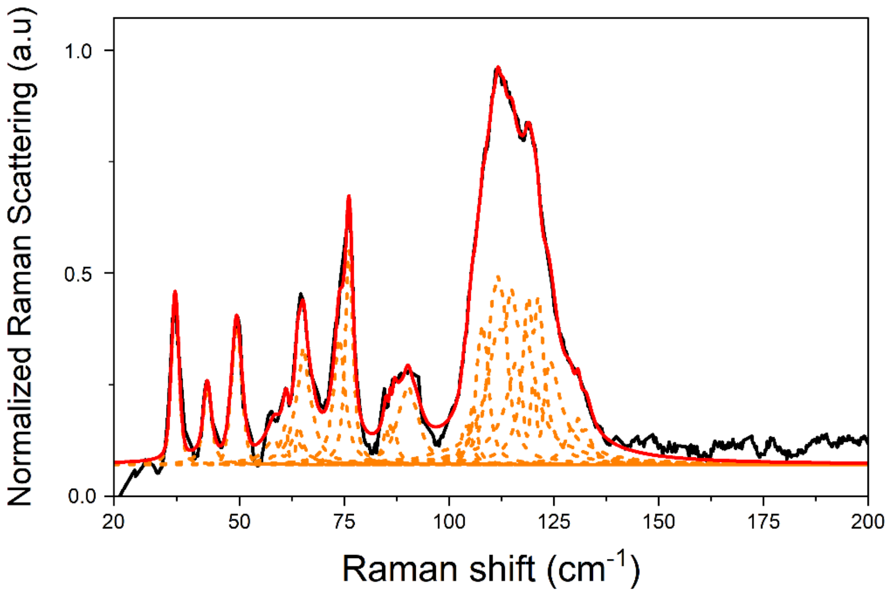

Peak Index	Peak Type	Area Intg	FWHM	Max Height	Center Grvty	Area IntgP
1	PsdVoigt2	0.07032	2.14934	0.02644	34.66838	3.47271
2	PsdVoigt2	0.04192	2.37335	0.01212	42.35361	2.07048
3	PsdVoigt2	0.07269	2.89522	0.02179	49.34499	3.58999
4	PsdVoigt2	0.03859	1.99049	0.00614	62.52038	1.90591
5	PsdVoigt2	0.10901	4.06205	0.01838	65.28419	5.38362
6	PsdVoigt2	0.10359	2.14897	0.03349	76.18866	5.11592
7	PsdVoigt2	0.02981	3.67329	0.0061	85.97052	1.47217
8	PsdVoigt2	0.10039	5.84655	0.01237	90.22715	4.95785
9	PsdVoigt2	0.21032	5.12756	0.02945	111.65898	10.38696
10	PsdVoigt2	0.39504	11.12938	0.02738	114.86022	19.50965
11	PsdVoigt2	0.27626	5.03369	0.01613	120.12891	13.64325
12	PsdVoigt2	0.21236	5.81919	0.02629	120.11095	10.48781
13	PsdVoigt2	0.04931	3.97995	0.00411	60.64865	2.43514
14	PsdVoigt2	0.0573	6.1715	0.00721	130.80314	2.82966
15	PsdVoigt2	0.02612	3.04984	0.00603	104.2353	1.28979
16	PsdVoigt2	0.03103	2.28402	0.00966	105.67814	1.53246
17	PsdVoigt2	0.10553	3.47127	0.02186	107.96817	5.21188
18	PsdVoigt2	0.09526	3.23973	0.01984	73.83979	4.70476

Figure S19. Pseudo-Voigt Line-shape analysis fit 78 K Raman spectrum for pure DMDPU from 10-200 cm^{-1}. Experimental traces shown in black, fit peaks shown in dashed orange and fitted trace shown in red. Spectrum normalized to 1.

Peak Analysis

Data Set:[Book1]Sheet1!F   Date:4/30/2025

BaseLine:Poly4

Chi^2=5.52596E-05   Adj. R-Square=9.53297E-01   # of Data Points=503

SS=2.60826E-02   Degrees of Freedom=472

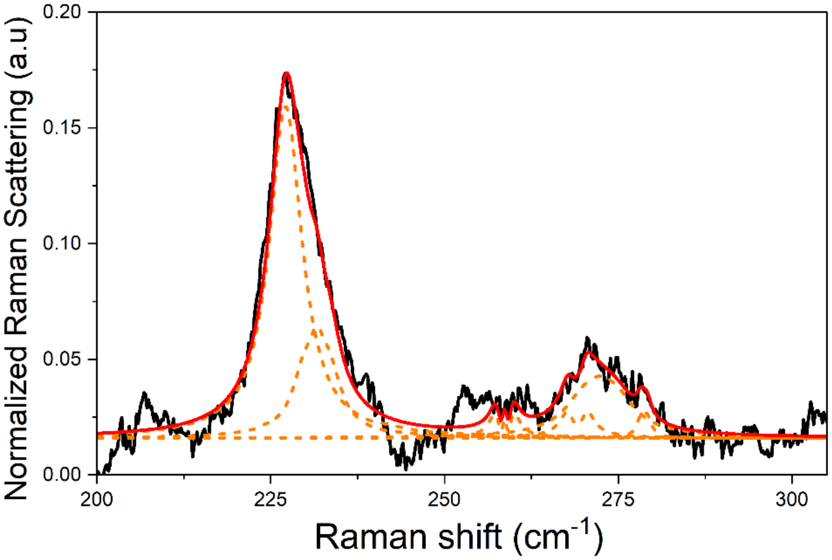

Peak Index	Peak Type	Area Intg	FWHM	Max Height	Center Grvty	Area IntgP
1	PsdVoigt2	1.23342	5.74413	0.14317	227.1611	56.17786
2	PsdVoigt2	0.41528	6.32735	0.04847	231.67101	18.91441
3	PsdVoigt2	0.06522	1.77815	0.01067	258.66003	2.97059
4	PsdVoigt2	0.08558	5.84656	0.01151	269.1166	3.89774
5	PsdVoigt2	0.3606	9.81368	0.02675	272.38079	16.42381
6	PsdVoigt2	0.03547	2.23793	0.01112	278.732	1.61561

Figure S20. Pseudo-Voigt Line-shape analysis fit 78 K Raman spectrum for pure DMDPU from 200-305 cm^{-1}. Experimental traces shown in black, fit peaks shown in dashed orange and fitted trace shown in red. Spectrum normalized to 1.

Peak Analysis

Data Set:[Book1]Sheet1!B"RT" Date:4/30/2025
BaseLine:Poly4
Chi^2=1.78366E-01 Adj. R-Square=9.62189E-01 # of Data Points=3282
SS=5.81650E+02 Degrees of Freedom=3261

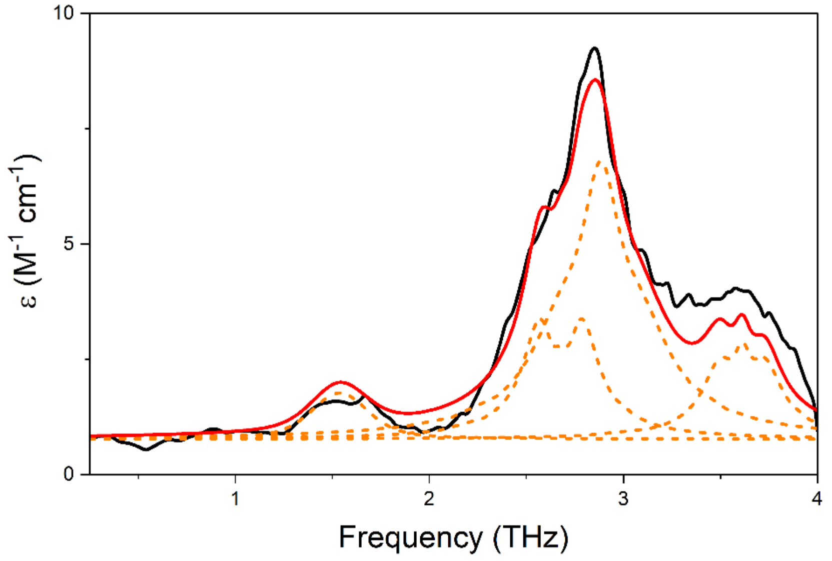

Peak Index	Peak Type	Area Intg	FWHM	Max Height	Center Grvty	Area IntgP
1	PsdVoigt2	0.45733	0.3515	1.00284	1.53809	6.68542
2	PsdVoigt2	1.51124	0.44866	2.61021	2.6778	22.09207
3	PsdVoigt2	3.82451	0.46833	6.0298	2.88398	55.90867
4	PsdVoigt2	1.04757	0.4178	2.09499	3.61341	15.31384

Figure S21. Pseudo-Voigt Line-shape analysis fit for 295 K THz spectrum of pure DEDPU. Experimental traces shown in black, fit peaks shown in dashed orange, and fitted trace shown in red.

Peak Analysis

Data Set:[Book1]Sheet1!D"78K" Date:4/30/2025

BaseLine:Poly4

Chi^2=2.35371E-02 Adj. R-Square=9.97150E-01 # of Data Points=3282

SS=7.65190E+01 Degrees of Freedom=3251

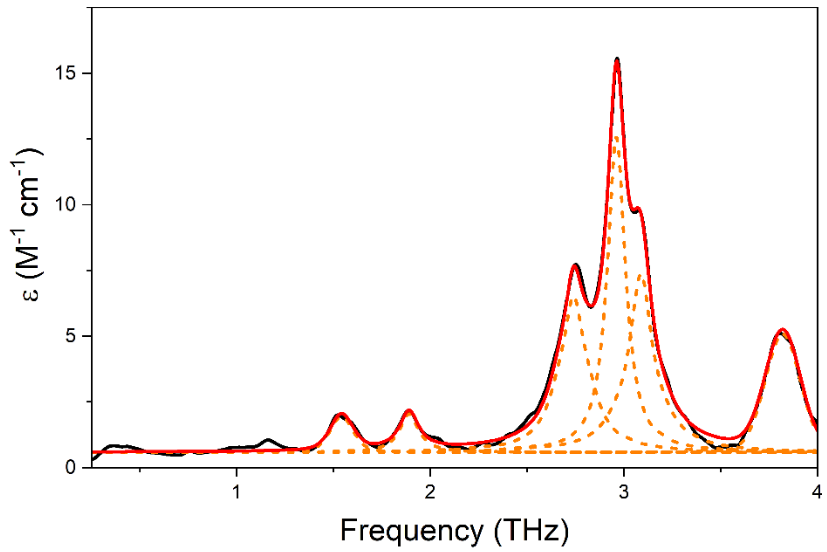

Peak Index	Peak Type	Area Intg	FWHM	Max Height	Center Grvty	Area IntgP
1	PsdVoigt2	0.24317	0.14107	1.36941	1.54223	3.52174
2	PsdVoigt2	0.26627	0.1187	1.45815	1.89065	3.85628
3	PsdVoigt2	1.56452	0.18157	5.94299	2.74073	22.65816
4	PsdVoigt2	2.06154	0.1137	11.97514	2.96139	29.8562
5	PsdVoigt2	1.71196	0.15367	6.78616	3.0881	24.7935
6	PsdVoigt2	1.05742	0.21559	4.49892	3.82108	15.31411

Figure S22. Pseudo-Voigt Line-shape analysis fit for 78 K THz spectrum of pure DEDPU. Experimental traces shown in black, fit peaks shown in dashed orange, and fitted trace shown in red.

Peak Analysis

Data Set:[Book1]Sheet1!B"RT" Date:4/30/2025

BaseLine:Poly5

Chi^2=2.13798E-02 Adj. R-Square=9.68141E-01 # of Data Points=3295

SS=6.96767E+01 Degrees of Freedom=3259

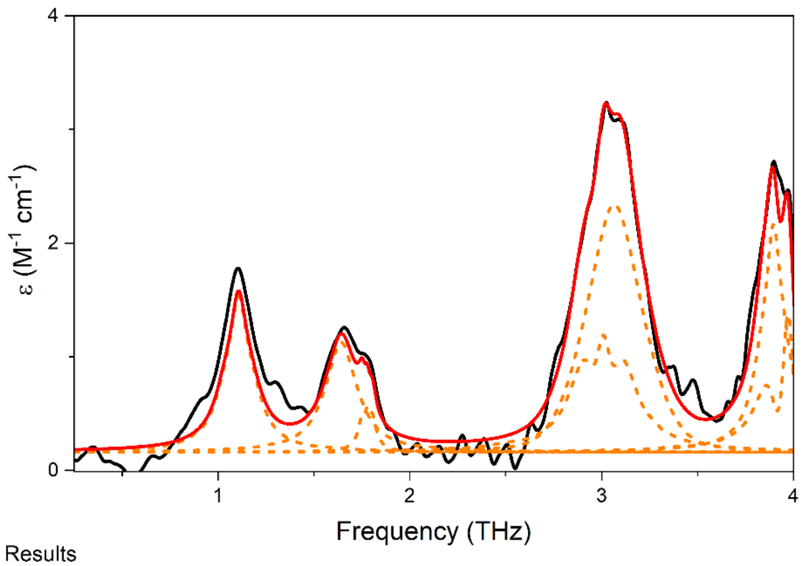

Fitting Results

Peak Index	Peak Type	Area Intg	FWHM	Max Height	Center Grvty	Area IntgP
1	PsdVoigt2	0.33588	0.16995	1.37586	1.10808	12.88528
2	PsdVoigt2	0.28935	0.20171	0.9655	1.64259	11.10048
3	PsdVoigt2	0.45787	0.3572	1.02902	3.00886	17.56546
4	PsdVoigt2	0.85902	0.33706	2.17899	3.06553	32.95494
5	PsdVoigt2	0.38506	0.1491	2.00259	3.89553	14.77196
6	PsdVoigt2	0.22834	0.06393	1.21672	3.97105	8.75983
7	PsdVoigt2	0.05114	0.0948	0.39472	1.77864	1.96204

Figure S23. Pseudo-Voigt Line-shape analysis fit for 295 K THz spectrum of pure DMDPU. Experimental traces shown in black, fit peaks shown in dashed orange, and fitted trace shown in red.

Peak Analysis

Data Set:[Book1]Sheet1!D"78K" Date:4/30/2025

BaseLine:Poly5

Chi^2=2.97961E-02 Adj. R-Square=9.81253E-01 # of Data Points=3295

SS=9.65096E+01 Degrees of Freedom=3239

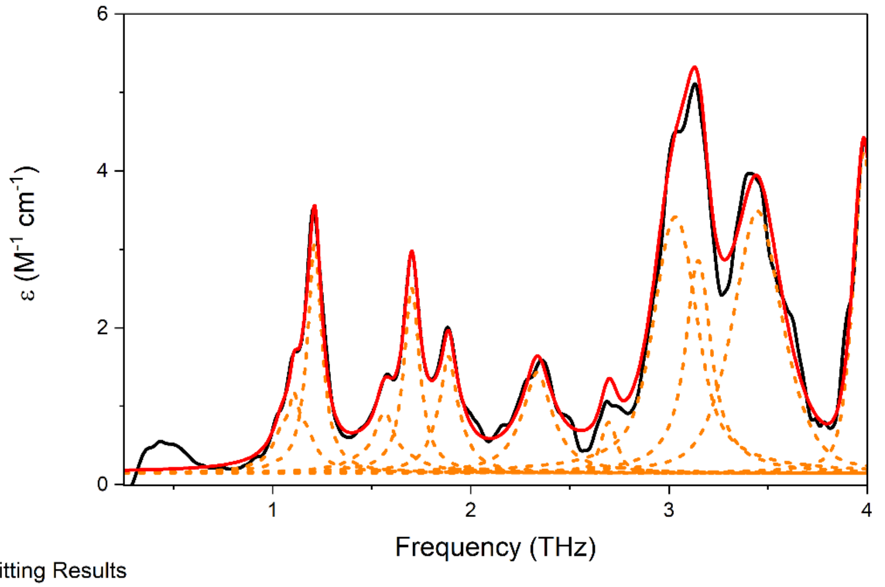

Fitting Results

Peak Index	Peak Type	Area Intg	FWHM	Max Height	Center Grvty	Area IntgP
1	PsdVoigt2	0.3948	0.08847	2.92476	1.21256	7.17862
2	PsdVoigt2	0.19785	0.14081	0.7766	1.56437	3.59742
3	PsdVoigt2	0.35084	0.09621	2.35348	1.70229	6.37917
4	PsdVoigt2	0.2775	0.12055	1.48413	1.88806	5.04577
5	PsdVoigt2	0.35682	0.18582	1.28448	2.33614	6.48802
6	PsdVoigt2	0.08911	0.09724	0.64376	2.69672	1.62034
7	PsdVoigt2	1.17739	0.26039	3.26608	3.03257	21.40809
8	PsdVoigt2	0.64956	0.15718	2.70573	3.14779	11.81082
9	PsdVoigt2	1.21389	0.29492	3.33952	3.44733	22.07173
10	PsdVoigt2	0.57032	0.12842	4.09713	3.98404	10.37003
11	PsdVoigt2	0.22164	0.1636	1.01874	1.10807	4.02999

Figure S24. Pseudo-Voigt Line-shape analysis fit for 78 K THz spectrum of pure DMDPU. Experimental traces shown in black, fit peaks shown in dashed orange, and fitted trace shown in red.

Peak Analysis

Data Set:[Book1]Sheet1!D Date:4/30/2025

BaseLine:Poly4

Chi^2=9.18805E-05 Adj. R-Square=9.96820E-01 # of Data Points=3294

SS=2.97509E-01 Degrees of Freedom=3238

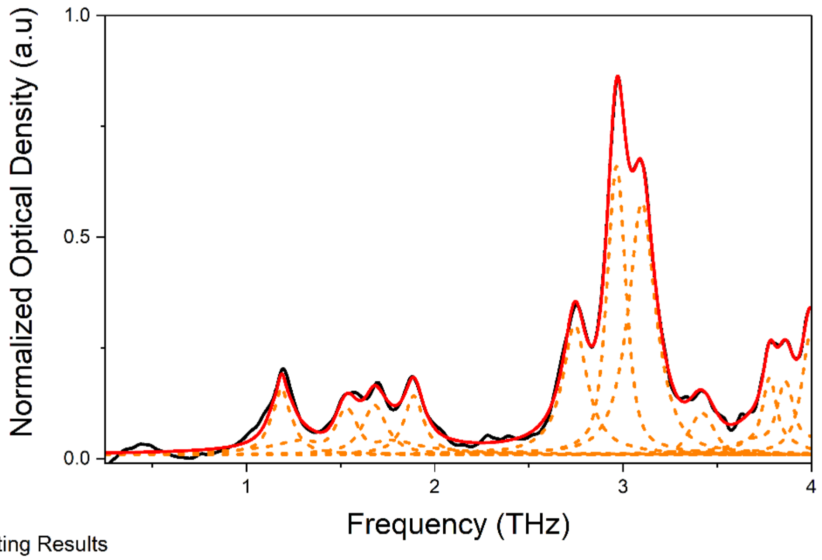

Fitting Results

Peak Index	Peak Type	Area Intg	FWHM	Max Height	Center Grvty	Area IntgP
1	PsdVoigt2	0.0267	0.12303	0.15165	1.1868	4.88078
2	PsdVoigt2	0.04437	0.14648	0.10462	1.53177	8.111
3	PsdVoigt2	0.02507	0.15314	0.11178	1.67996	4.58292
4	PsdVoigt2	0.02495	0.12479	0.13232	1.88884	4.56055
5	PsdVoigt2	0.06962	0.156	0.29354	2.74366	12.72788
6	PsdVoigt2	0.104	0.11916	0.65013	2.96504	19.01321
7	PsdVoigt2	0.13455	0.172	0.56918	3.10008	24.5993
8	PsdVoigt2	0.01737	0.15417	0.09345	3.41934	3.17516
9	PsdVoigt2	0.02837	0.09715	0.17294	3.77819	5.1862
10	PsdVoigt2	0.03616	0.11742	0.16461	3.86188	6.61003
11	PsdVoigt2	0.03584	0.11348	0.27017	3.99504	6.55297

Figure S25. Pseudo-Voigt Line-shape analysis fit for THz spectrum of equimolar mix DEDPU:DMDPU at 78 K. Experimental traces shown in black, fit peaks shown in dashed orange, and fitted trace shown in red. Spectrum normalized to 1.

Peak Analysis

Data Set:[Book1]Sheet1!D		Date:4/30/2025
BaseLine:Poly4		
Chi^2=5.63886E-04	Adj. R-Square=9.88189E-01	# of Data Points=880
SS=4.51109E-01	Degrees of Freedom=800	

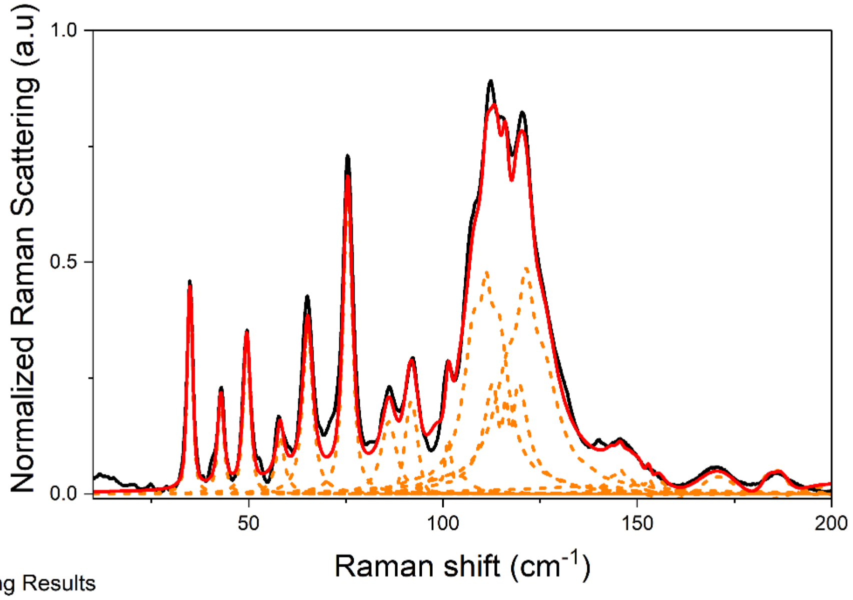

Fitting Results

Peak Index	Peak Type	Area Intg	FWHM	Max Height	Center Grvty	Area IntgP
1	PsdVoigt2	0.99532	1.74773	0.43652	34.89592	3.12297
2	PsdVoigt2	0.55625	1.95512	0.20068	42.82765	1.74532
3	PsdVoigt2	0.98072	2.23681	0.3279	49.51558	3.07718
4	PsdVoigt2	0.52936	2.55723	0.12579	58.08772	1.66097
5	PsdVoigt2	1.64873	3.12285	0.35516	65.17971	5.17318
6	PsdVoigt2	2.57487	2.84068	0.64951	75.49744	8.07911
7	PsdVoigt2	1.11316	4.8454	0.15553	86.06785	3.49274
8	PsdVoigt2	0.85882	3.73385	0.19945	91.85634	2.6947
9	PsdVoigt2	0.75799	1.95023	0.11925	101.37481	2.37832
10	PsdVoigt2	7.21215	11.34282	0.47814	111.10258	22.62936
11	PsdVoigt2	3.86029	12.72802	0.23998	116.14753	12.11231
12	PsdVoigt2	8.54192	13.40242	0.48654	121.37513	26.80172
13	PsdVoigt2	0.48084	6.85347	0.05302	145.57444	1.50871
14	PsdVoigt2	1.03361	8.76619	0.03667	170.80589	3.24313
15	PsdVoigt2	0.34165	6.90339	0.04974	185.38722	1.07197
16	PsdVoigt2	0.3851	8.99708	0.03547	152.90646	1.20831

Figure S26. Pseudo-Voigt Line-shape analysis fit for Raman spectrum of 0.10 mix DEDPU:DMDPU from 10-200 cm^{-1} at 78 K. Experimental traces shown in black, fit peaks shown in dashed orange, and fitted trace shown in red. Spectrum normalized to 1.

Peak Analysis

Data Set:[Book1]Sheet1!F
Date:4/30/2025
BaseLine:Poly5
Chi^2=4.26690E-05
Adj. R-Square=9.71638E-01
# of Data Points=503
SS=1.99264E-02
Degrees of Freedom=467

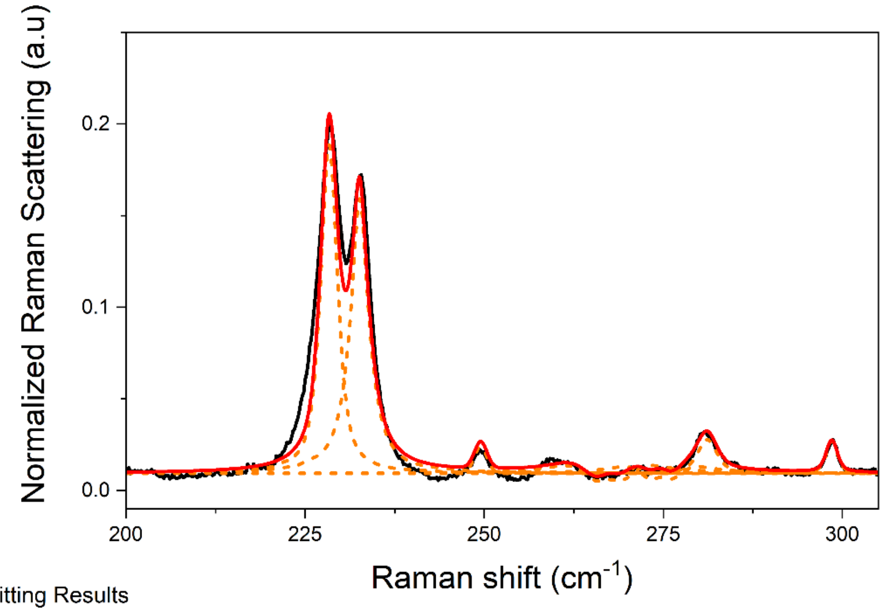

Fitting Results

Peak Index	Peak Type	Area Intg	FWHM	Max Height	Center Grvty	Area IntgP
1	PsdVoigt2	0.69645	2.82007	0.17897	228.35268	41.81878
2	PsdVoigt2	0.69481	3.02968	0.14975	232.61366	41.7201
3	PsdVoigt2	0.03405	2.064	0.01471	249.50746	2.04481
4	PsdVoigt2	0.06188	6.53927	0.00427	267.36326	3.71566
5	PsdVoigt2	0.04885	5.53047	0.00372	274.66216	2.93307
6	PsdVoigt2	0.04402	1.91027	0.01749	298.51319	2.64313
7	PsdVoigt2	0.08014	3.25567	0.01836	281.13431	4.81202

Figure S27. Pseudo-Voigt Line-shape analysis fit for Raman spectrum of 0.10 mix DEDPU:DMDPU from 200-305 cm^{-1} at 78 K. Experimental traces shown in black, fit peaks shown in dashed orange, and fitted trace shown in red. Spectrum normalized to 1.

Peak Analysis

Data Set:[Book1]Sheet1!D | Date:4/30/2025

BaseLine:Poly5

Chi^2=6.65742E-04 | Adj. R-Square=9.87299E-01 | # of Data Points=880

SS=5.28599E-01 | Degrees of Freedom=794

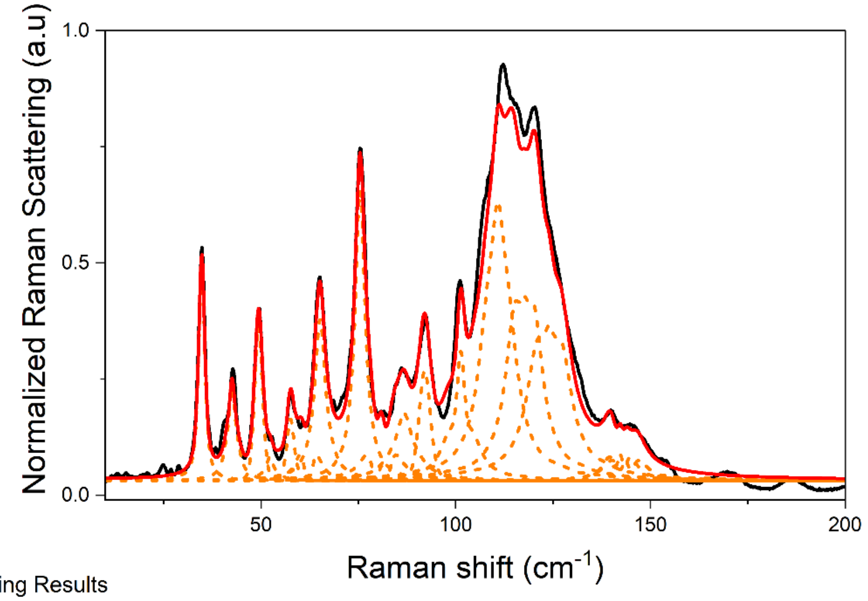

Fitting Results

Peak Index	Peak Type	Area Intg	FWHM	Max Height	Center Grvty	Area IntgP
1	PsdVoigt2	1.26826	1.90069	0.47329	34.79145	3.69256
2	PsdVoigt2	0.80635	2.63025	0.20131	42.58467	2.34769
3	PsdVoigt2	0.92126	2.42937	0.33042	49.39104	2.68225
4	PsdVoigt2	0.23583	2.54221	0.05149	52.32294	0.68662
5	PsdVoigt2	0.41959	2.22984	0.13238	57.59472	1.22164
6	PsdVoigt2	0.51718	2.90401	0.05634	62.18375	1.50577
7	PsdVoigt2	1.92265	3.47979	0.34647	65.18797	5.59782
8	PsdVoigt2	3.11506	3.17351	0.62345	75.43115	9.06954
9	PsdVoigt2	0.80471	4.36225	0.05576	80.90412	2.34292
10	PsdVoigt2	1.40695	6.12271	0.14448	86.76138	4.09635
11	PsdVoigt2	0.98531	3.42653	0.23748	92.05361	2.86874
12	PsdVoigt2	2.12495	3.24558	0.27779	101.29672	6.1868
13	PsdVoigt2	7.03956	8.45962	0.60192	110.82393	20.49575
14	PsdVoigt2	5.5719	12.23172	0.335	124.00548	16.22263
15	PsdVoigt2	0.68289	9.79856	0.05561	142.2213	1.98824
16	PsdVoigt2	0.71918	13.02153	0.04408	143.60066	2.09391
17	PsdVoigt2	5.80481	10.50444	0.39702	117.56639	16.90077

Figure S28. Pseudo-Voigt Line-shape analysis fit for Raman spectrum of 0.25 mix DEDPU:DMDPU from 10-200 cm^{-1} at 78 K. Experimental traces shown in black, fit peaks shown in dashed orange, and fitted trace shown in red. Spectrum normalized to 1.

Peak Analysis

Data Set:[Book1]Sheet1!F Date:4/30/2025

BaseLine:Poly5

Chi^2=4.03351E-05 Adj. R-Square=9.73475E-01 # of Data Points=503

SS=1.90382E-02 Degrees of Freedom=472

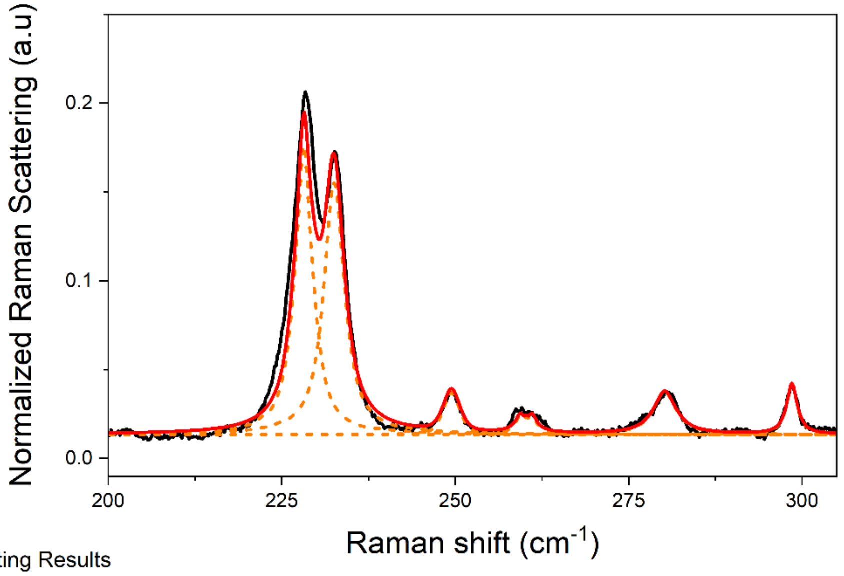

Fitting Results

Peak Index	Peak Type	Area Intg	FWHM	Max Height	Center Grvty	Area IntgP
1	PsdVoigt2	0.73692	3.05629	0.1627	228.17626	40.04446
2	PsdVoigt2	0.74996	3.55177	0.14149	232.54786	40.75312
3	PsdVoigt2	0.07712	1.84855	0.02845	298.52006	4.19086
4	PsdVoigt2	0.05136	3.48215	0.01074	260.22322	2.79089
5	PsdVoigt2	0.08363	2.83408	0.02347	249.47002	4.54451
6	PsdVoigt2	0.14126	3.95965	0.02409	280.20327	7.67616

Figure S29. Pseudo-Voigt Line-shape analysis fit for Raman spectrum of 0.25 mix DEDPU:DMDPU from 200-305 cm^{-1} at 78 K. Experimental traces shown in black, fit peaks shown in dashed orange, and fitted trace shown in red. Spectrum normalized to 1.

Peak Analysis

Data Set:[Book1]Sheet1!D Date:4/30/2025
BaseLine:Poly4
Chi^2=1.12553E-04 Adj. R-Square=9.97332E-01 # of Data Points=880
SS=8.76791E-02 Degrees of Freedom=779

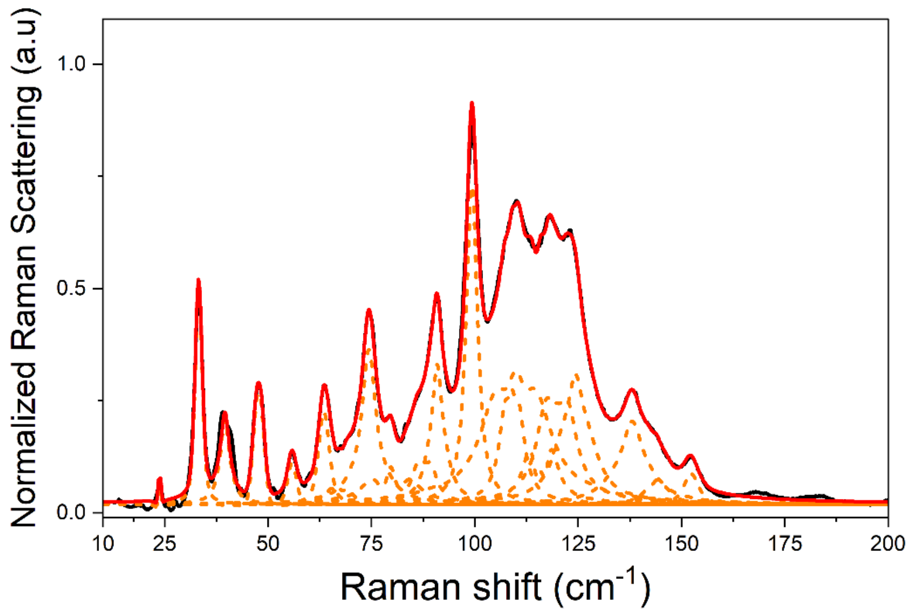

Peak Index	Peak Type	Area Intg	FWHM	Max Height	Center Grvty	Area IntgP
1	PsdVoigt2	1.26324	2.05352	0.48545	33.19668	3.55886
2	PsdVoigt2	0.80084	2.74097	0.19089	39.56295	2.25617
3	PsdVoigt2	0.72087	2.89856	0.24903	47.71276	2.03088
4	PsdVoigt2	0.30106	2.51165	0.09226	55.79132	0.84816
5	PsdVoigt2	0.85771	3.16223	0.20459	63.52563	2.41639
6	PsdVoigt2	0.36098	7.44413	0.03547	67.17657	1.01697
7	PsdVoigt2	2.54749	4.34438	0.34462	74.38403	7.17693
8	PsdVoigt2	1.24982	3.24909	0.08295	79.68601	3.52106
9	PsdVoigt2	1.87982	3.81547	0.31121	90.90849	5.29594
10	PsdVoigt2	0.92617	6.92571	0.09449	87.75176	2.60926
11	PsdVoigt2	3.5152	3.29844	0.71088	99.28431	9.90323
12	PsdVoigt2	7.00746	17.86491	0.29831	109.95136	19.74183
13	PsdVoigt2	1.52615	10.10187	0.12191	115.90511	4.29957
14	PsdVoigt2	2.29701	6.49186	0.25582	108.96814	6.47127
15	PsdVoigt2	3.61083	10.91821	0.23785	119.84311	10.17265
16	PsdVoigt2	3.44645	8.43366	0.28947	124.37344	9.70955
17	PsdVoigt2	2.27761	7.89413	0.18492	138.1094	6.41662
18	PsdVoigt2	0.40014	4.30265	0.06797	152.34113	1.12731
19	PsdVoigt2	0.02686	0.87202	0.04883	23.77217	0.07566
20	PsdVoigt2	0.47978	6.56181	0.05431	144.07868	1.35168

Figure S30. Pseudo-Voigt Line-shape analysis fit for Raman spectrum of 0.50 mix DEDPU:DMDPU from 10-200 cm^{-1} at 78 K. Experimental traces shown in black, fit peaks shown in dashed orange, and fitted trace shown in red. Spectrum normalized to 1.

Peak Analysis

Data Set:[Book1]Sheet1!F Date:4/30/2025

BaseLine:Poly4

Chi^2=2.34135E-05 Adj. R-Square=9.68022E-01 # of Data Points=503

SS=1.09341E-02 Degrees of Freedom=467

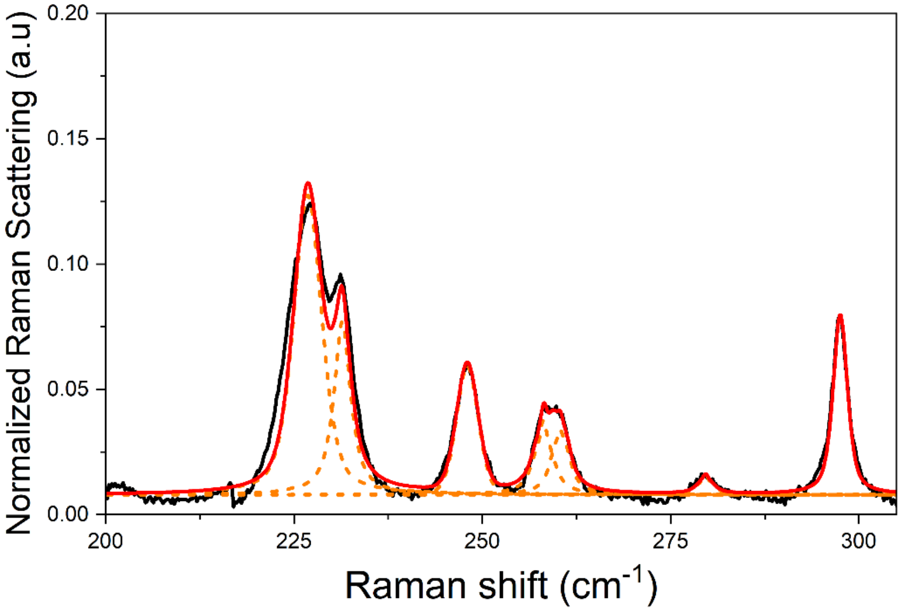

Peak Index	Peak Type	Area Intg	FWHM	Max Height	Center Grvty	Area IntgP
1	PsdVoigt2	0.67615	4.4383	0.11952	226.81169	42.22107
2	PsdVoigt2	0.27323	2.69238	0.06909	231.41153	17.06145
3	PsdVoigt2	0.20083	3.40349	0.05126	248.01206	12.54032
4	PsdVoigt2	0.11209	2.6334	0.0298	258.12984	6.99931
5	PsdVoigt2	0.1	2.83322	0.02553	260.38359	6.24427
6	PsdVoigt2	0.21507	2.26756	0.07175	297.52864	13.42955
7	PsdVoigt2	0.02409	2.12436	0.00778	279.6395	1.50403

Figure S31. Pseudo-Voigt Line-shape analysis fit for Raman spectrum of 0.50 mix DEDPU:DMDPU from 200-305 cm^{-1} at 78 K. Experimental traces shown in black, fit peaks shown in dashed orange, and fitted trace shown in red. Spectrum normalized to 1.

Peak Analysis

Data Set:[Book1]Sheet1!D　　　Date:4/30/2025

BaseLine:Poly4

Chi^2=2.86795E-05　　　Adj. R-Square=9.98565E-01　　　# of Data Points=880

SS=2.24847E-02　　　Degrees of Freedom=784

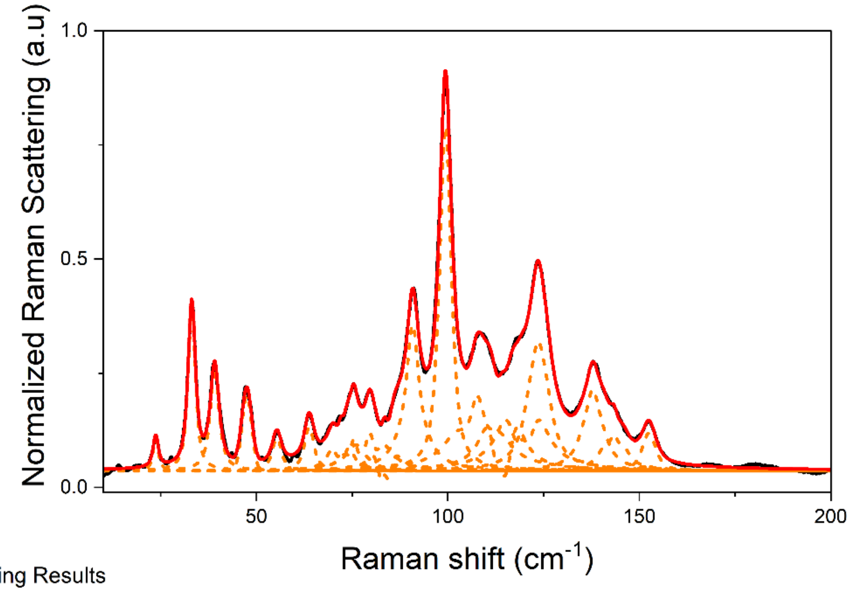

Fitting Results

Peak Index	Peak Type	Area Intg	FWHM	Max Height	Center Grvty	Area IntgP
1	PsdVoigt2	0.17618	1.70637	0.06951	23.71041	0.72244
2	PsdVoigt2	1.17183	2.31029	0.36069	33.12842	4.80515
3	PsdVoigt2	0.91374	2.74366	0.22476	39.11193	3.74682
4	PsdVoigt2	0.53014	3.01667	0.16498	47.4918	2.17387
5	PsdVoigt2	0.31717	2.91506	0.0721	55.33492	1.30056
6	PsdVoigt2	0.43362	2.97575	0.09635	63.79109	1.77809
7	PsdVoigt2	0.5266	8.19845	0.04997	72.00288	2.15936
8	PsdVoigt2	0.55249	5.69672	0.06888	75.46168	2.2655
9	PsdVoigt2	1.10455	11.27664	0.08153	79.6158	4.52925
10	PsdVoigt2	0.33006	3.51059	0.03298	83.50713	1.35344
11	PsdVoigt2	2.36654	4.39875	0.3194	90.80258	9.70408
12	PsdVoigt2	3.90194	3.66752	0.7533	99.35165	16.00003
13	PsdVoigt2	2.70944	12.59889	0.16343	107.70075	11.11016
14	PsdVoigt2	1.47251	5.09403	0.09475	114.15353	6.03807
15	PsdVoigt2	2.33441	16.90508	0.11134	119.46185	9.57234
16	PsdVoigt2	2.68992	6.6882	0.28405	123.61914	11.03011
17	PsdVoigt2	1.57805	6.02522	0.17843	137.8437	6.47086
18	PsdVoigt2	0.48594	4.27919	0.08261	152.52817	1.9926
19	PsdVoigt2	0.79192	7.56226	0.07942	143.35435	3.24729

Figure S32. Pseudo-Voigt Line-shape analysis fit for Raman spectrum of 0.75 mix DEDPU:DMDPU from 10-200 cm^{-1} at 78 K. Experimental traces shown in black, fit peaks shown in dashed orange, and fitted trace shown in red. Spectrum normalized to 1.

Peak Analysis

Data Set:[Book1]Sheet1!F

Date:4/30/2025

BaseLine:Poly5

Chi^2=1.04881E-05

Adj. R-Square=9.71785E-01

# of Data Points=503

SS=4.96085E-03

Degrees of Freedom=473

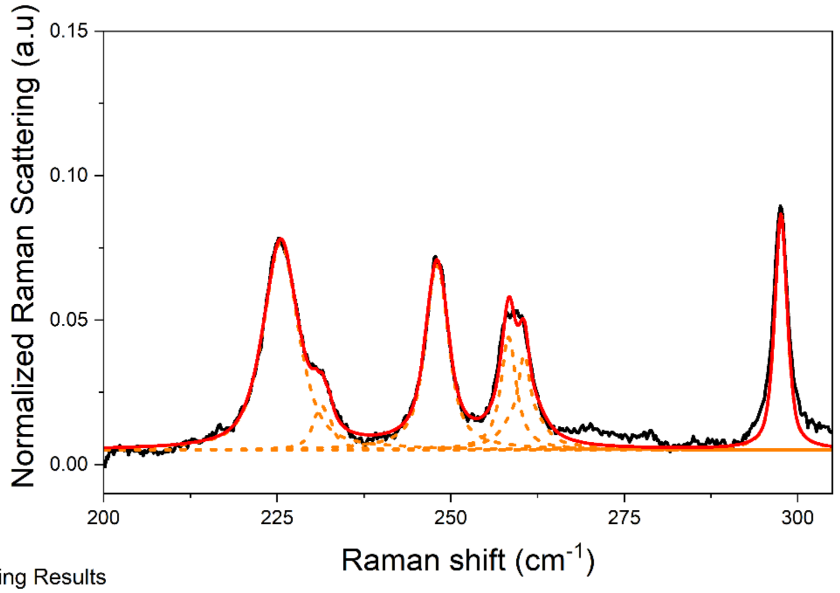

Fitting Results

Peak Index	Peak Type	Area Intg	FWHM	Max Height	Center Grvty	Area IntgP
1	PsdVoigt2	0.57039	5.96458	0.0719	225.51336	36.6012
2	PsdVoigt2	0.06448	3.1244	0.01514	231.52464	4.13761
3	PsdVoigt2	0.36447	3.99864	0.06383	248.01996	23.38787
4	PsdVoigt2	0.16064	2.68035	0.039	258.34398	10.30814
5	PsdVoigt2	0.16444	2.74514	0.03306	260.59038	10.55187
6	PsdVoigt2	0.23397	2.15925	0.08132	297.5741	15.0133

Figure S33. Pseudo-Voigt Line-shape analysis fit for Raman spectrum of 0.75 mix DEDPU:DMDPU from 200-305 cm^{-1} at 78 K. Experimental traces shown in black, fit peaks shown in dashed orange, and fitted trace shown in red. Spectrum normalized to 1.

Peak Analysis

Data Set:[Book1]Sheet1!D Date:4/30/2025

BaseLine:Poly4

Chi^2=4.51978E-04 Adj. R-Square=9.81056E-01 # of Data Points=880

SS=3.58871E-01 Degrees of Freedom=794

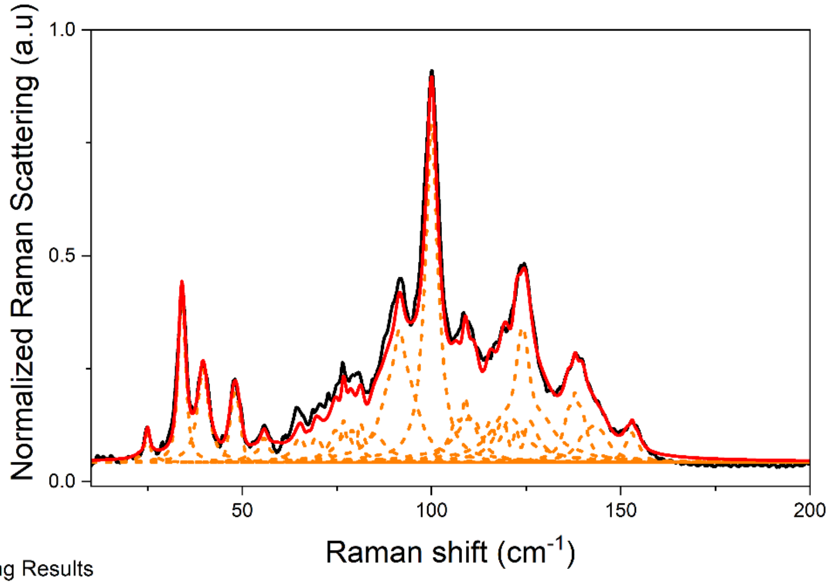

Fitting Results

Peak Index	Peak Type	Area Intg	FWHM	Max Height	Center Grvty	Area IntgP
1	PsdVoigt2	0.21208	1.67618	0.06655	24.91538	0.8338
2	PsdVoigt2	1.227	2.35467	0.37483	34.03832	4.82395
3	PsdVoigt2	1.12733	3.579	0.2057	39.64586	4.43208
4	PsdVoigt2	0.54056	3.18325	0.15473	48.11082	2.12519
5	PsdVoigt2	0.3856	3.27657	0.054	55.7528	1.51598
6	PsdVoigt2	0.61071	8.42045	0.05705	67.39724	2.40101
7	PsdVoigt2	0.81892	7.25015	0.09257	76.7527	3.21959
8	PsdVoigt2	3.29064	8.06507	0.2949	91.54165	12.93712
9	PsdVoigt2	4.60127	4.04065	0.7481	100.00475	18.08983
10	PsdVoigt2	1.32324	7.33537	0.144	108.93081	5.20229
11	PsdVoigt2	2.45843	7.72213	0.09854	115.44568	9.66529
12	PsdVoigt2	3.63378	5.60959	0.30028	124.04333	14.28618
13	PsdVoigt2	1.43853	4.58146	0.09233	122.3529	5.65557
14	PsdVoigt2	1.2208	5.89684	0.15409	137.90684	4.79955
15	PsdVoigt2	0.96601	8.73517	0.08036	142.74259	3.79787
16	PsdVoigt2	0.37278	4.21117	0.06581	153.01255	1.46559
17	PsdVoigt2	1.20797	2.96431	0.08399	81.28803	4.74912

Figure S34. Pseudo-Voigt Line-shape analysis fit for Raman spectrum of 90-10 mix DEDPU:DMDPU from 10-200 cm^{-1} at 78 K. Experimental traces shown in black, fit peaks shown in dashed orange, and fitted trace shown in red. Spectrum normalized to 1.

Peak Analysis

Data Set:[Book1]Sheet1!F | Date:4/30/2025
BaseLine:Poly5
Chi^2=2.57600E-05 | Adj. R-Square=9.33819E-01 | # of Data Points=503
SS=1.21845E-02 | Degrees of Freedom=473

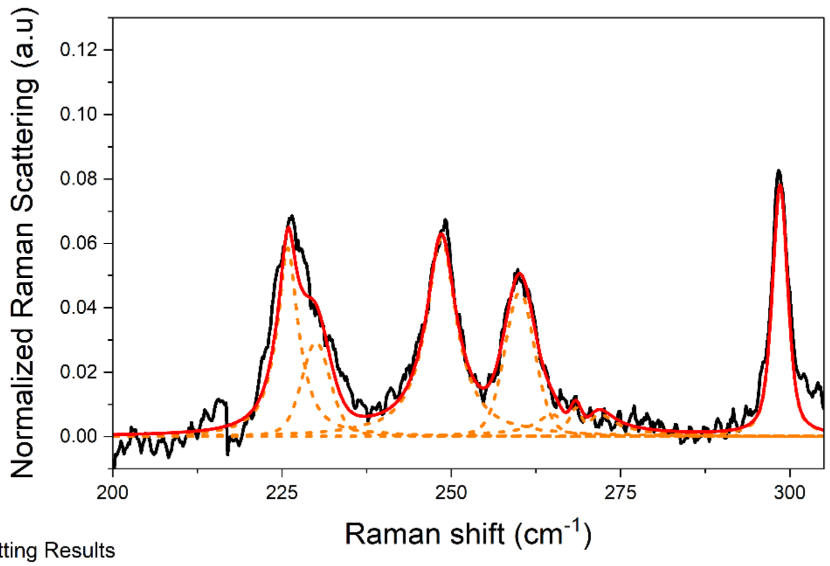

Fitting Results

Peak Index	Peak Type	Area Intg	FWHM	Max Height	Center Grvty	Area IntgP
1	PsdVoigt2	0.26617	2.67828	0.07772	298.55151	15.85152
2	PsdVoigt2	0.11742	2.70929	0.00893	268.4137	6.99296
3	PsdVoigt2	0.27335	5.23413	0.04478	260.10434	16.27914
4	PsdVoigt2	0.51029	5.43079	0.06151	248.5285	30.3897
5	PsdVoigt2	0.33725	3.80121	0.05852	225.85827	20.08449
6	PsdVoigt2	0.17467	4.959	0.02936	230.02455	10.4022

Figure S35. Pseudo-Voigt Line-shape analysis fit for Raman spectrum of 90-10 mix DEDPU:DMDPU from 200-305 cm^{-1} at 78 K. Experimental traces shown in black, fit peaks shown in dashed orange, and fitted trace shown in red. Spectrum normalized to 1.

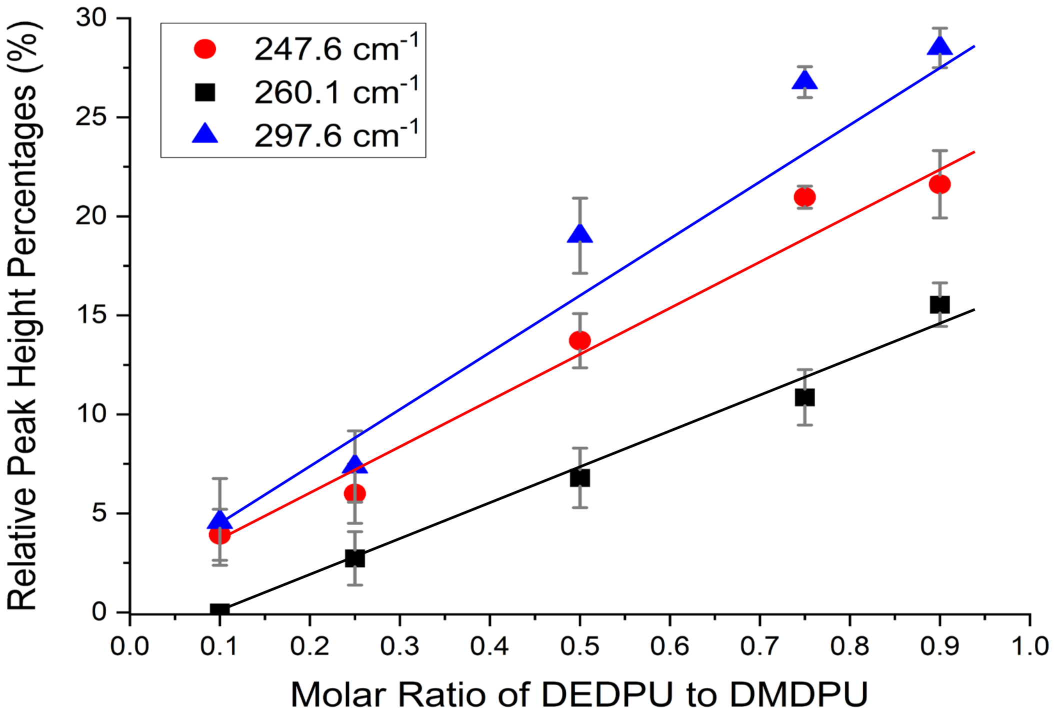

Figure S36. Depiction of key peaks and their relative peak height percentages across each mixture composition for the 200-305 cm^{-1} range. The LOD for 247.6 cm^{-1} is 0.17. The LOD for 260.1 cm^{-1} is 0.13. The LOD for 297.6 cm^{-1} is 0.17.

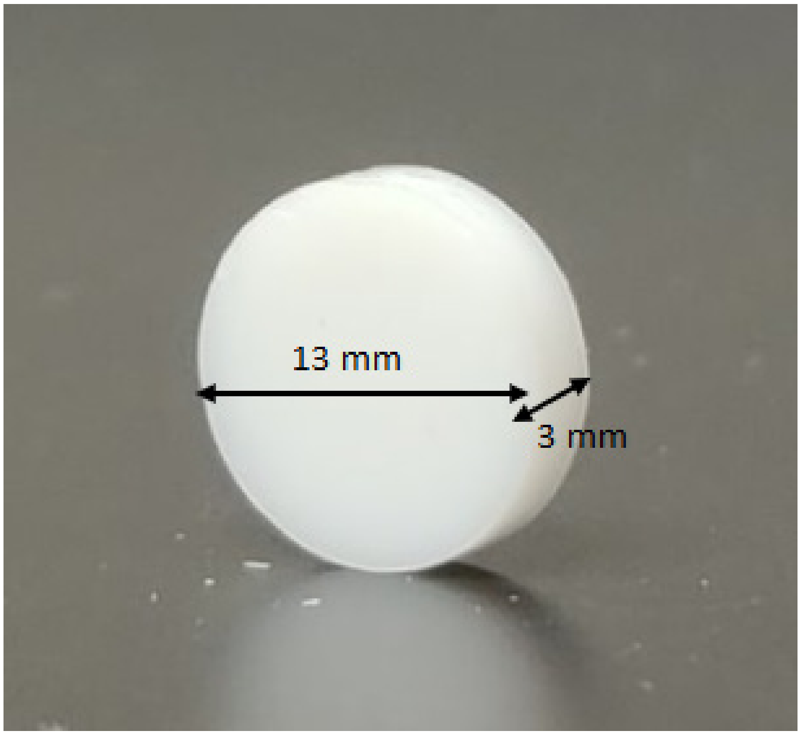

Figure S37. Standard appearance and dimensions of a THz pellet with PTFE as the matrix. A pure PTFE pellet of 13 mm by 3.0 mm was used as reference. Sample pellet information for DEDPU is as follows: 3% DEDPU:PTFE resulting in a 13 mm diameter by 3.2 mm thick pellet. Sample pellet information for DMDPU is as follows: 3% DMDPU:PTFE resulting in a 13 mm diameter by 3.3 mm thick pellet.
